# The adaptability of grassland soil microbiomes to resource and stress shifts is mainly accomplished by niche conservatism under nitrogen deposition

**DOI:** 10.1093/ismeco/ycaf215

**Published:** 2025-11-20

**Authors:** Qing-Yi Yu, Xin Liu, Hui Yao, Peng-Peng Lü, Guo-Jiao Yang, Xiao-Tao Lü, Xing-Guo Han, Liang-Dong Guo, Ying Huang

**Affiliations:** State Key Laboratory of Microbial Diversity and Innovative Utilization, Institute of Microbiology, Chinese Academy of Sciences, No.1 Beichen West Road, Chaoyang District, Beijing 100101, China; College of Life Sciences, University of Chinese Academy of Sciences, No. 19, Yuquan Road, Shijingshan District, Beijing 100049, China; State Key Laboratory of Microbial Diversity and Innovative Utilization, Institute of Microbiology, Chinese Academy of Sciences, No.1 Beichen West Road, Chaoyang District, Beijing 100101, China; College of Life Sciences, University of Chinese Academy of Sciences, No. 19, Yuquan Road, Shijingshan District, Beijing 100049, China; State Key Laboratory of Microbial Diversity and Innovative Utilization, Institute of Microbiology, Chinese Academy of Sciences, No.1 Beichen West Road, Chaoyang District, Beijing 100101, China; Marine Agriculture Research Center, Tobacco Research Institute of Chinese Academy of Agricultural Sciences, No. 11 Keyuanjing 4th Road, Laoshan District, Qingdao 266101, China; State Key Laboratory of Microbial Diversity and Innovative Utilization, Institute of Microbiology, Chinese Academy of Sciences, No.1 Beichen West Road, Chaoyang District, Beijing 100101, China; Zhejiang Key Laboratory for Restoration of Damaged Coastal Ecosystems, School of Life Sciences, Taizhou University, No. 1139 Shifu Road, Jiaojiang district, Taizhou 318000, China; Hainan Baoting Tropical Rainforest Ecosystem Observation and Research Station, School of Ecology, Hainan University, No. 58 Renmin Avenue, Meilan District, Haikou 570228, China; Erguna Forest-Steppe Ecotone Research Station, CAS Key Laboratory of Forest Ecology and Management, Institute of Applied Ecology, Chinese Academy of Sciences, No. 72 Wenhua Road, Shenhe District, Shenyang 110016, China; Erguna Forest-Steppe Ecotone Research Station, CAS Key Laboratory of Forest Ecology and Management, Institute of Applied Ecology, Chinese Academy of Sciences, No. 72 Wenhua Road, Shenhe District, Shenyang 110016, China; School of Life Sciences, Institute of Life Science and Green Development, Hebei University, No. 180 Wusi East Road, Baoding 071002, China; State Key Laboratory of Vegetation and Environmental Change, Institute of Botany, Chinese Academy of Sciences, No. 20 Nanxincun, Xiangshan, Haidian District, Beijing 100093, China; State Key Laboratory of Microbial Diversity and Innovative Utilization, Institute of Microbiology, Chinese Academy of Sciences, No.1 Beichen West Road, Chaoyang District, Beijing 100101, China; College of Life Sciences, University of Chinese Academy of Sciences, No. 19, Yuquan Road, Shijingshan District, Beijing 100049, China; State Key Laboratory of Microbial Diversity and Innovative Utilization, Institute of Microbiology, Chinese Academy of Sciences, No.1 Beichen West Road, Chaoyang District, Beijing 100101, China; College of Life Sciences, University of Chinese Academy of Sciences, No. 19, Yuquan Road, Shijingshan District, Beijing 100049, China

**Keywords:** life history strategies, soil microbiome, resources and stress, niche conservatism, nitrogen deposition, grassland, adaptability, metagenomes

## Abstract

Atmospheric nitrogen (N) deposition usually alters the ratio of resources to stress in terrestrial ecosystems and has important impacts on soil microbiomes. To elucidate the adaptability of soil microbiomes under N deposition scenarios, we conducted a 6-year N addition experiment in a temperate grassland in Inner Mongolia, applying different levels of ammonium nitrate (AN) and urea (AU) to form different resource-to-stress ratio. Our results reveal that the inborn high yield (Y)-resource acquisition (A)-stress tolerance (S) life history strategies of soil microbiomes collectively drive their adaptability to resources and stress under N deposition. Enriched taxa under AN treatment mainly belonged to *Actinomycetota* and *Chloroflexota* with Y and S strategies, while those under AU mainly belonged to *Pseudomonadota* with A and S strategies. Functional preference analysis indicated that bacterial phyla maintained consistent Y-A-S life history strategies across AN and AU treatments. Moreover, strong purifying selection restricted the pace of adaptive evolution, and horizontal gene transfer expanded the functional repertoire in a complementary rather than essential manner. Thus, the adaptation of microbiomes to shifting resources and stress under N deposition scenarios is mainly accomplished by niche conservatism (“move”) rather than niche evolution (“evolve”). Our results support the point that it may be easier for microbial species to move into a befitting niche than to evolve to acclimate a new environment.

## Introduction

The earth is now undergoing profound global environmental changes, marked by climate warming, ocean acidification, altered biogeochemical cycles, and biodiversity loss [1]. Among these, atmospheric nitrogen (N) deposition is a particularly pressing challenge of the Anthropocene, with far-reaching consequences for ecosystem health, greenhouse gas balance, and global biodiversity [2–4]. By inducing eutrophication and acidification, N deposition fundamentally reshapes local environments through altering the ratio of resources to stress [5, 6].

Soil microorganisms play vital roles in ecosystem functioning and are highly sensitive to global environmental changes [7, 8]. Although many studies have documented the variation of microbial community structures under various global change drivers [9–11], microbial communities are considered resistant, resilient, and functionally redundant [12]. As a result, community-level structural metrics alone may not be sensitive enough to reflect the ecosystem processes under changing conditions [13–15]. Microbial traits, as collective features of microbiomes, can reflect their adaptability to environmental changes at the levels of specific taxa and even the whole community [16–18]. For example, the genome size of oligotrophic stress-tolerators in harsh environments is larger than that of copiotrophic non-tolerators in benign environments, due to the need to cope with abiotic stress and limited resources [19]. This suggests that trait-based approaches are promising to reveal microbial life history strategies and integrate microbial processes into ecosystem models under global changes [20, 21].

According to the trait-based Y-A-S theory, soil microbes exhibit three major life history strategies in response to varying resources and stress: high yield (Y), resource acquisition (A), and stress tolerance (S) [22]. Microbial taxa with different life history strategies are favored by different environmental conditions [19, 22, 23]. S-strategy bacteria can resist ammonia (NH_3_) toxicity by increasing lipopolysaccharide biosynthesis and promoting biofilm formation [24], while A-strategy bacteria with motility and chemotaxis capabilities effectively compete for limited resources [25]. Besides, Ning *et al.* proved that Y-strategy microbial functions, such as amino acid and carbohydrate metabolism, are more abundant in low-salinity soils than in high-salinity environments [26]. All these indicate that the Y-A-S framework would be conducive to linking the changing microbial taxa and functional potentials, and then to summarizing the adaptive microbial traits. However, the trait-based adaptability of microbiomes remains unexplored in the context of N deposition.

Although the trait-based theory can reflect the environmental adaptability of microbes, little attention has been paid to how microbiomes develop corresponding traits to cope with altered environmental conditions. Generally, when confronting environmental changes, species may either track their original niches through dispersal (niche conservatism, or “move”) or adapt to new niches through evolution (niche evolution, or “evolve”) [27, 28]. Nevertheless, such studies on adaptive trait development have largely been confined to macroorganisms. For instance, the *Vitaceae* mainly took “move” strategy during the Eocene Climatic Optimum and Miocene Climatic Optimum, for the pervasive high global temperature providing appropriate and similar empty niches [29]. Whereas in the Miocene, the expand of colder and drier environments provided new, *in situ* niches, which might promote niche shifts and more frequent “evolve” events in *Vitaceae* [29]. Recently, trait-based frameworks are increasingly employed to characterize microbial ecological strategies; however, these efforts largely depend on presumed traits imputed from taxonomic identity using existing literature or databases [30–32]. This approach tacitly assumes that adaptive traits are acquired by “move”. However, microbial traits often deviate from taxonomic relatedness owing to horizontal gene transfer (HGT) and other evolutionary mechanisms [17]. For example, Yang *et al.* found that both continuous and ceased N addition increased bacterial HGT events and the richness of horizontally transferred genes, resulting in the mismatch between bacterial taxonomic and functional diversities [33]. This suggests that “evolve” may promote diversification and help bacteria to resist variable soil conditions. All the above highlight the need to disentangle the relative contributions of “move” and “evolve” to microbial adaptability, particularly under intensifying global change.

**Figure 1:**
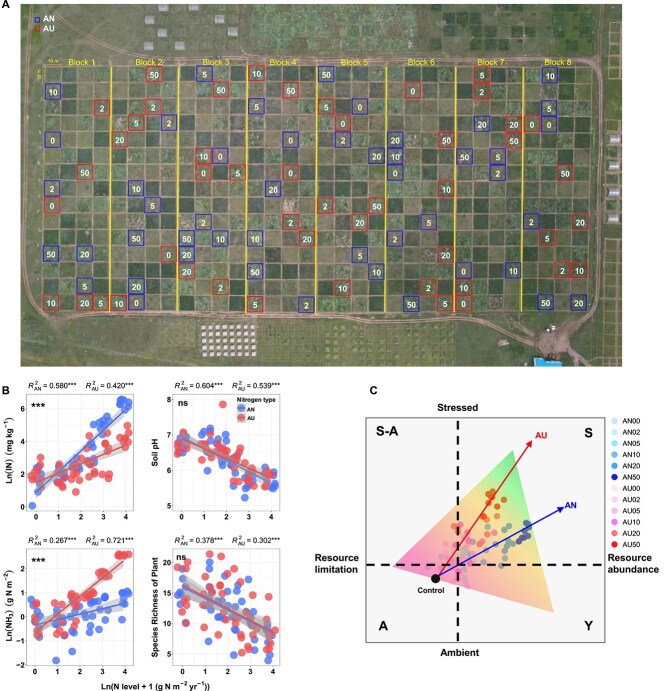
Experimental design and conceptual model. (A) Spatial distribution of eight nitrogen (N) addition blocks. Each block includes 60 plots (10 × 10 m^2^) arranged in completely randomized block design. Plots sampled in this study (*n* = 12 per block) are enclosed with blue and red rectangles, representing ammonium nitrate addition (AN) and urea addition (AU), respectively. Numbers inside the rectangles indicate six N addition levels (0, 2, 5, 10, 20, and 50 g N m^−2^ yr^−1^). Samples from each plot were subjected to 16S amplicon, ITS amplicon, and metagenome shotgun sequencing (Fig. S1). (B) Effects of AN and AU addition on resource availability and stress. Resource availability was measured by soil available N (IN). Biotic stress was measured by plant diversity, and abiotic stress by soil pH and NH_3_ toxicity (using NH_3_ volatilization as a proxy, Fig. S3). Statistical analysis was performed using a linear regression model with two-sided test, and adjusted R^2^ values are reported. The grey shaded area around the smooth line indicates the 95% confidence interval. The marks in the top left corner indicate the significance of the difference between the regression slopes of AN and AU (ns, non-significant; ^***^, *P* < .001). (C) Conceptual model depicting the interaction of resources and stress based on Y-A-S life history strategies. This figure was adapted from the conceptual framework of Malik *et al.* [22], in which the x- and y-axes represent resource availability and stress intensity, respectively. To better reflect actual conditions under AN and AU treatments, the framework was combined with the stress-to-resource ratio patterns derived from Fig. S2, indicated by the blue (AN) and red (AU) arrows.

In this study, we aim to elucidate the adaptability of soil microbiomes to shifting resources and stress under N deposition scenarios and to uncover the underlying mechanisms. To this end, we applied ammonium nitrate (AN) and urea (AU) as N treatments, common approaches for simulating N deposition-induced environmental changes [34, 35]. While both fertilizers relieve N limitation by increasing soil available N, they also exacerbate survival stress such as soil acidification, loss of plant species, and NH_3_ toxicity. Notably, AU induces stronger NH_3_ toxicity but provides lower N availability than AN, due to its higher NH_3_ volatilization potential [36–38]. The accumulated NH_3_ gas passively diffuses into microbial cells, where it disrupts cellular metabolism by impairing intracellular pH regulation and inhibiting enzymatic activities [39, 40], thereby directly threatening microbial survival. Based on the above rationales, we conducted a 6-year simulated N deposition experiment in a temperate grassland in Inner Mongolia, employing two N types (AN and AU) and six N levels (0, 2, 5, 10, 20, and 50 g N m^−2^ yr^−1^) (Fig. 1A). Using both metabarcoding and shotgun metagenomic data, we analyzed bacterial community composition, associated microbial traits, and the evolutionary ecology of functional genes (Fig. S1). Soil available N, pH, NH_3_ toxicity, and plant species richness were measured to characterize resource and stress gradients under AN and AU treatments (Fig. 1B, C; Fig. S2). The results demonstrated that the difference in resources and stress caused by AN and AU addition drove divergent microbiome assembly patterns, underpinned by the inborn Y-A-S life history strategies of the constituent taxa. Our findings also support the ecological view that “it is easier to move than to evolve” [41], indicating a predominant microbial reliance on prioritizing niche tracking over evolutionary adaptation in response to N deposition.

## Materials and methods

### Study site and sampling

Field experiments were conducted at a temperate grassland in Inner Mongolia (50°10′46.1″N, 119°22′56.4″E, 523 m above sea level), which was built by the Erguna Forest-Steppe Ecotone Research Station, Institute of Applied Ecology, Chinese Academy of Sciences. The N is usually considered as the only limited resource [42]. The annual mean temperature is −2.45°C (1957–2016) and the annual mean precipitation is 363 mm, occurring mostly from May to August [34, 35]. The mean annual N deposition was less than 2.0 g N m^−2^ yr^−1^ for the last 20 years [43].

The N addition experiment was started in 2014 using a completely randomized block design. All plots were homogeneous at initiation of the experiment. In this study, we chose two N types (AN and AU), six N levels (0, 2, 5, 10, 20 and 50 g N m^−2^ yr^−1^), and an unmown management regime (Fig. 1). Note that AN00 and AU00 served as independent control treatments for the AN and AU series, respectively. On 22 July 2020, five soil cores (3 cm in diameter) were collected from the surface bulk soil (0–10 cm depth) of each plot (10 m × 10 m). These cores were combined into one composite sample per plot and sieved through a 2 mm mesh. The sieved soil was then immediately placed in sterile plastic bags, labeled, stored on ice and transported to the laboratory. In total, there were 96 bulk soil samples in this study. All samples were stored at −80°C until DNA extraction.

### Chemical analyses

The physical and chemical properties of soil and plants was derived from Yang et al (2022 & 2023) [34, 35] (Fig. 1B). The NH_3_ toxicity was estimated based on a meta-analysis (Fig. S3 and Table S1). See the statistical analyses for details. We used data on soil pH, soil available N, NH_3_ toxicity, and plant species richness, which form the general environmental conditions for soil microbiomes and are easily altered by N addition, to depict the different states of resources and stress under AN and AU treatments (Figs. 1C and S2).

### DNA extraction and sequencing

Genomic DNA of 0.5 g frozen soil samples were extracted using the Powersoil® DNA Isolation Kit (MoBio Laboratories Inc., Carlsbad, CA, USA) with the manufacturer’s instructions.

After quality check, the eligible DNA samples were processed to construct metagenome shotgun sequencing libraries with insert sizes of ~400 bp by using Illumina TruSeq Nano DNA LT Library Preparation Kit (Illumina, USA). Each library was sequenced by Illumina NovaSeq platform (Illumina, USA) with PE150 run mode at Personal Biotechnology Co., Ltd. (Shanghai, China). After data quality control, we got 1.3 TB data of clean read sequences for the 96 samples with average of 14 GB per sample.

The same DNA samples were diluted to 10 ng μl^-1^, then the bacterial 16S rRNA gene and fungal ITS rRNA gene was amplified with the primer pairs 515F and 806R, and ITS4F and fITS7, respectively. A 12-base pair barcode was on the primers 806R and ITS4F to differentiate each sample. The amplification for bacteria and fungi was carried out in 25 μl reaction solutions. After purifying and adding sequencing adapter to the PCR products using an Illumina TruSeq DNA PCR-Free Library Preparation Kit (Illumina) following the manufacturer’s instructions, the library was sequenced on an Illumina NovaSeq PE250 instrument (San Diego, CA, USA) at Chengdu Institute of Biology, Chinese Academy of Sciences.

### Metabarcoding analyses

Taxonomic identification of each 16S rRNA OTU and ITS rRNA OTU was performed using SINTAX algorithm with a confidence cutoff (*P*) value of .65 [44]. The Ribosomal Database Project [45] and UNITE database [46] were used as reference databases for bacteria and fungi, respectively. Then we removed the OTUs classified as “*Cyanobacteria*/*Chloroplast*”, “Mitochondria”, “Unclassified” and “Archaea” (only amounting to 3.39% of total sequences).

### Metagenome analyses

The cleans reads were assembled into contigs using MEGAHIT (v1.2.9) [47]. The GC% of target contigs was calculated using Quast (v5.2.0) [48].

To analyze bacterial functional potential, we combined all contigs and run redundans pipeline (https://github.com/lpryszcz/redundans) to remove redundant contigs. The protein-coding genes predicted by Prodigal [49] were clustered into non-redundant gene catalog with 147 656 673 genes by CD-HIT (v4.8.1), with identity threshold 0.95 and alignment coverage 0.9 [50]. The gene abundance of every sample was estimated by Salmon (v1.6.0) [51]. And the non-redundant gene catalog was annotated by EggNOG-mapper tool (v5.0) through against Kyoto Encyclopedia of Genes and Genomes (KEGG) database [52]. The ARG were annotated by DIAMOND tool (https://github.com/bbuchfink/diamond) through against Resfams database [53]. As we found that the gene numbers of phage in each sample were less than 1% of the total prokaryotic genes (Fig. S4) and the numbers of eukaryotic contigs in most samples (95 of the 96 samples) were less than 1% of the total contigs (Fig. S5), we considered the detected protein-coding genes mainly represented the functional potential and functional gene diversity (S.KO) of the bacterial community.

The evolutionary ecology of functional genes was assessed by profiling the intra-population genetic diversity of microbial community under each N treatment using the inStrain pipeline [54]. Metagenomic short reads were aligned to 8238 soil-derived reference genomes [55] to quantify nucleotide diversity (*π*) and pN/pS ratios. HGT events were detected from assembled contigs in each sample using the WAFFLE pipeline (http://huttenhower.sph.harvard.edu/waafle), which employs homology-based search to identify recently transferred genes. The number of HGT events was standardized by normalizing total HGT events against assembly sizes [56]. The transferred genes were extracted and annotated with KEGG database. The frequency of transferred genes in each sample was calculated.

Metagenome-assembled genomes (MAGs) was binned and integrated with MetaWRAP (v1.2.1) [57]. The MAGs were clustered using dRep (v3.4.2) [58], and the quality of MAGs was evaluated using CheckM (v1.2.2) [59]. The taxonomic classification of MAGs was done by GTDB-tk (v1.7.0) [60] and quantified by MetaWRAP, then the phylogenetic tree of high-quality MAGs (completeness > 80% and contamination < 5%) was constructed using GTDB-tk and visualized using iTOL (https://itol.embl.de/). The functional genes of each high-quality MAG were predicted using Prodigal and annotated with KEGG database. The frequency of genes in each MAG was calculated.

Average genome size was calculated by using MicrobeCensus pipeline [61]. This method calculated the sequence coverage of 30 core single-copy genes, which were ubiquitous in bacteria and archaea. The detection ratio of these essential genes should make up a higher proportion in the microbial community with a smaller genome size. Average 16S rRNA gene copy number was evaluated by referring the work of Pereira-Flores *et al.* [62]. In brief, the average 16S rRNA gene copy number was estimated by dividing the coverage of 16S rRNA gene by the number of genomes in an unassembled metagenome. gRodon (v2.3.0) [63] was used to estimate the minimal doubling time, which is closely related to bacterial maximum growth rate, by calculating codon usage bias. These indexes were calculated based on contigs > 500 bp for more accuracy of estimation methods.

### Statistical analyses

All the statistical analyses were implemented in R version 3.6.2 and 4.0.4, except the LEfSe analysis, which used online tool (huttenhower.sph.harvard.edu/galaxy).

We assigned KEGG pathways and functions of interest to Y-A-S life history strategies based on classifications established in previous studies, as summarized in Table S2. In cases where a pathway was associated with multiple Y-A-S categories, we applied analytical reasoning and contextual interpretation to either retain all relevant categories or select the most appropriate one. The relevant KEGG Orthologs (KOs) were subsequently assigned to the same Y-A-S strategies as their parent KEGG pathways. A complete summary of all KOs, their corresponding KEGG pathways, and assigned Y-A-S categories is provided in Table S4.

The NH_3_ toxicity was estimated through a meta-analysis of 18 studies that measured NH_3_ volatilization in response to the application of AN and AU (Fig. S3 and Table S1). To ensure data comparability, we selected studies that simultaneously applied both AN and AU fertilizers and measured NH_3_ volatilization within the same experimental site, encompassing a variety of ecosystems and soil textures. For each study, we recorded the N application rate and the corresponding NH_3_ volatilization measurements under both AN and AU treatments. We then constructed separate linear regression models between N application level and NH_3_ volatilization values for AN and AU using the data extracted from the meta-analysis. Based on these models, we estimated the likely NH_3_ volatilization at each N application level for both fertilizers using the predict and runif functions in the “*stats*” package [64]. The estimated value served as a proxy for NH₃ toxicity in the soil environment.

The differences of bacterial community and functional potential between AN and AU were detected using Differential analysis and visualized by Volcano plot in the “*edgeR*” package [65] and “*EnhancedVolcano*” package [66]. Then the significantly different KOs were implemented functional enrichment analysis based on KEGG database. The hypergeometric test in the “*stats*” package was used to test whether the enrichment ratio of a KEGG pathway had a P-value < .05.

The Random forest models (RFMs) predicted the important drivers for bacterial community, functional potential and microbial traits using the “*rfPermute*” [67], “*randomForest*” [68], and “*rfUtilities*” [69] packages. Besides, we used Spearman’s correlation analysis to test the relationship between Non-metric multidimensional scaling (NMDS) dimensions and relative abundance of bacteria taxa or functional genes in the “*psych*” package [70]. To test whether there was potential consistency between bacteria taxa, functional potential and microbial traits, we did Procrustes analysis using the “*vegan*” package [71]. Bacterial MAGs/KOs preference analysis between AN and AU of each phylum was carried out according to Toju *et al.* [72] with “*bipartite*” package [73].

Multitable co-inertia analysis (MCOA) was performed using the “*omicade4*” package [74] to identify consistent trait associations across soil bacterial communities, bacterial functional potential, and microbial traits under both AN and AU treatments. RFMs were used to predict the important drivers for the first two dimensions of MCOA. The contribution of each parameter to these dimensions was evaluated using multitable linear regression models with relative importance assessment in the “*relaimpo*” [75] packages.

In summary, niche conservatism (“move”) was assessed by comparing functional preferences and microbial traits of the same bacterial phylum existing in both AN and AU, followed by summarization through bacterial MAGs/KOs preference analysis. Niche evolution (“evolve”) was evaluated based on population microdiversity of functional genes and HGT events, particularly focusing on the functions of the transferred genes (Fig. S1).

More details of the materials and methods are provided in the Supplementary Information.

## Results

### The different ratios of resources to stress under AN and AU treatments led to divergent microbiome assembly patterns

**Figure 2:**
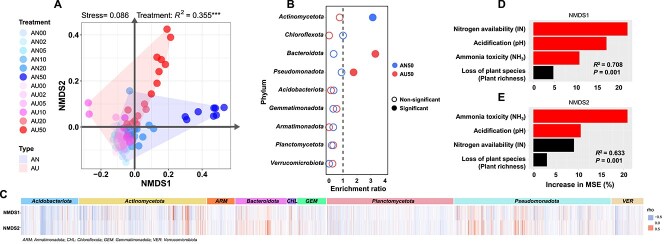
Enrichment patterns of soil bacterial taxa under AN and AU treatments. (A) NMDS (non-metric multidimensional scaling) analysis of bacterial community composition with Bray–Curtis dissimilarity and permutational analysis of variance (PERM ANOVA). (B) The enrichment analysis of bacterial community at the phylum level. The solid circles indicate the categories for which the enrichment has a P-value < .05 in a hypergeometric test. (C) Association between NMDS dimension and each OTU of the dominant bacterial phyla (*n* = 6075 OTUs). Note that associations between environmental variables and dominant bacterial phyla were detailed in Fig. S8. (D, E) Importance of resources and stress represented as the percentage of increase in %MSE (increase of mean square error) with **D** NMDS dimension1 and **E** dimension2 predicted by random forest models. Red bars indicate factors that significantly predict NMDS dimensions and black bar indicate non-significant predicting factors.

NMDS analysis for bacterial community composition using 16S rRNA gene amplicon data showed significant differences between AN and AU addition (*R*^2^ = 0.355, *P* < .001, Fig. 2A; Figs. S6 and S7). The enriched OTUs in AN50 mainly belonged to *Actinomycetota* and *Chloroflexota*, while those in AU50 mainly belonged to *Bacteroidota* and *Pseudomonadota* (Fig. 2B; Figs. S6 and S7). Correlation analysis between bacterial dominant phyla and NMDS dimensions derived from bacterial community composition revealed that OTUs of *Actinomycetota* and *Chloroflexota* were more strongly positively correlated with NMDS1, while those of *Bacteroidota* and *Pseudomonadota* showed a closer association with NMDS2 (Fig. 2C). RFMs indicated that the most important driver for NMDS1 was N availability (Fig. 2D) and that for NMDS2 was NH_3_ toxicity (Fig. 2E). These results suggest that the variation of resources and stress drove the different enrichment patterns of bacterial community between AN and AU treatments. Note that analysis of the bacterial community using metagenomic data got almost the same results (Figs. S9–S11).

**Figure 3:**
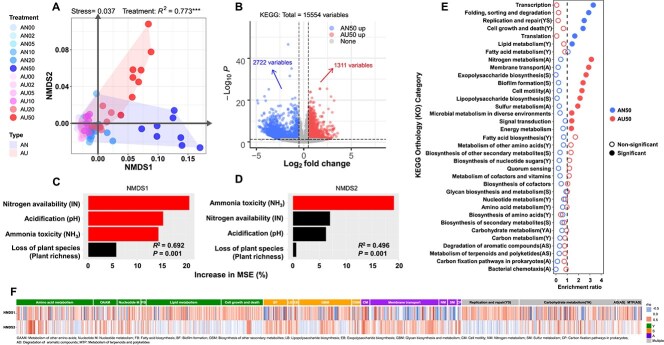
Enrichment patterns of soil bacterial functional potential (KOs) under AN and AU treatments. (A) NMDS analysis of bacterial functional potential with Bray–Curtis dissimilarity and PERM ANOVA under AN and AU treatment. (B) Volcano plot showing the different enrichment patterns of KOs between AN50 and AU50 (|LogFC| > 0.5, *P* < .05). (C, D) Importance of resources and stress represented as the percentage of increase in %MSE with **C** NMDS dimension1 and **D** dimension2 predicted by random forest models. Red bars indicate factors that significantly predict NMDS dimensions and black bar indicate non-significant predicting factors. (E) Functional enrichment analysis of up regulated KOs in AN50 and AU50 with hypergeometric tests. The functions were classified into Y-A-S life history strategies based on Table S2. Y, High yield; A, resource acquisition; S, stress tolerance. The solid circles indicate the categories for which the enrichment has a P-value < .05 in a hypergeometric test. (F) Association between NMDS dimension and functional potential classified into Y-A-S strategies (*n* = 1405 KOs). Note that associations between environmental variables and functional potential were detailed in Fig. S13.

Similarly, NMDS and differential analysis also showed significant differences in bacterial functional potential, annotated by the KEGG database, between AN and AU (*R*^2^ = 0.773, *P* < .001, Fig. 3A; |LogFC| > 0.5, *P* < .05, Fig. 3B). RFMs further indicated that the most important drivers for NMDS1 and NMDS2 derived from bacterial functional potential were still N availability and NH_3_ toxicity, respectively (Fig. 3C, D). Then, we classified the functional potential into Y-A-S life history strategies based on previous literature (Table S2). The enrichment analysis further showed that the functions of Y and S strategies tended to be enriched in AN50, while the functions of A and S strategies tended to be enriched in AU50 (Figs. 3E and S12). The correlation analysis showed that the KOs of the Y strategy were mainly positively correlated with NMDS1 but negatively correlated with NMDS2, while the KOs of the A strategy were mainly positively correlated with NMDS2 but negatively correlated with NMDS1 (Fig. 3F). Interestingly, for the S-strategy KOs, some (replication and repair) were mainly positively correlated with NMDS1 but negatively correlated with NMDS2, while others (biofilm formation and lipopolysaccharide biosynthesis) were mainly positively correlated with NMDS2 but negatively correlated with NMDS1 (Fig. 3F). This indicated that the microbiomes probably faced different stress along NMDS dimensions. All these results suggested that the variation of resources and stress were also the main drivers for the different patterns of functional potential between AN and AU treatment.

**Figure 4:**
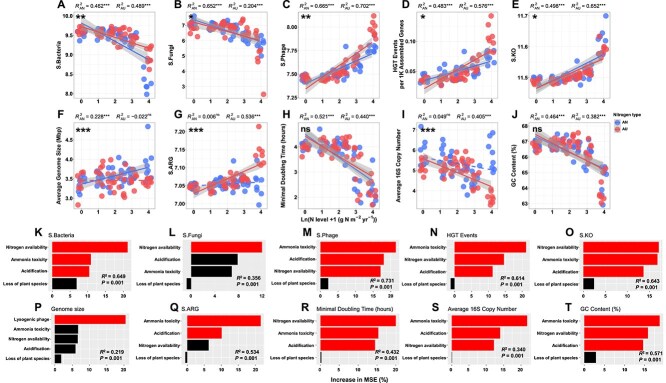
Microbial traits in relation to nitrogen addition. (A–J) The variation trend of 10 other microbial traits with increasing N level under AN and AU addition. Note that the taxonomy diversity of bacteria annotated from metagenomic data got almost the same results (Fig. S14). Taxonomy diversities of bacteria (S.Bacteria), fungi (S.Fungi), and phage (S.Phage), the diversities of bacterial antibiotic resistance genes (S.ARG), and prokaryotic functional gene diversities (S.KO) were measured by Shannon’s index. Bacterial estimated growth rate was measured by minimal doubling time. Statistical analysis was performed using a linear regression model with two-sided test, and adjusted R^2^ values are reported. The grey shaded area around the smooth line indicates the 95% confidence interval. The marks in the top left corner indicate the significance of the difference between the regression slopes of AN and AU (ns, non-significant; ^*^, .01 < *P* < .05; ^**^, .001 < *P* < .01; ^***^, *P* < .001). The dash lines indicate non-significantly relationships between microbial traits and N addition levels. (K–T) The importance of resources and stress represented as the percentage of increase in %MSE with microbial traits predicted by random forest models. Red bars indicate factors that significantly predict microbial traits, and black bars indicate non-significant predicting factors.

To comprehensively illustrate the response of microbiomes to AN and AU addition, we calculated 10 other microbial traits (Fig. 4) and compared them with previous studies in Table S3. We noted that the variation trends of microbial traits were not always the same between AN and AU. For example, the average genome size was only enlarged in AN but remained stable in AU (Fig. 4F). Also, the diversity of bacterial antibiotic resistance genes (S.ARG) remained stable in AN but rose with the N addition level in AU (Fig. 4G). Moreover, the minimal doubling time of the bacterial community was shortened during adding N fertilizers (Fig. 4H), but the average 16S rRNA gene copy numbers significantly decreased with N level in AU while not in AN (Fig. 4I). The RFMs indicated that resources and stress were the most important drivers for almost all microbial traits except genome size (Fig. 4K–T). The latter was determined by the number of lysogenic phages, which was also influenced by resources and stress (Figs. 4P and S15). These results demonstrated that the adaptation of the microbiomes to resources and stress could also be embodied by other microbial traits.

In summary, the adaptability of microbiomes driven by shifting resources and stress under AN and AU addition could be embodied by the different patterns of bacterial taxa, bacterial functional potential, and other microbial traits (Figs. 2–4).

### The Y-A-S life history strategies of microbiomes drove their adaptability to resources and stress under N deposition

Procrustes analysis showed the strong relevance between the bacterial community composition and functional potential (*M*^2^ = 0.112, *P* = .001, Fig. S16A; *M*^2^ = 0.127, *P* = .001, Fig. S17). To further clarify the relationship between them, we assembled genomes from metagenomic data. A total of 134 non-redundant, high-quality bacterial MAGs were captured (Figs. S18 and S19). Among them, 79 MAGs were identified in AN, with a higher representation of *Actinomycetota* and *Chloroflexota* compared to AU (Fig. S20A, C). In contrast, 73 MAGs were detected in AU, which exhibited a greater abundance of *Pseudomonadota* and *Bacteroidota* compared to AN (Fig. S20B, D). The functional preference analysis and enrichment analysis found significantly different functional preferences between these dominant bacterial taxa in AN and AU. In detail, the functions of Y and S strategies were mainly preferred by MAGs in AN, while A and S strategies were mainly preferred by MAGs in AU (Fig. S16B, C). This suggested that the enriched bacterial taxa had corresponding Y-A-S strategies for the adaptation to resources and stress under AN and AU treatment.

Besides, Procrustes analysis also found a strong relevance between the bacterial community composition and microbial traits (*M*^2^ = 0.315, *P* = .001; *M*^2^ = 0.332, *P* = .001; Fig. S21). After binning and calculating microbial traits of each MAG (Fig. S19), we calculate microbial traits at community level based on community-weighted means (CWMs) method [76]. The results were almost consistent with the collective features of the whole bacterial community, except S.ARG in AN (Figs. 4 and S22). In detail, the diversity of bacterial functional genes (S.KO) rose with increasing N levels, while the minimal doubling time and GC content decreased with N levels in both AN and AU (Fig. S22A, C, D). The genome size was only enlarged in AN but was minified in AU (Fig. S22E). These results suggested that the enriched bacterial taxa also had corresponding microbial traits for the adaptation to resources and stress.

**Figure 5:**
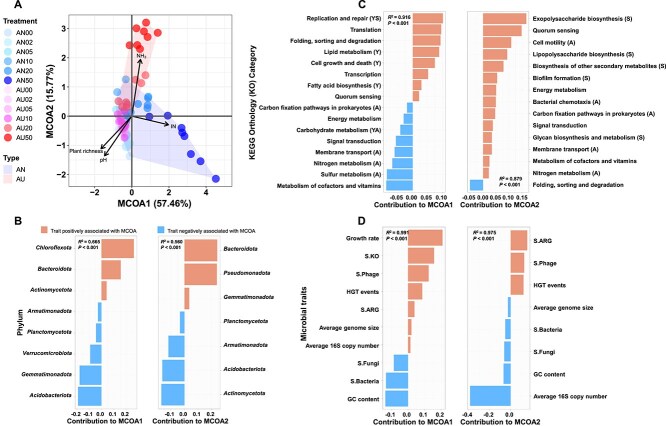
The Y-A-S life history strategies of the microbiomes under AN and AU treatment. (A) Multitable co-inertia analysis (MCOA) with 2D trait space reflected consistent trait associations across soil bacterial community composition, bacterial functional potential, and microbial traits under AN and AU addition. The bacterial community was from 16S rRNA gene amplicon data. Dots represented the microbial communities from the 96 samples used in this study along the two dimensions. Significant resource and stress indexes were presented as vectors on the MCOA plot using the “envfit” (based on 999 permutations) at *P* < .05. (B–D) The contributions of variables to the MCOA dimensions. Only the variables with significant correlation (*P* < .05) with each dimension or significant in the linear regression models were expressed in this figure. The R^2^ values and P-values of *F*-test are shown. ^***^P < .001.

MCOA strongly confirmed the relevance among bacterial community composition, bacterial functional potential, and microbial traits under AN and AU, with MCOA1 and MCOA2 explained more than 73% of the variation (Figs. 5 and S23A). N availability was the most important factor for AN while NH_3_ toxicity for AU (Figs. 5A and S23B, C). Using the bacterial community from metagenomic data for MCOA got almost the same results (Fig. S24). In addition, the enrichment patterns of fungal community were still different between AN and AU addition, and the variation patterns of the fungal community composition were highly similar as that of bacteria (Figs. S25 and S26A, B). Note that there was still consistent result when considering fungal community into MCOA (Fig. S26C–F).

In bacterial community composition, the phyla *Chloroflexota*, *Bacteroidota*, and *Actinomycetota* had the highest contributions to MCOA1, while the phyla *Bacteroidota*, *Pseudomonadota*, and *Gemmatimonadota* were the main contributors to MCOA2 (Figs. 5B, S27, S28). On the aspect of bacterial functional potential, the functions of Y and S strategies had the most contributions to MCOA1 (Figs. 5C and S29), while A and S strategies were the main contributors to MCOA2 (Figs. 5C and S30). It is noteworthy that the microbial traits provided the highest contribution to the MCOA (Fig. S23D). Among them, growth rate was the most important contributor to MCOA1, while S.ARG, S.Phage and HGT events were the most important contributors to MCOA2 (Figs. 5D, S31, S32). These results suggested that *Chloroflexota* and *Actinomycetota* with Y strategy might be favored by more resources under benign environment, while *Pseudomonadota* and *Gemmatimonadota* with A and S strategies might resist higher stress under harsh environment. *Bacteroidota* might also resist stress while taking advantage of abundant resources.

In summary, the adaptability of microbial taxa to changing resources and stress induced by AN and AU addition was governed by their Y-A-S life history strategies, which were reflected in corresponding functional potential and microbial traits.

### The adaptability of microbiomes to resources and stress were mainly accomplished by “move” under N deposition scenarios

**Figure 6:**
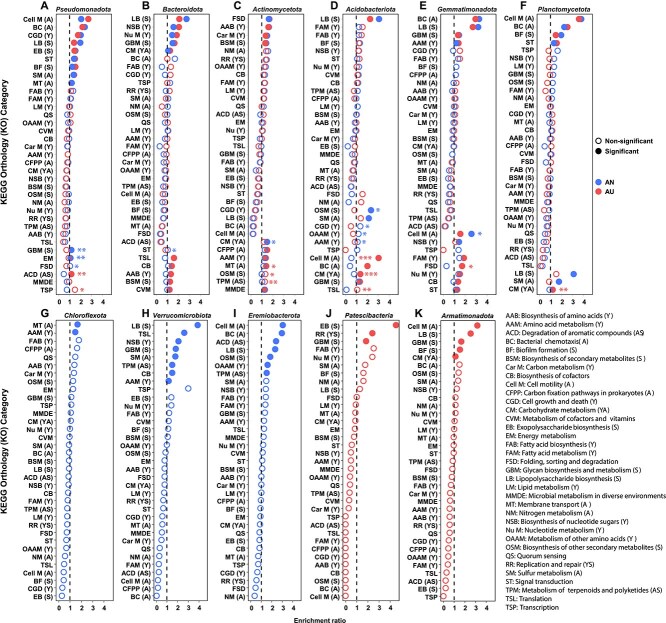
The functional preference showing Y-A-S life history strategies of each bacterial phylum under AN and AU treatment. Functional enrichment analysis showed the preferred KOs in each bacterial phylum. The solid circles indicate the significant enriched categories with a P-value < .05 in a hypergeometric test. The asterisk indicates the significant difference between AN and AU in a hypergeometric test. ^*^, .01 < *P* < .05; ^**^, .001 < *P* < .01; ^***^, *P* < .001.

To evaluate the relative contribution of “move” and “evolve” to microbiome adaptability, we examined bacterial phyla that were present in both AN and AU. Notably, microbial traits of the same phylum did not differ significantly between AN and AU (Fig. S33). Functional preference analysis of MAGs from these shared phyla further revealed consistent Y-A-S life history strategies across treatments (Figs. 6A–F, S34 and S35), suggesting limited evolutionary adaptation at the phylum level. These results imply that the adaptation of microbial community to N-induced changes in resources and stress may be achieved largely by “move”. Based on this deduction, we also analyzed the functional preference of phyla uniquely enriched in either AN or AU to characterize their Y-A-S strategies (Fig. 6G–K).

Consistent with our hypothesis, *Pseudomonadota* and *Gemmatimonadota* were enriched in KOs involved in resource acquisition (A strategy) and stress tolerance (S strategy) (Fig. 6A, E). Their relative abundances were higher under AU than AN and showed positive correlations with MCOA2 (Figs. 2, S6, S7, S28). *Chloroflexota* was enriched in KOs associated with growth (Y strategy) and resource acquisition (A strategy) (Fig. 6G), suggesting better adaptation to resource-rich conditions. This phylum accumulated preferentially in AN and was positively correlated with MCOA1 (Figs. 2, S6, S7, S27). *Actinomycetota* displayed a functional preference for secondary metabolite production (S strategy), while showing reduced engagement in lipopolysaccharide biosynthesis and biofilm formation (S strategy) (Fig. 6C). This functional profile likely enhances competitiveness for resources and niches while remaining susceptible to acidic or NH_3_ stress, which may explain their general enrichment under the relatively low-stress AN treatment. It’s worth noting that *Bacteroidota*, which was positively correlated with both MCOA1 and MCOA2, exhibited Y and S strategies (Fig. 6B), supporting their accumulation in both AN and AU (Figs. 2, S27, and  S28). Interestingly, although *Acidobacteriota*, *Planctomycetota*, and *Armatimonadota* were enriched in functions associated with A or S strategy (Fig. 6D, F, K), their abundances did not increase in AU and were negatively correlated with MCOA2 (Figs. 2 and S28). As AU addition also induced moderate eutrophication, the lack of a Y-strategy preference likely limited their growth advantage compared to *Pseudomonadota*.

**Figure 7:**
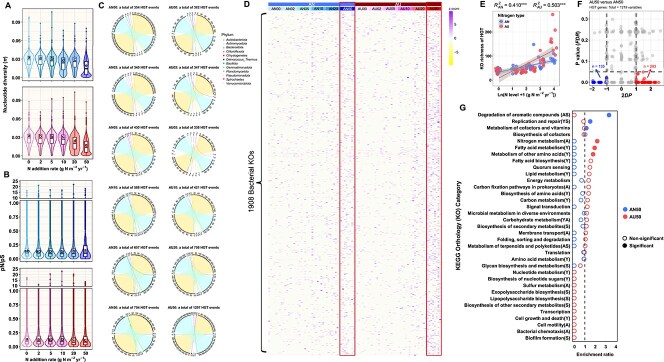
Analysis of evolutionary adaptation under AN and AU addition. (A, B) Violin plots displaying nucleotide diversity (*π*) and pN/pS ratios across N application levels in AN (n = 12 484, 12 577, 16 019, 21 125, 20 077, 14 285 for *π* and n = 12 179, 12 279, 15 419, 20 121, 19 206, 12 830 for pN/pS) and AU (n = 13 184, 12 950, 12 903, 15 774, 26 582, 43 844 for *π* and n = 12 854, 12 521, 12 543, 15 220, 25 052, 40 458 for pN/pS). In each plot, the bold horizontal line denotes the median, the box spans the 25^th^ to 75^th^ percent quartiles, and the whiskers indicate the scope from the highest to lowest data scores. Dots represent outliers. Different letters in the boxes indicate significant differences in *π* or pN/pS among N treatments, determined by Wilcoxon rank-sum test. (C) Chord diagrams representing HGT events among bacterial phyla under AN and AU treatments. The width of every ribbon represents the number of HGT events within or between phyla. The chord diagrams were colored by bacterial phylum. (D) The frequency of horizontally transferred genes, annotated by KO (n = 1908) and standardized as *Z*-score. The red rectangles indicate the higher frequency of horizontally transferred genes in samples of AN50 and AU50. (E) The richness of horizontally transferred genes under AN and AU addition. (F) Preference analysis of horizontally transferred genes in AN50 and AU50. In the 2D preference (2*DP*) plot, KOs significantly preferred in AN50 (*P_FDR_* < .05 and 2*DP* < −1.0) are in the lower-left quadrant, while those preferred in AU50 (*P_FDR_* < .05 and 2*DP* > 1.0) are shown in the lower-right quadrant. (G) Functional enrichment analysis of transferred KOs preferred under AN50 and AU50. The solid circles indicate significantly enriched categories with a *P*-value < .05 in a hypergeometric test.

To assess the contributions of “evolve” to the microbial adaptability, we first analyzed the evolutionary ecology of functional genes by profiling intra-population genetic diversity (microdiversity) of microbial communities under each N treatment (Fig. 7A, B). The results revealed consistently low nucleotide diversity (*π*; average 0.015–0.028), which declined with increasing N input under both AN and AU (Fig. 7A). In addition, pN/pS ratios remained low across all N levels (average 0.17–0.20, with 87.6%–92.5% of ratios less than 0.4; Fig. 7B). These results suggest strong purifying selection and limited mutation accumulation under N addition. We further examined HGT events in the microbial community (Fig. 7C–G). In both AN and AU treatments, the majority of HGT events occurred within *Actinomycetota* (193–588 HGTs per treatment) and *Pseudomonadota* (118–602 HGTs per treatment) (Fig. 7C). The frequency and diversity of 1908 horizontally transferred KOs were increased with N addition level (Fig. 7D, E). Notably, the functional preferences of these transferred KOs differed between AN and AU (Fig. 7F). Specifically, in AN50, where the bacterial community exhibited a Y-S preference, transferred KOs were notably enriched in degradation of aromatic compounds (A and S strategies), and additionally in replication and repair (Y and S strategies) and metabolism of cofactors and vitamins (Fig. 7G). In contrast, under AU50, characterized by an A-S-preferring community, transferred KOs were enriched in N metabolism (A strategy), fatty acid metabolism (Y strategy), and metabolism of other amino acids (Y strategy) (Fig. 7G). These results suggest that HGT expanded the functional repertoire of the community in a complementary manner, rather than fundamentally shaping its core strategic preferences under both AN and AU (Figs. 3 and Fig. 7G).

**Figure 8:**
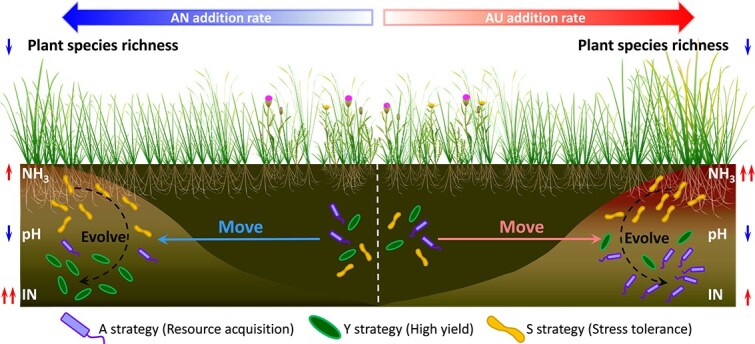
The conceptual illustration showing how soil microbes adapted to changing resources and stress under AN and AU treatment. Consistent with our hypothesis, Y-strategy bacteria were mainly enriched under AN treatment, which provided more abundant resources and lower stress than AU treatment. In contrast, A- and S- strategy bacteria were favored under AU addition, characterized by fewer resources and stronger stress. The adaptability of soil microbiomes to these resource-stress shifts was mainly accomplished by niche conservatism (“move”) in a 6-year simulated N deposition experiment. NH_3_, ammonia toxicity caused by ammonia volatilization after N fertilization; IN, soil available N. The number of bacterial symbols indicates their relative abundance.

In summary, although “evolve” contributed partially to the adaptation of microbiomes, the prevalence of strong purifying selection in the microbial community constrained the accumulation of adaptive mutations. Moreover, the functions acquired by HGT were more complementary than essential. Thus, the adaptability of microbiomes to resources and stress under N deposition was mainly accomplished by “move” (Fig. 8).

## Discussion

Our N addition experiment, which created a spectrum of soil physicochemical conditions, reveals the adaptation mechanisms of soil microbiomes under N deposition scenarios, thereby informing and inspiring future research on microbial adaptability to resource and stress changes.

As expected, microbial traits effectively reflected the adaptive features of microbiomes to resource and stress shifts under N deposition, exhibiting the highest contribution in the MCOA (Fig. S23D). The taxonomic diversity and number of phages increased with elevated N addition (Figs. 4C and S15A), inferring that phages may serve as mediators of HGT events [77]. In addition, while previous studies have indicated that microbial communities with higher functional diversity tend to have larger genome size [78, 79], in our study, a significant increase in average genome size was observed only in AN (Figs. 4F and S22E). This pattern may be attributed to the fact that maintaining larger genomes requires greater quantities of N and phosphorus for cell division [80, 81], conditions that are more favorably met in AN. Besides, the higher proportion of lysogenic phages in AN compared to AU (Fig. S15B, C) may further promote genome expansion [82]. Thus, the increase in functional diversity in AU was probably mainly driven by enhanced HGT events, while that in AN (Fig. 4E) likely resulted from a combination of both HGT and enlarged average genome size. It is generally recognized that multiple rRNA operon copies in prokaryotes are linked to rapid reproduction [83], and that abundant resources select fast-growing bacteria [84]. But in this study, we observed a decoupling between growth rates of bacterial communities and average 16S rRNA gene copy numbers, particularly in the more stressed AU treatment (Fig. 4H, I). This suggests that despite resource availability, increasing stress might force the bacterial community to trade maximum growth potential for stress tolerance by curtailing the 16S rRNA gene copy number [85]. Consequently, microbial growth rates may decline as environmental stress intensifies.

Although AN and AU additions recruited distinct microbial taxa through different resource and stress regimes, the same bacterial phyla under both treatments showed almost no differences in either functional potentials or microbial traits (Figs. 6 and S33). HGT, a major driver of prokaryotic evolution, is known to enhance genetic diversity and facilitate environmental adaptation of bacteria [86–88]. However, the transferred genes in this system appeared to serve more complementary than essential roles within the existing functional inventory (Figs. 3E and 7G). This suggests that while environmental changes under N deposition promote HGT between microbes, the acquired genes may initially contribute to functional diversification rather than conferring direct and specific adaptation to environmental stressors [86]. Furthermore, the decreased *π* and consistently low pN/pS ratios indicated strong purifying selection within the microbial community (Fig. 7A, B), which likely restricted the accumulation of adaptive mutations under N treatments [89, 90]. Although 6 years is a substantial period for microbial evolution [91], the stochastic nature of the process may considerably delay the emergence of advantageous mutations [87, 88, 92]. Therefore, we conclude that microbial adaptation to the 6-year N deposition was mainly accomplished by “move” (Fig. 8). This finding also provides evidence that inferring microbial traits from taxonomic data based on literature or databases is credible over short evolutionary timescales.

Our study also has some limitations. Firstly, although the inborn Y-A-S life history strategies largely explain the consistent distribution patterns of bacterial taxa and functional potential under AN and AU treatments, they should not be equated with actual competitive fitness. Functional potential annotated as Y, A, or S strategies may not fully capture realized ecological behavior, as illustrated by *Verrucomicrobiota*. Although this phylum possesses functional traits associated with Y and S strategies (Fig. 6H), it was depleted under both AN and AU conditions (Figs. S6 and S7), suggesting that its actual competitive capacity may be constrained by other taxa [93]. Secondly, in our study, the assignment of functional categories to Y-A-S strategies was mainly relied on published annotations. Further experimental and genomic validation is needed to fully resolve the life-history characteristics of many bacterial lineages. Thirdly, although the functional preferences were almost consistent within phyla across AN and AU, there were still deviations that could not be explained by HGT, particularly in *Acidobacteriota*. On the one hand, there might be influence of other evolutionary processes such as random drift, point mutation, and homologous recombination [94]. On the other hand, the trait-based functional profiles might not be fully conserved at the phylum level. Finally, MCOA revealed consistent patterns of variation among fungal communities, bacterial communities, bacterial functional potential, and microbial traits under AN and AU treatments. The analysis further confirmed that N availability served as the most important driver of community variation under AN, while NH_3_ toxicity was the dominant factor under AU (Fig. S26). Nevertheless, whether fungal communities employ adaptive strategies similar to the Y-A-S framework observed in bacterial communities remains an open question, due to the inherent limitations of metagenomic sequencing in characterizing the functional potential of fungal communities. This represents an important direction for future research.

## Conclusions

In conclusion, the different resource and stress regimes induced by AN and AU addition drove distinct microbiome assembly patterns, which were fundamentally shaped by the inherent Y-A-S life history strategies of the constituent taxa. Our findings demonstrate that soil microbes may prioritize moving to track suitable niches over relying on evolutionary adaptation in response to N deposition. This supports the reliability of inferring microbial functional traits from taxonomy data using established literature or database annotations in the context of near-term environmental change. Furthermore, our results suggest that grassland soil microbiomes may exhibit resilience to short-term N deposition and other anthropogenic disturbances, provided that appropriate management practices are implemented to restore and maintain soil environments with balanced resource availability and minimal stress.

## Supplementary Material

Supplementary_Information-20251113_ycaf215

Supplementary_Table_4_KOs_and_Y-A-S_category_in_this_study_ycaf215

## Data Availability

All metabarcoding and metagenomics sequences in this manuscript are deposited in NCBI database PRJNA1211281 (https://www.ncbi.nlm.nih.gov/sra/PRJNA1211281).

## Code availability

The source code to accomplish the analyses in this manuscript is available on GitHub (https://github.com/yuqyfisher/YAS_N_microbiome).

## Additional information

**Correspondence and requests for materials** should be addressed to Ying Huang.
