# Supplementary material for: The adaptability of grassland soil microbiomes to resource and stress shifts is mainly accomplished by niche conservatism under nitrogen deposition
Source: ISME Commun. 2025 Nov 20;5(1):ycaf215. doi: 10.1093/ismeco/ycaf215 (PMC12704437; doi:10.1093/ismeco/ycaf215)
Supplement: Supplementary_Information-20251113_ycaf215 [file supplementary_information-20251113_ycaf215.pdf]

Supplementary Information

for

“The adaptability of grassland soil microbiomes to resource and stress shifts is mainly accomplished by niche conservatism under nitrogen deposition” by Yu et al.

Supplementary Text

Materials and Methods

The study site and experimental design

The soil in this study site is classified as Chernozem by the Chinese Soil Classification System with a pH of 6.8-7.0 (0-10 cm). The dominant herbaceous plants are *Leymus chinensis* and *Stipa baicalensis.*

The N addition experiment was started in 2014 using a completely randomized block design. N fertilizers were uniformly applied when the grassland turned green every year and continued for 6 years (2014-2020). There are eight blocks, each containing 60 plots (10 × 10 m^2) arranged in a full factorial combination of five N types (AN: ammonium nitrate addition, AU: urea addition, AR: slow-release urea addition, AC: ammonium bicarbonate addition and AS: ammonium sulfate addition), six application levels (0, 2, 5, 10, 20, 50 g N m^{-2} yr^{-1}), and two mowing types (mown and unmown). More details on the study site and experimental design can refer to Yang et al (2022 & 2023) [1, 2].

DNA extraction and Sequencing

After DNA extraction, the concentration of each DNA sample was determined using Qubit 2.0 Flurometer (Life Technologies, CA, USA). And the quality of DNA was checked using electrophoresis in 1% agarose gels.

Raw sequencing reads from metagenome shotgun sequencing were processed to obtain quality-filtered reads for further analysis. Briefly, sequencing adapters were removed from sequencing reads and low-quality reads were trimmed using a sliding-window algorithm.

The amplification for bacteria and fungi was carried out in a 25 µL reaction solution, which contained 4.5 µL ddH_2O, 12.5 µL 2 × PCR Buffer, 5 µL 2 mM dNTPs, 0.5 µL KOD-FX-Neo polymerase (1.0 U μL^{-1}, Toyobo Inc, Osaka, Japa), 0.75 µM of each primer and 1 µL of template DNA. The PCR conditions for bacteria were set as 95 °C for 3 min, followed by 35 cycles of denaturation at 95 °C for 30 s, annealing at 56 °C (Bacteria) or 58 °C (Fungi) for 30 s, and extension at 68 °C for 40 s, then final extension at 68 °C for 10 min. DNA2000 marker was the positive controls. Meanwhile, we used sterile ultrapure water as negative controls to test the presence of contamination in reagents and there were no bands observed. The PCR products were purified using paramagnetic bead technology (Agencourt AMPure XP, USA) and 50 ng purified DNA of each of the 96 samples was pooled and adjusted to 10 ng μL^{-1}.

Metabarcoding analyses

The reads of 16S and ITS rRNA gene sequences were assigned to each sample according to the unique barcode at the 5’ end of 806R and ITS4F, respectively. The quality control was performed using QIIME2 pipeline [3]. After merging the qualified forward and reverse reads, removing the primers, and extracting ITS2 region using ITSx [4], we detected and removed duplicated sequences and all singletons from 16S and ITS

sequences respectively using USEARCH pipeline [5]. The remained sequences were clustered into different operational taxonomic units (OTUs) at a 97% similarity level based on the UPARSE pipeline using the USEARCH (v8.0) and discarded chimeras using MOTHUR (v1.31.2) [6].

Metagenome analyses

To focus on bacteria, the contigs of potential eukaryotic contigs were detected using EukRep (v0.6.7) [7] . And the potential viral contigs were detected using Virsorter2 (v2.2.3) [8]. Then the protein-coding genes of all contigs and viral contigs were predicted by Prodigal (v2.6.3) [9]. As we found that the gene numbers of phage in each sample were less than 1% of the total prokaryotic genes (Fig. S4) and the numbers of eukaryotic contigs in most samples (95 of the 96 samples) were less than 1% of the total contigs (Fig. S5), we considered the detected protein-coding genes mainly represented the functional potential and functional gene diversity (S.KO) of the bacterial community.

The bacterial 16S rRNA genes were extracted from metagenomic data using BBMap (v39.01) [10]. Then we clustered the non-duplicated sequences into OTUs and did taxonomic identification using the same step as Metabarcoding analyses. The non-bacteria sequences only amounted to 1.39% of total sequences.

The sequences of virome were detected using Virsorter2 (v2.2.3). The integrase genes were annotated using Prodigal and PfamScan pipeline [11]. Each phage genome with integrase gene was consider as a lysogenic phage [12]. And phage diversity was determined from the metagenome sequence by Kaiju [13].

Statistical analyses

The diversities of the bacteria (S.Bacteria), fungi (S.Fungi), phage (S.Phage), bacterial antibiotic resistance genes (S.ARG), and prokaryotic functional gene (S.KO) were calculated based on Shannon's index using "*vegan*" package [14]. We did NMDS analysis with Bray-Curtis dissimilarity for bacterial community and for bacterial functional potential using the "*vegan*" package, with permutational multivariate analysis of variance (PERM ANOVA) using the adonis command based on 999 permutations. All linear regression models were fitted using two-sided tests, and adjusted R-squared were reported to evaluate the model fit and the strength of the relationships between parameters.

After detecting the HGT events and transferred genes, we constructed chord diagrams to visualize the HGTs between bacterial taxa at the phylum level in the "*circlize*" package [15]. Preference analysis was also used to find the preference of horizontally transferred genes between AN50 and AU50.

Supplementary Figures and Tables

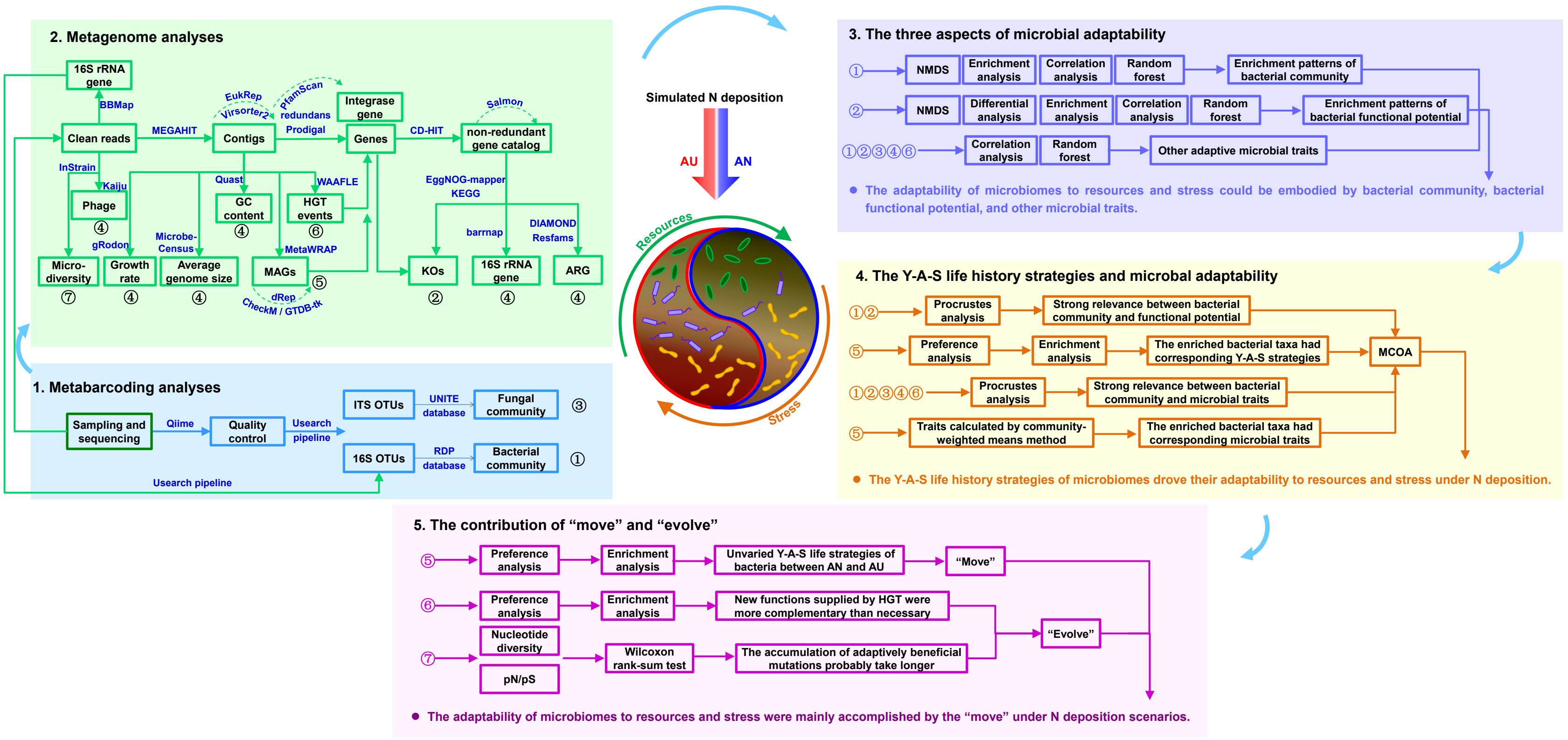

Figure S1. Workflow schematic for exploring the adaptability of grassland soil microbiomes to resources and stress under nitrogen deposition scenarios.

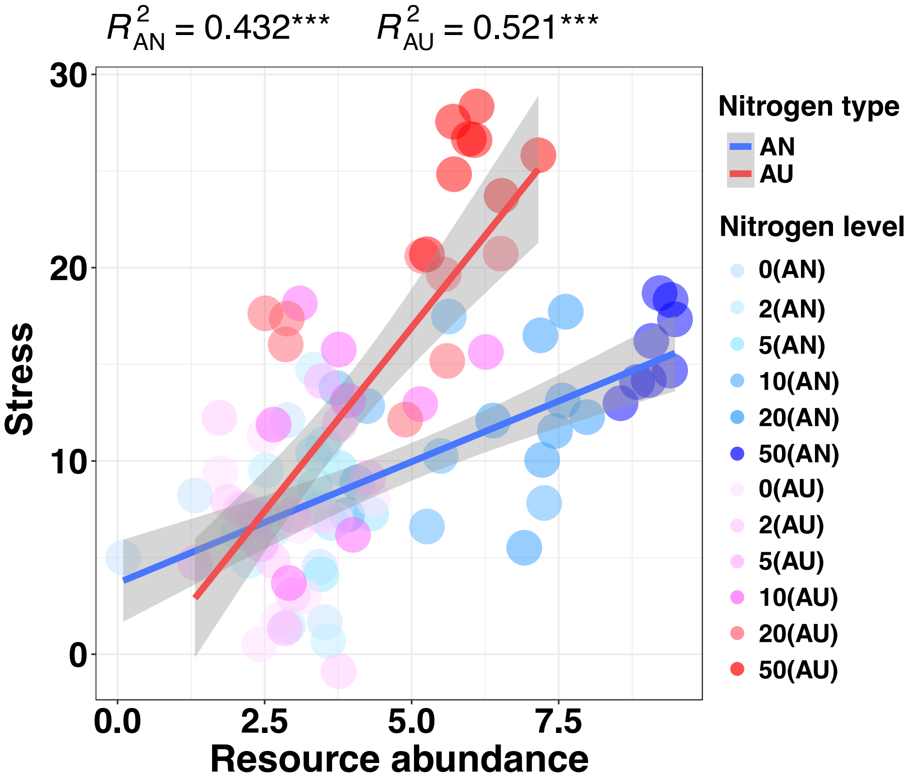

Figure S2. Ratios of stress to resources under the addition of AN and AU.

Resource abundance was measured by soil available N. The stress was calculated as: stress = NH_3 toxicity + |7 - pH| + |21 - plant diversity|. Loss of plant species diversity probably reduces habitat availability for soil microorganisms and was thus considered a biotic stressor. The value 7 represents neutral pH, and 21 corresponds to the maximum plant species richness observed across the plots selected in this study. ***, $P < 0.001$

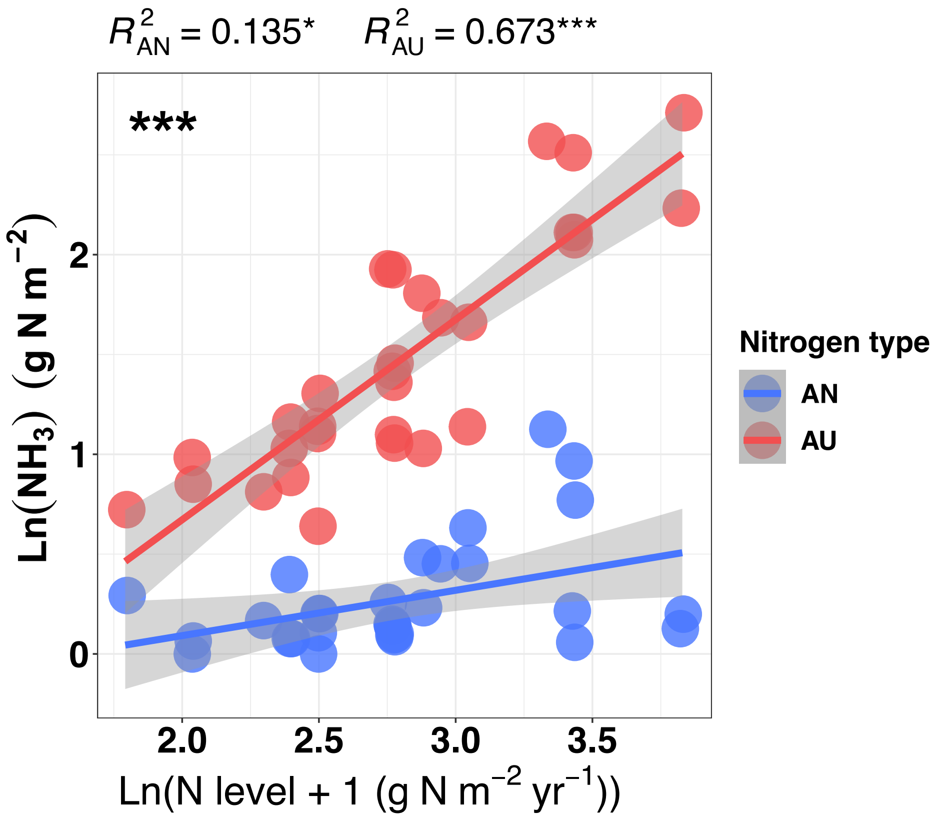

Figure S3. Results of the meta-analysis assessing NH_3 volatilization (as a proxy for toxicity) under the addition of AN and AU.

Data were derived from studies compiled in Table S1. The asterisk in the top-left corner indicates a significant difference between the regression slopes of AN and AU. ***, $P < 0.001$.

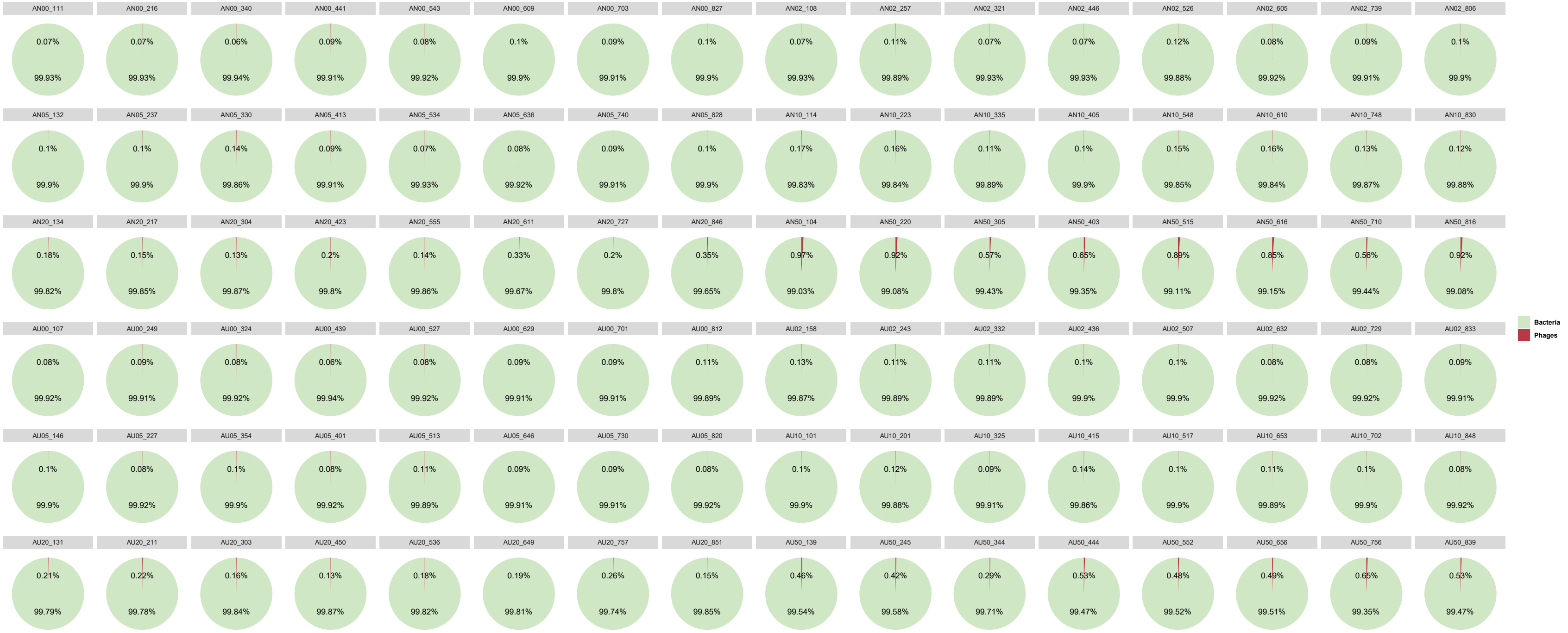

Figure S4. The number of phage genes in each sample, accounting for less than 1% of the total prokaryotic genes.

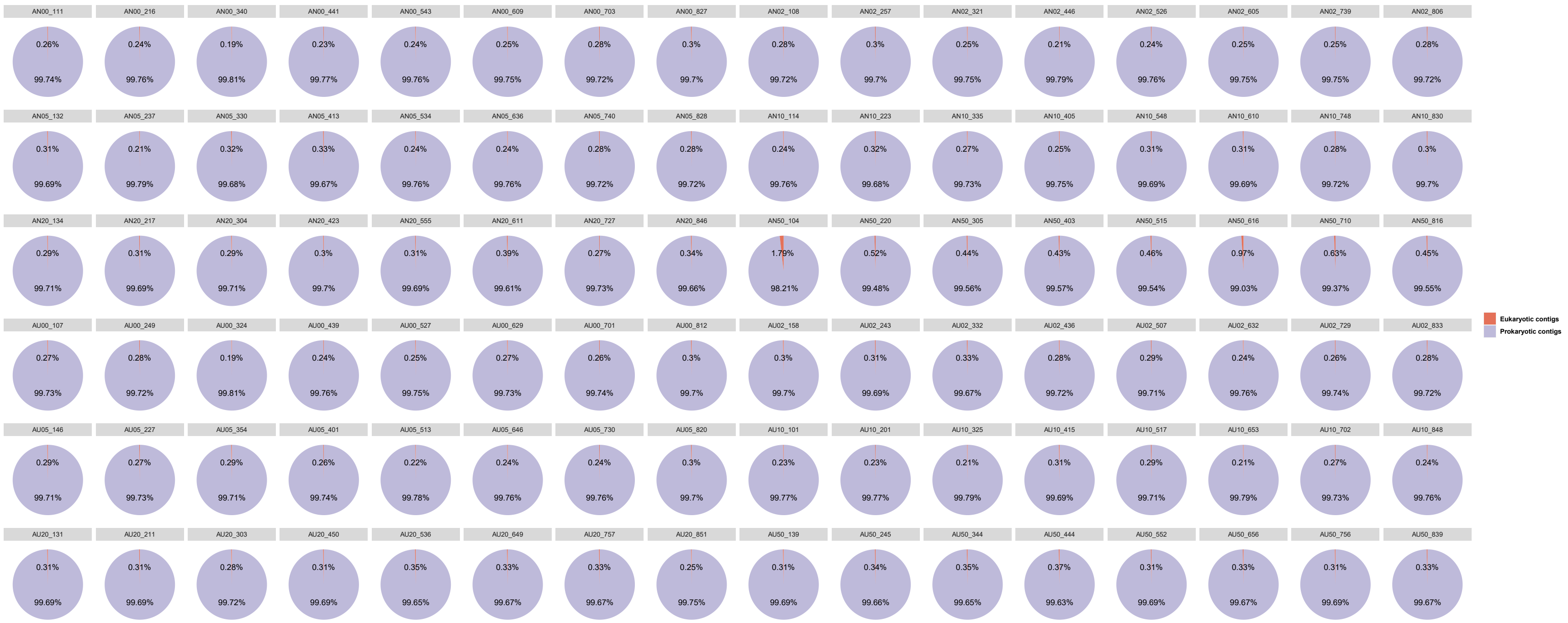

Figure S5. The number of eukaryotic contigs in each sample.

The number eukaryotic contigs accounted for less than 1% of the total contigs in 95 samples and for 1.79% only in sample AN50_104.

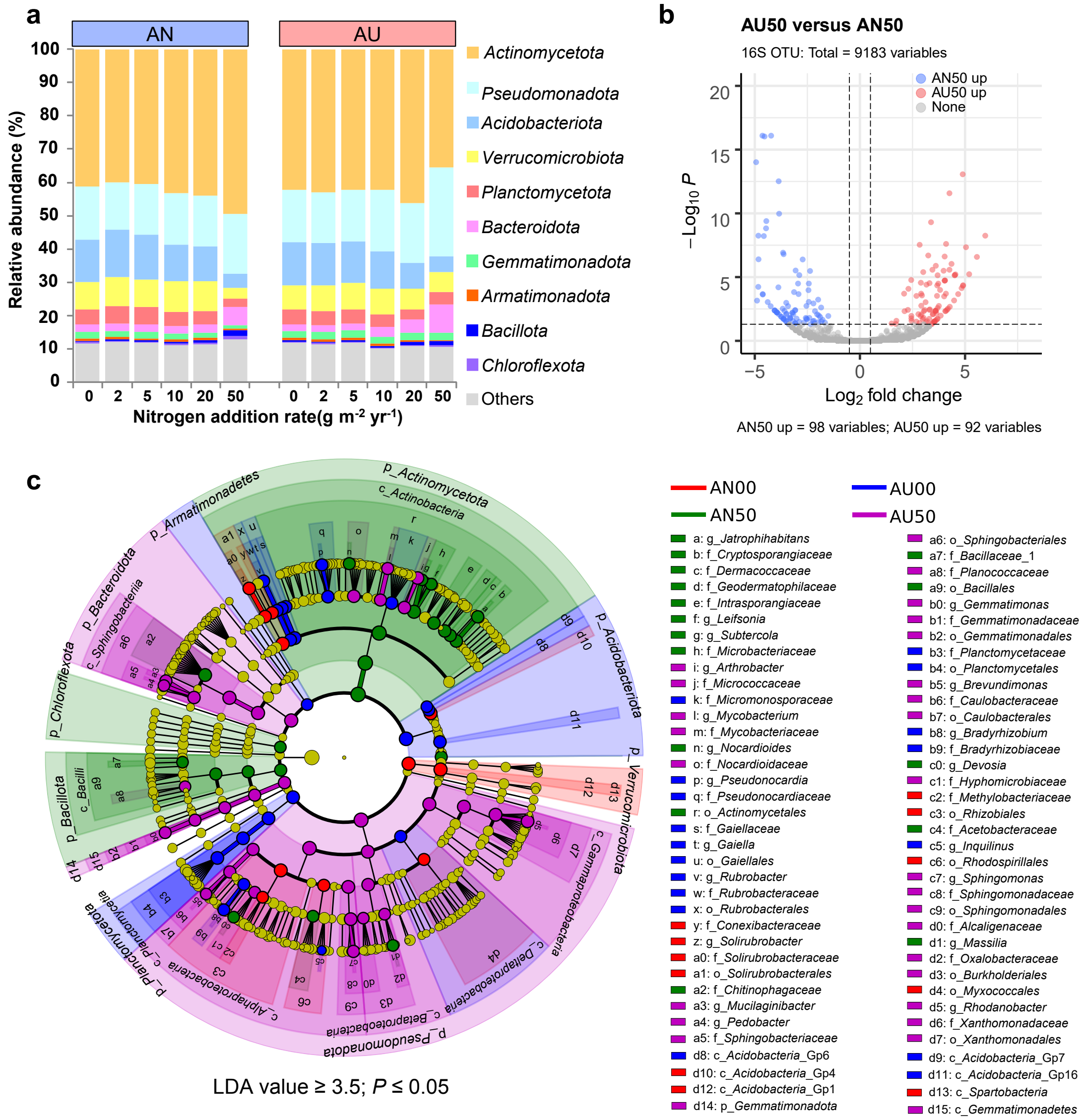

Figure S6. Differences of bacterial community composition between AN50 and AU50 based on 16S rRNA gene amplicon data.

a Bacteria phylum composition along N addition gradients under AN and AU. **b** Volcano plot showed the different enrichment of 16S OTUs from AN50 and AU50 treatment (|LogFC| > 0.5, *P* < 0.05). Differential analysis with two-sided test and P_{FDR} were used for the statistical analysis. **c** LDA effect size (LEfSe) cladogram indicating the phylogenetic distribution of bacterial lineages and LDA scores of enriched bacterial taxa in AN00, AN50, AU00 and AU50. The yellow nodes represent the bacterial taxa with no significant differences, while nodes and sectors with other colors represent significantly enriched

bacterial taxa with an LDA value larger than 3.5. The taxa of *Actinomycetota*, *Chloroflexota* and *Bacillota* were mainly enriched in AN50, while the taxa of *Pseudomonadota*, *Bacteroidata* and *Gemmatimonadota* were mainly enriched in AU50. These analyses were based on 16S rRNA gene amplicon data.

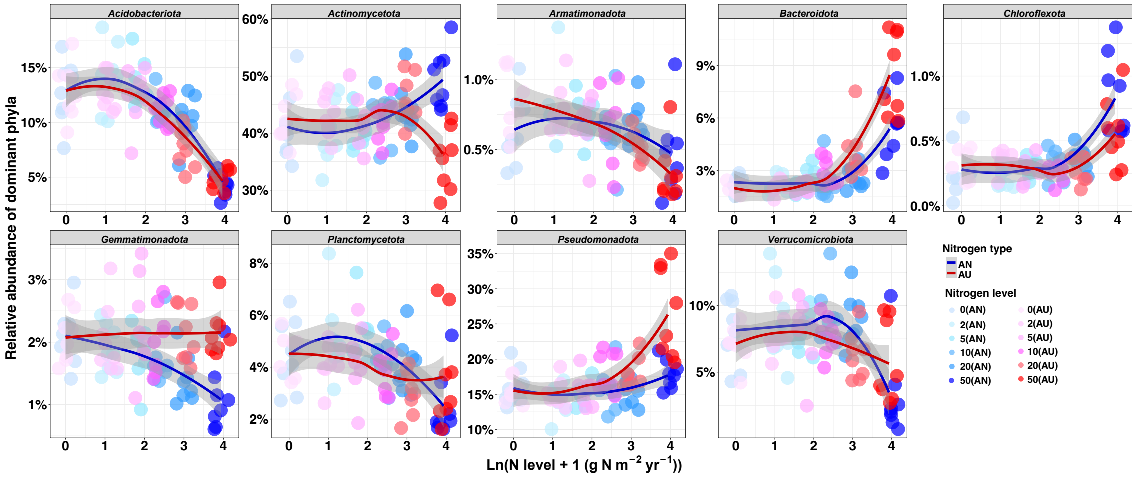

Figure S7. Regression curves depicted the relative abundance of bacterial dominant phyla along N addition gradient under AN and AU treatments based on 16S rRNA gene amplicon data.

Regression curves of the dominant phyla of bacterial community along N levels under AN and AU. Linear regression model with two-side and adjusted R-squared was used for the statistical analysis.

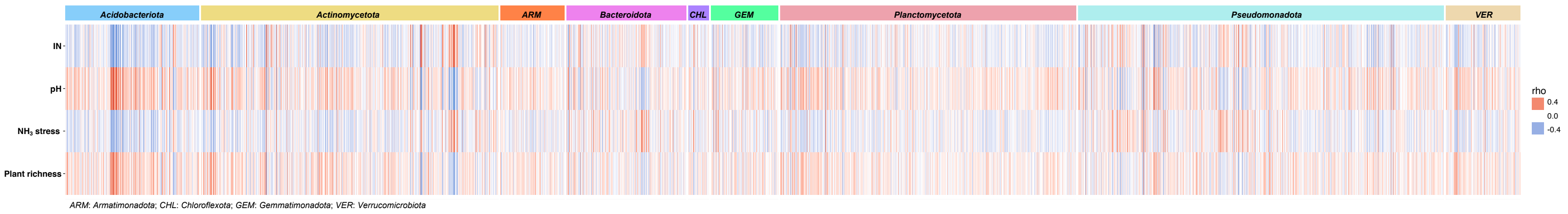

Figure S8. Association between environmental variables and dominant bacterial phyla from 16S rRNA gene amplicon data (*n* = 6075 OTUs).

In general, *Acidobacteriota*, *Armatinondata*, *Gemmatimonadota*, *Planctomycetota* and *Verrucomiceobiota* were positively correlated with soil pH and plant species richness but negatively correlated with soil available N and NH_3 stress. Many OTUs of *Actinomycetota*, *Bacteriodota*, *Chloroflexota* and *Pseudomonadota* were positively correlated with soil available N and NH_3 stress but negatively correlated with soil pH and plant species richness. IN: soil available N.

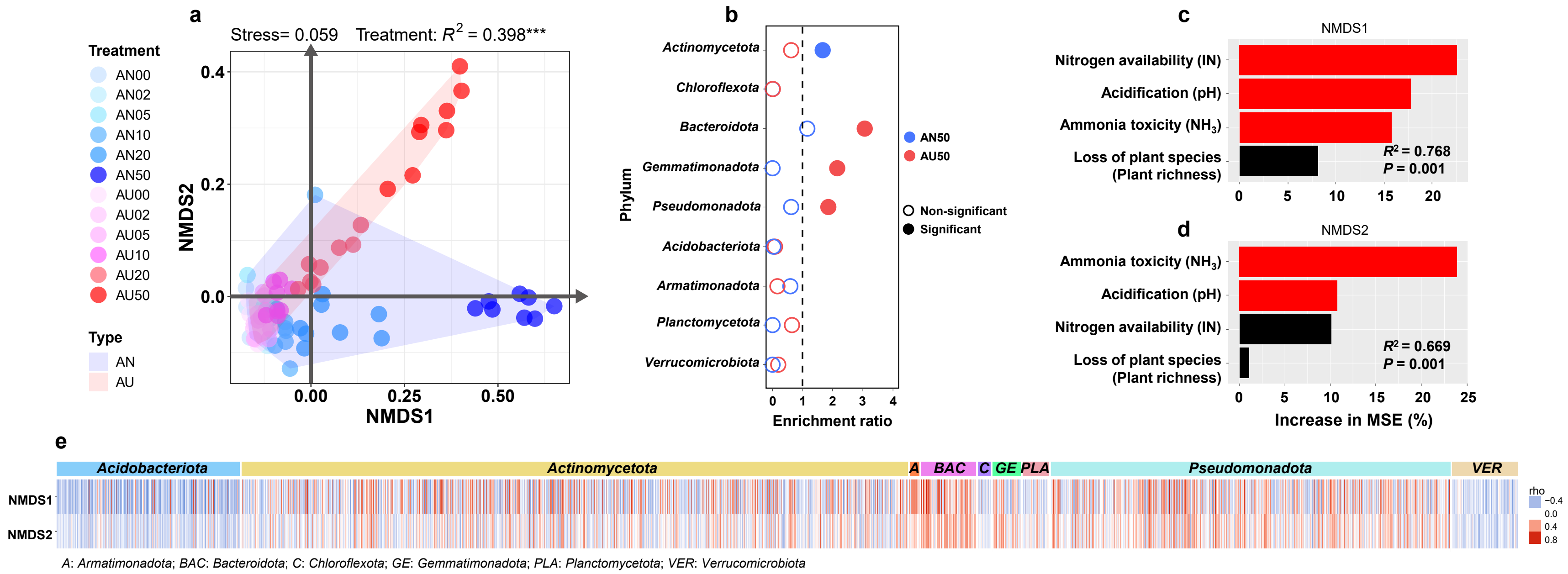

Figure S9. Community patterns of soil bacteria under AN and AU treatment from metagenomic data.

a NMDS analysis of bacterial community with Bray-Curtis dissimilarity and PERM ANOVA showed significant difference between AN and AU. **b** The enrichment analysis of bacterial community at the phylum level. The solid circles indicate the categories for which the enrichment has a P-value < 0.05 in a hypergeometric test. **c-d** The importance of resources and stress represented as the percentage of increase of mean square error (increase in %MSE) with **c** NMDS dimension1 and **d** dimension2 predicted by random forest models. Red bars indicate factors that significantly predict NMDS dimensions and black bar indicate non-significant predicting factors. **e** Association between NMDS dimension and dominant bacterial phyla (n = 10241 OTUs).

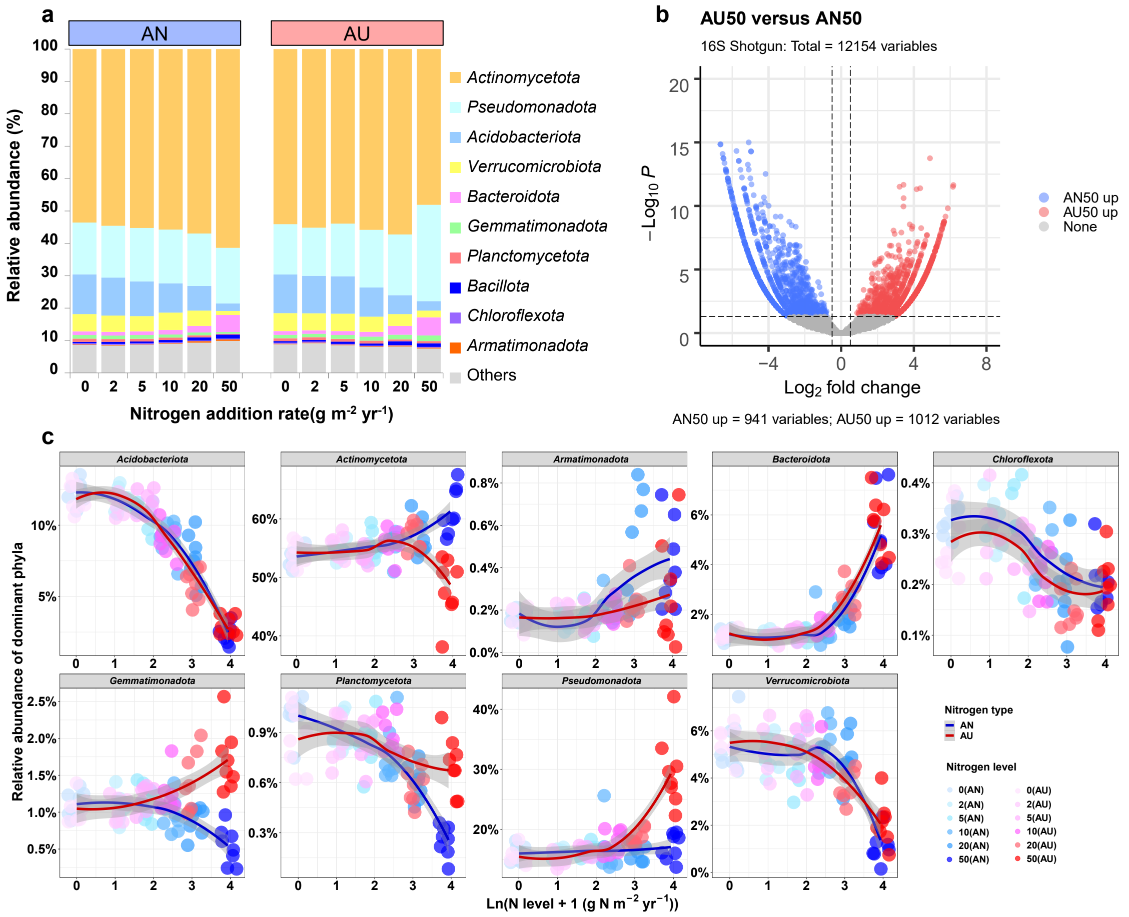

Figure S10. Differences of bacterial community composition from metagenomic data.

a Bacteria phylum composition along N addition gradients under AN and AU. **b** Volcano plot showed the different enrichment of 16S OTUs from AN50 and AU50 treatment (|LogFC| > 0.5, P < 0.05). Differential analysis with two-sided test and P_{FDR} were used for the statistical analysis. **c** Regression curves depicted the relative abundance of bacterial dominant phyla along N levels under AN and AU. Linear regression model with two-side and adjusted R-squared was used for the statistical analysis.

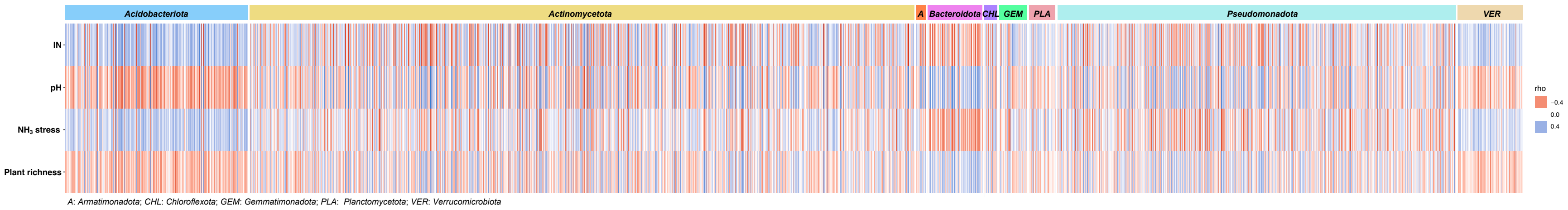

Figure S11. Association between environmental variables and bacterial dominant phyla from metagenomic data (*n* = 10241 OTUs). In general, *Acidobacteriota*, *Armatinondata*, *Planctomycetota* and *Verrucomiceobiota* were positively correlated with soil pH and plant species richness but negatively correlated with soil available N and NH_3 stress. Many OTUs of *Bacteriodota* and *Pseudomonadota* were positively correlated with soil available N and NH_3 stress but negatively correlated with soil pH and plant species richness. Note that the result from metagenomic data was almost the same as that from 16S rRNA gene amplicon data.

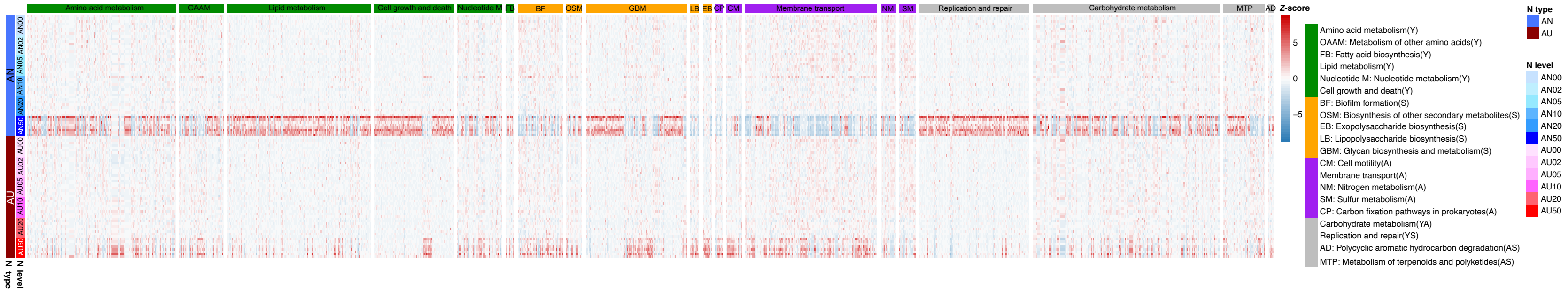

Figure S12. Heatmap showing the relative abundance of KOs classified into Y-A-S strategies under each N treatment.

In general, the functions classified as Y strategy, including amino acid metabolism, metabolism of other amino acids, nucleotide metabolism, lipid metabolism, cell growth and death, fatty acid biosynthesis, replication and repair and carbohydrate metabolism, were more abundant in AN50. The functions classified as S strategy, including biosynthesis of other secondary metabolites, glycan biosynthesis and metabolism and replication and repair, were more abundant in AN50. However, the other functions of S strategy, including biofilm formation, lipopolysaccharide biosynthesis and exopolysaccharide biosynthesis, were more abundant in AU50. The functions classified as A strategy, including cell motility, membrane transport, nitrogen metabolism and sulfur metabolism, were more abundant in AU50.

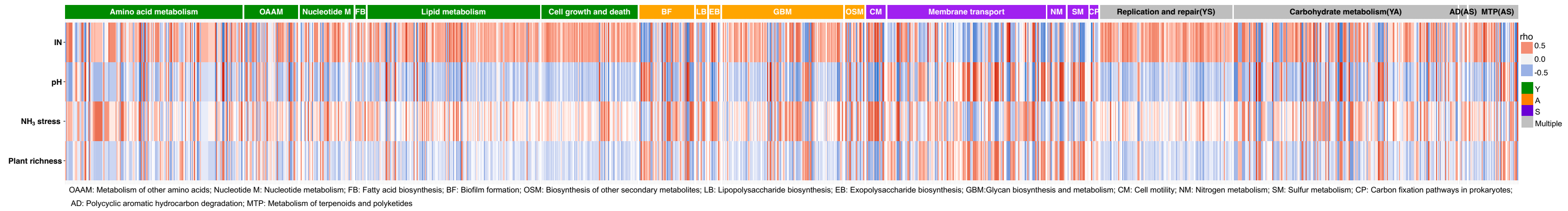

Figure S13. Association between environmental variables and KOs classified into Y-A-S strategies from metagenomic data (*n* = 1405 KOs).

In general, the KOs classified as Y strategy, including amino acid metabolism, metabolism of other amino acids, nucleotide metabolism, fatty acid biosynthesis, carbohydrate metabolism, lipid metabolism and replication and repair, were positively correlated with soil available N and NH_3 stress but negatively correlated with soil pH and plant diversity. The KOs classified as S strategy, including biofilm formation, lipopolysaccharide biosynthesis, exopolysaccharide biosynthesis, glycan biosynthesis and metabolism, biosynthesis of other secondary metabolites and replication and repair, were also positively correlated with soil available N and NH_3 stress but negatively correlated with soil pH and plant diversity, while the KOs of biofilm formation were mainly negatively correlated with soil available N and NH_3 stress but positively correlated with soil pH and plant diversity. The KOs classified as A strategy, including membrane transport, nitrogen metabolism and sulfur metabolism, were also mainly negatively correlated with soil available N and NH_3 stress but positively correlated with soil pH and plant diversity, while the KOs of cell motility were mainly positively correlated with soil available N and NH_3 stress but negatively correlated with soil pH and plant diversity.

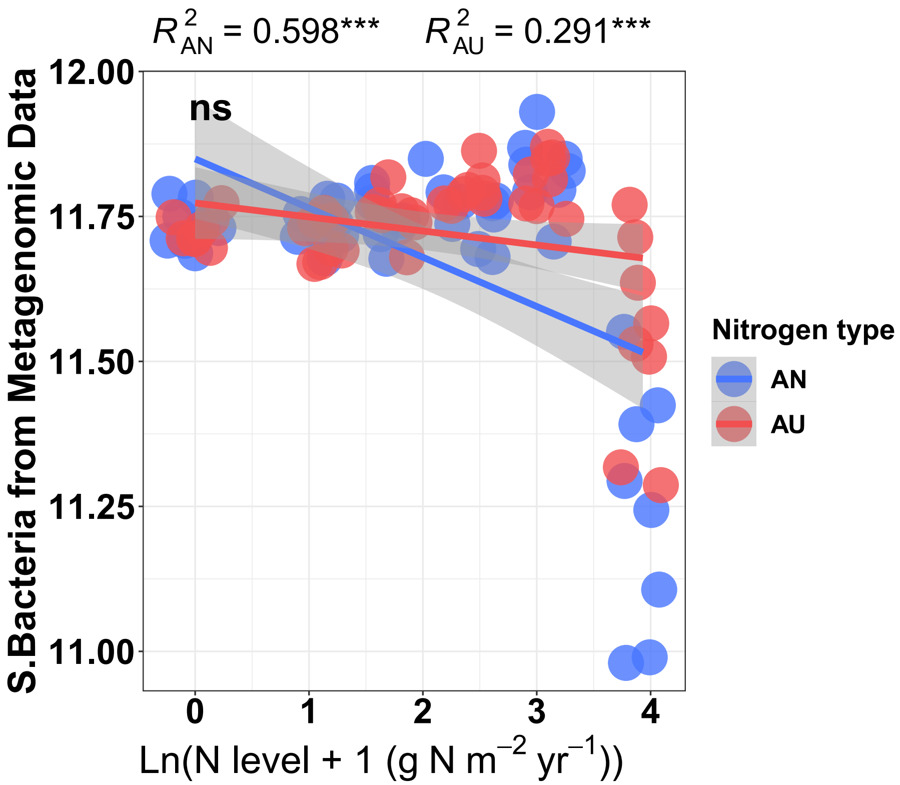

Figure S14. Taxonomy diversity of bacteria in relation to nitrogen addition from metagenomic data.

Taxonomy diversity of bacteria measured by Shannon's index decreased with increasing levels of N addition, with a greater extent at AN than AU. The mark on the top left shows there is no significant difference between the slope of lines AN and AU. ***, $P < 0.001$.

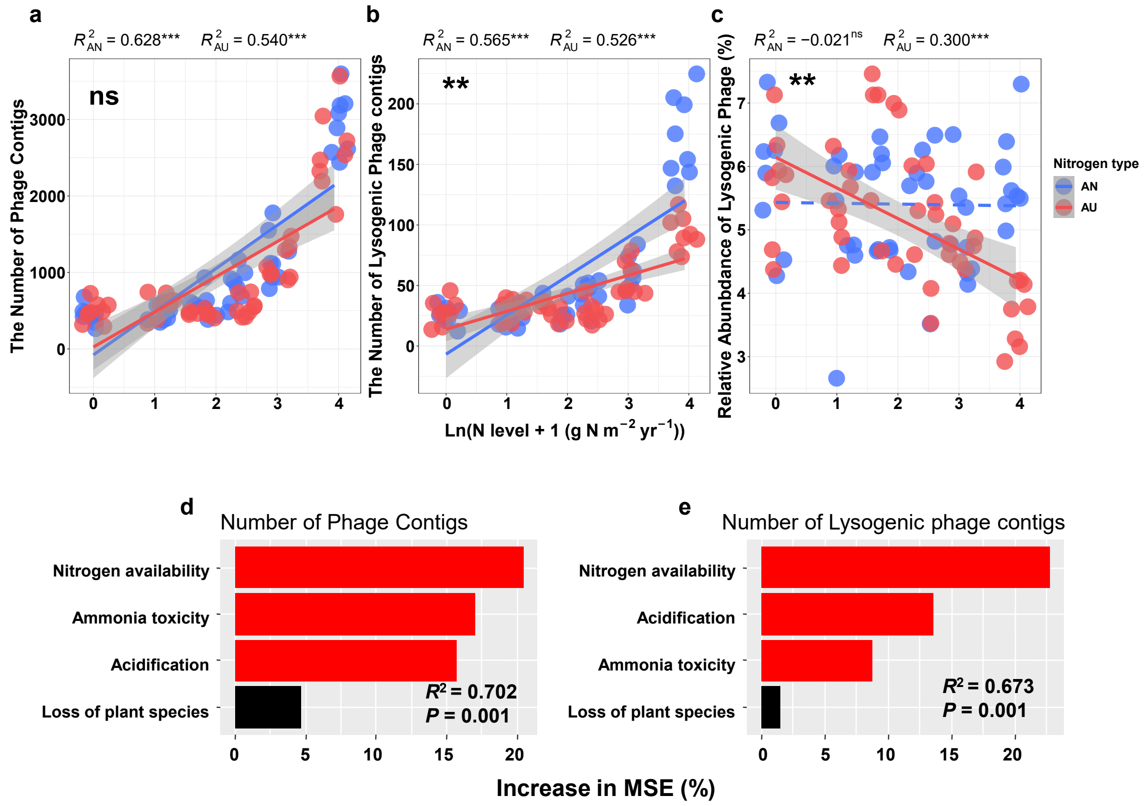

Figure S15. Traits of phages under AN and AU addition.

a-b The blue and red solid lines indicate the significant relationships between factors and AN or AU, respectively, while the dash lines indicate non-significant results. The grey area around the smooth line is the 95% confidence interval. The marks on the top left show whether there are significant differences between the slope of line AN and line AU. **d-e** The importance of resources and stress represented as the percentage of increase in %MSE (increase of mean square error) with traits of phages predicted by random forest models. Red bars indicate factors that significantly predict traits of phages and black bar indicate non-significant predicting factors. ***, $P < 0.001$.

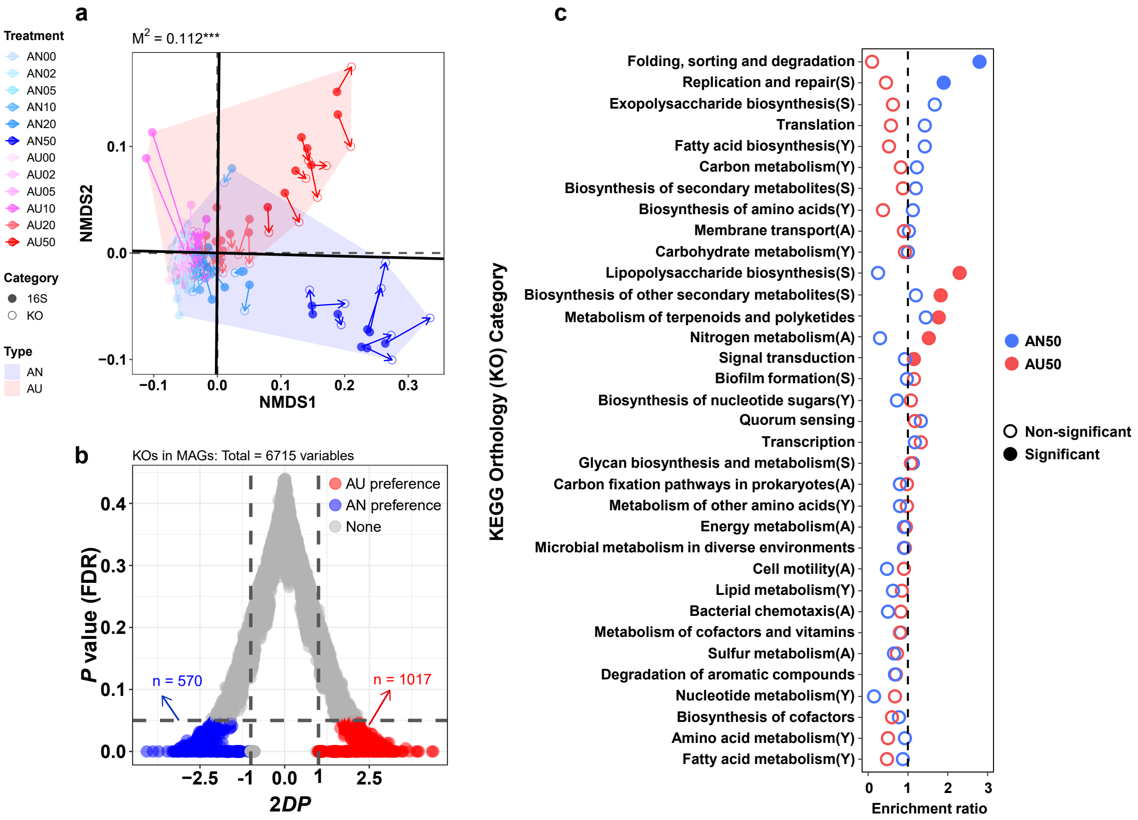

Figure S16. Distribution patterns of bacterial taxa and functional potential (KOs) showing consistency under AN and AU treatment.

a Procrustes analysis showed the significant consistency between the patterns of bacterial community and functional potential in both AN and AU. **b** Preference analysis about the functional potential of bacterial metagenome-assembled genomes (MAGs) indicating the different preference of KOs between bacterial MAGs in AN and AU. The left-bottom quadrant of two-dimensional preference (2*DP*) plot shows the preference of KOs in AN (P_{FDR} < 0.05 and 2*DP* < -1.0), and the right-bottom quadrant shows the preference of KOs in AU (P_{FDR} < 0.05 and 2*DP* > 1.0). **c** Functional enrichment analysis of preferred KOs in the MAGs of AN and AU with hypergeometric tests. The solid circles indicate the significant enriched categories, which have a P-value < 0.05 in a hypergeometric test.

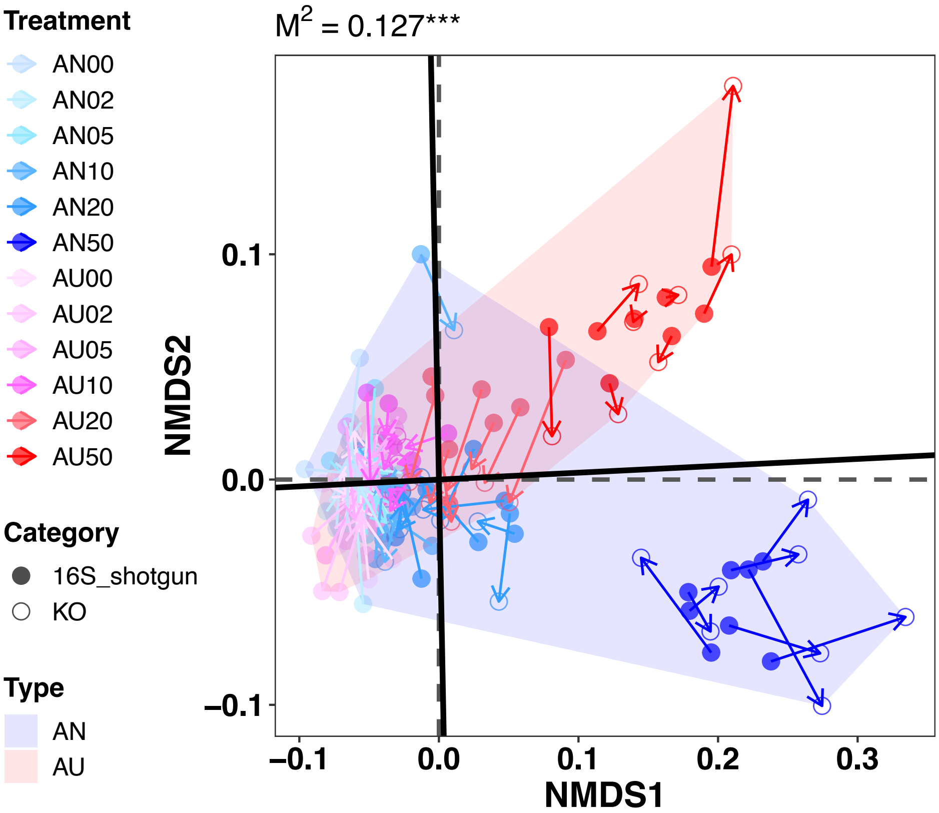

Figure S17. Procrustes analysis showing the significant consistency between the patterns of bacterial taxa and functional potential in both AN and AU.

The bacterial taxa analysis was annotated from metagenomic data. The result was almost the same as Fig. S16a.

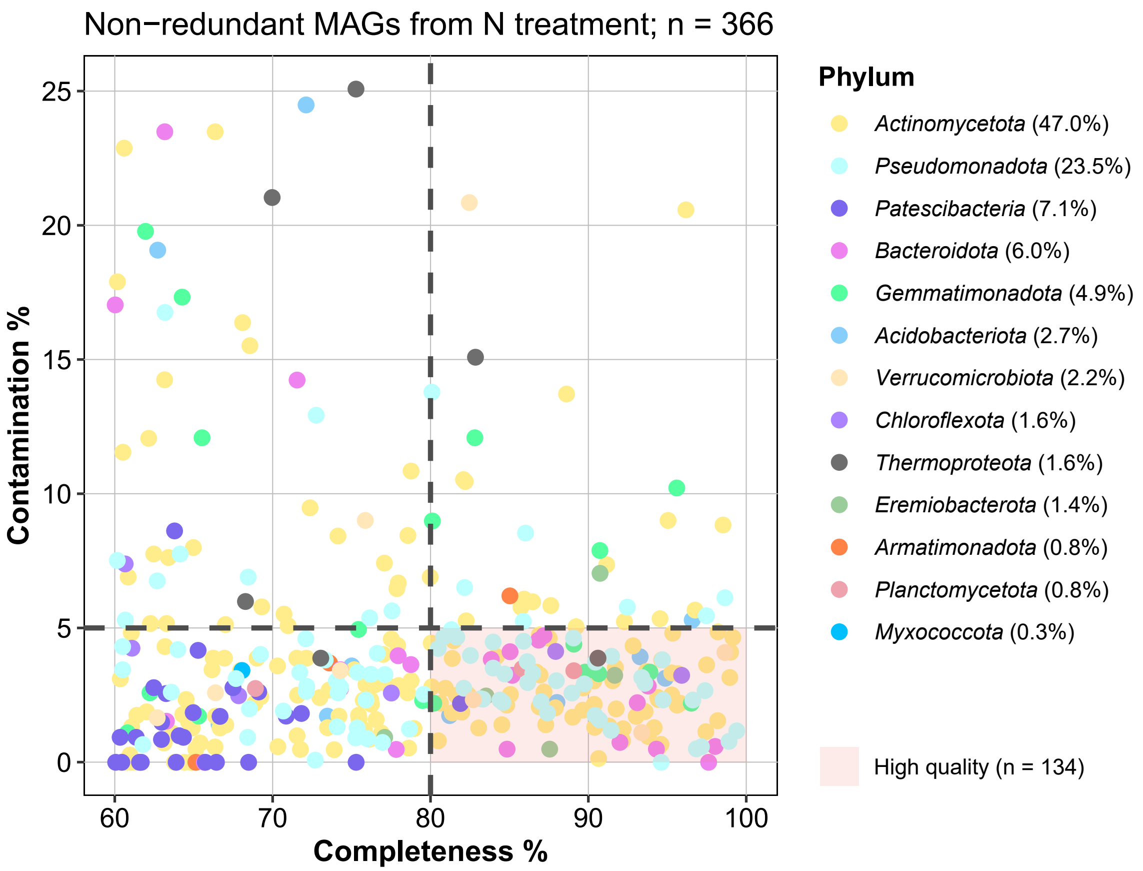

Figure S18. Quality distribution of the 134 non-redundant metagenome-assembled genomes (MAGs) with high quality from metagenomic binning in this study.

The high-quality standard of each MAG in this study was completeness > 80% and contamination < 5%. Note that the 1 archaea MAG (*Thermoproteota*) with high quality was eliminated in the subsequent analysis.

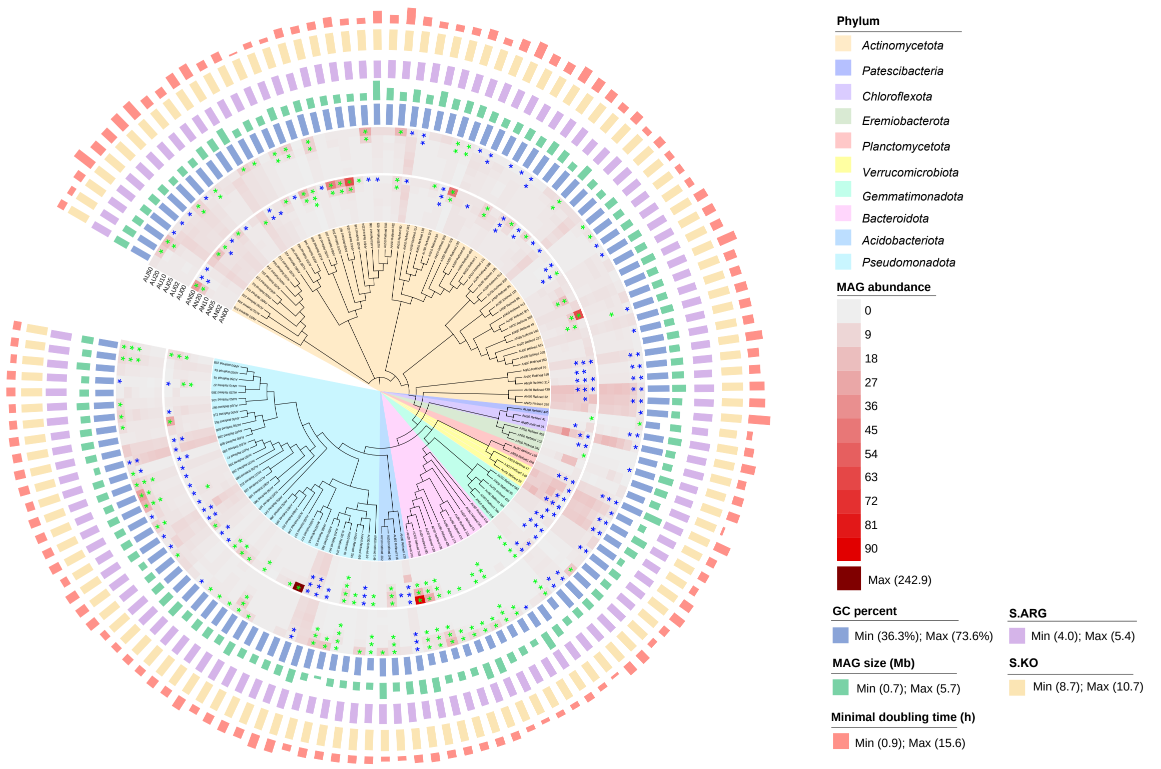

Figure S19. Phylogenetic tree of 133 high-quality (completeness > 80% and contamination < 5%) bacterial MAGs in this study.

The outer five rings of barplots represent the genome size, GC content, diversities of bacterial antibiotic resistance genes (S.ARG), the functional gene diversities (S.KO) and minimal doubling time of each MAG. The inner 12 rings of heatmaps represent the average relative abundance of each MAG under each N level of AN and AU treatment. The green or blue asterisks represent the enrichment or the depletion of one MAG in one N treatment ($P < 0.05$).

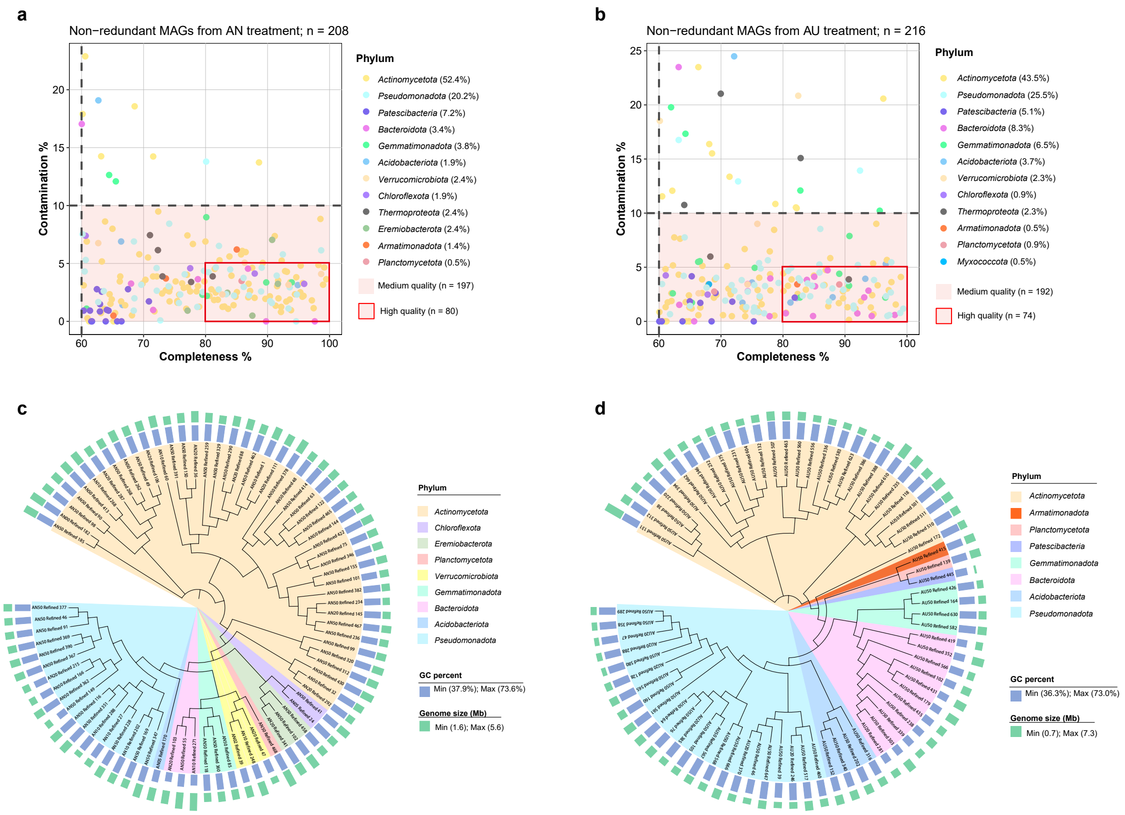

Figure S20. The non-redundant metagenome-assembled genomes (MAGs) in AN and AU treatment.

a,b Quality distribution of the non-redundant MAGs in AN (**a**) and AU (**b**) treatments. The percentages in parentheses following each bacterial phylum represent the proportion of MAGs in each phylum. The medium-quality standard of each MAG was completeness > 60% and contamination < 10%; and the high-quality standard was completeness > 80% and contamination < 5%. Note that only one archaea MAG (*Thermoproteota*, with high quality) was obtained in both AN and AU treatment, which was eliminated in the subsequent analysis. **c** Phylogenetic tree of 79 bacterial high-quality MAGs in AN treatment. **d** Phylogenetic tree of 73 bacterial high-quality MAGs in AU treatment. The outer two rings of barplots represent the genome size and GC content of each MAG.

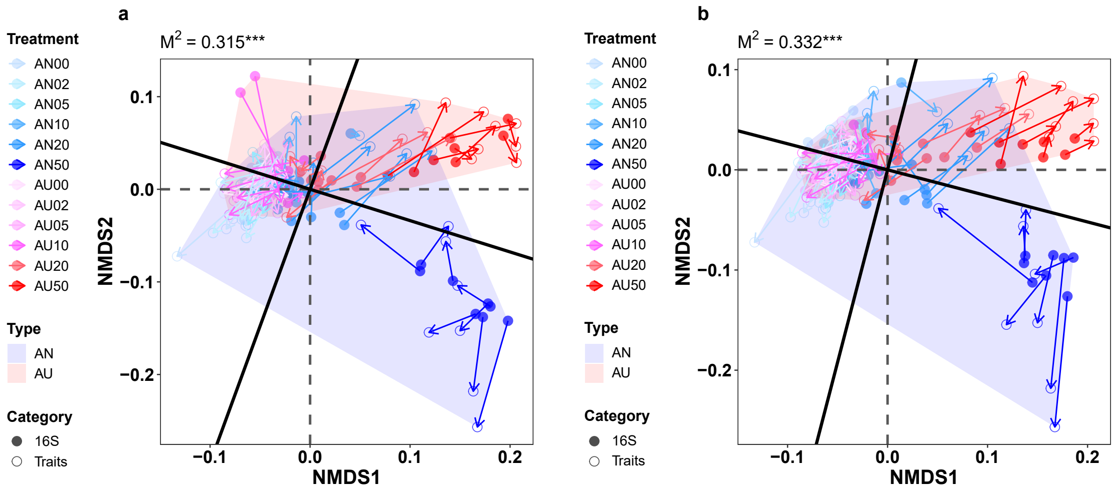

Figure S21. Procrustes analysis showing the significant consistency between the patterns of bacterial taxa and microbial traits in both AN and AU.

The bacterial taxa analysis was annotated from **a** 16S rRNA gene amplicon data and **b** metagenomic data.

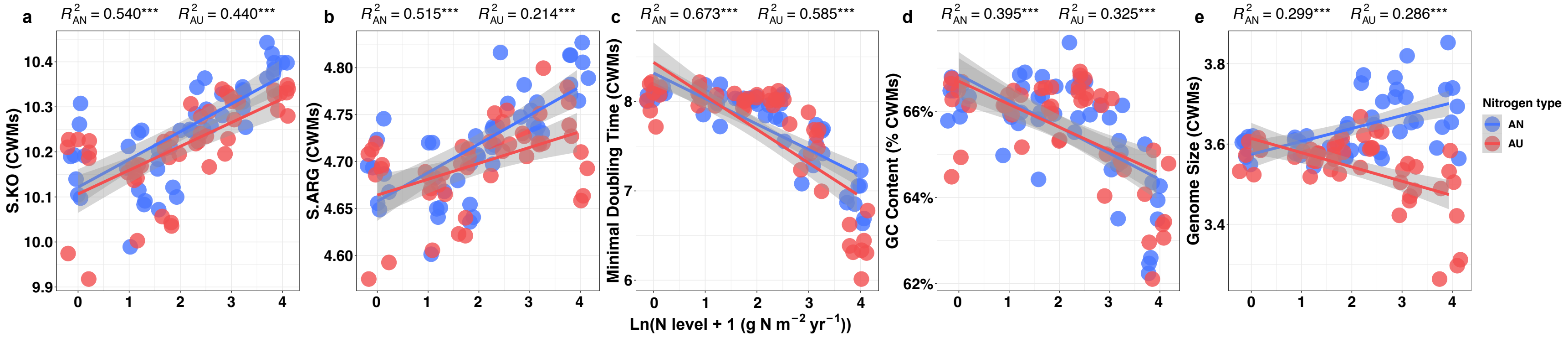

Figure S22. Microbial traits at community level based on MAGs and calculated by community-weighted means (CWMs) method. The blue and red solid lines indicate significant relationships between factors and AN or AU, respectively. The gray area around the smooth line is the 95% confidence interval. ***, $P < 0.001$.

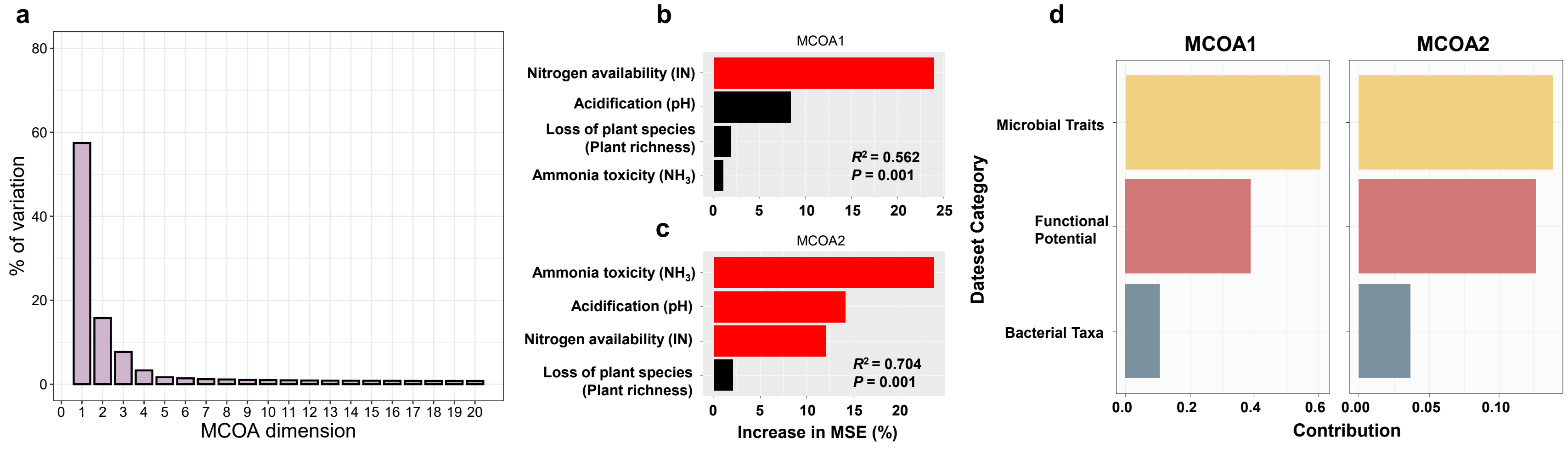

Figure S23. The other supplementary information of MCOA analysis.

a The explanation of MCOA dimensions to the variation. **b-c** The importance of resources and stress represented as the percentage of increase in %MSE (increase of mean square error) with **b** MCOA dimension1 and **c** dimension2 predicted by random forest models. Red bars indicate factors that significantly predict MCOA dimensions and black bars indicate non-significant predicting factors. **d** The contributions of the three datasets to MCOA.

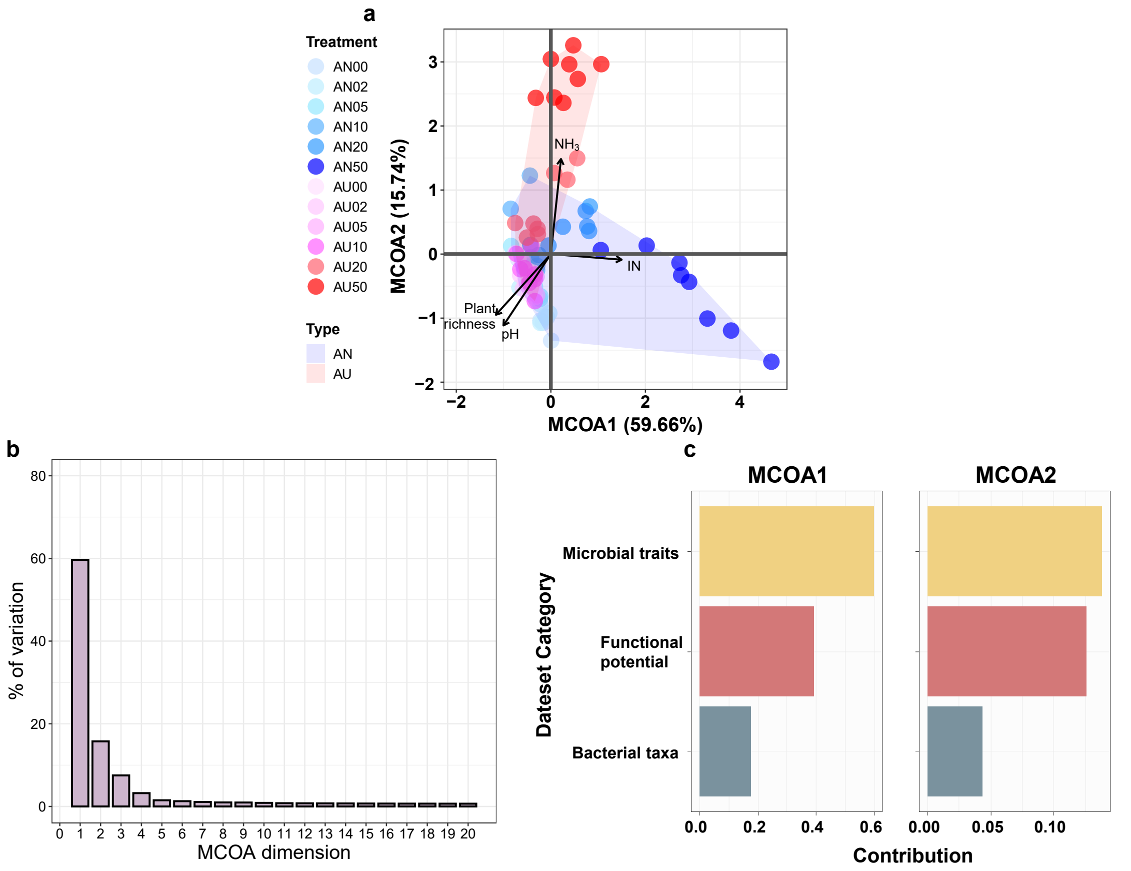

Figure S24. Multitable co-inertia analysis (MCOA) with two-dimensional trait and using the dataset of bacterial community from metagenomic data.

a MCOA depicting consistent trait associations across soil bacterial communities, bacterial functional potential and microbial traits under AN and AU. Dots represented the microbial communities from the 96 samples used in this study along the two dimensions. Significant resource and stress indexes are presented as vectors on the MCOA plot using the “envfit” (based on 999 permutations) at $P < 0.05$. **b** The explanation of MCOA dimensions to the variation. **c** The contributions of the three datasets to MCOA.

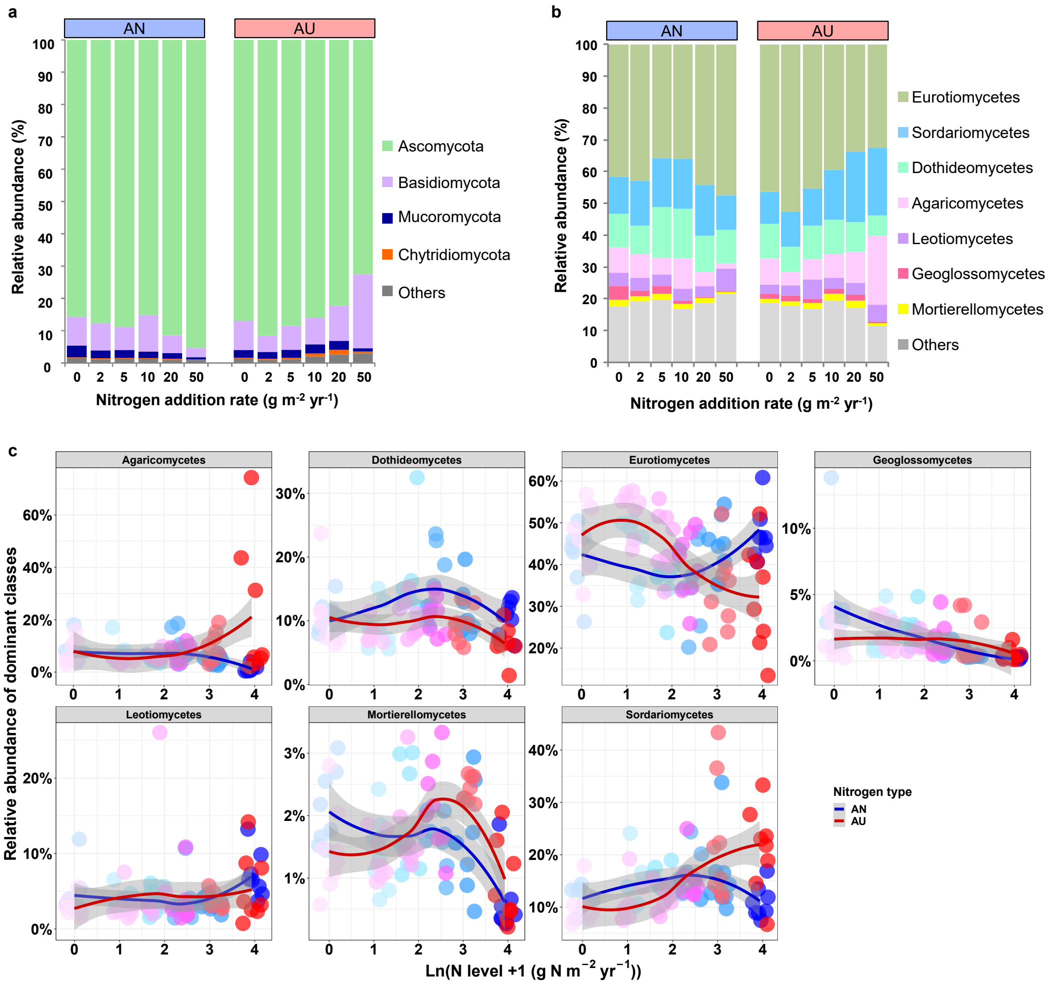

Figure S25. Differences of fungal community composition under AN and AU addition.

a Fungal community composition at phylum level along N addition gradients under AN and AU. **b** Fungal community composition at class level along N addition gradients under AN and AU. **c** Regression curves depicted the relative abundance of fungal dominant classes along N levels under AN and AU. Linear regression model with two-side and adjusted R-squared was used for the statistical analysis.

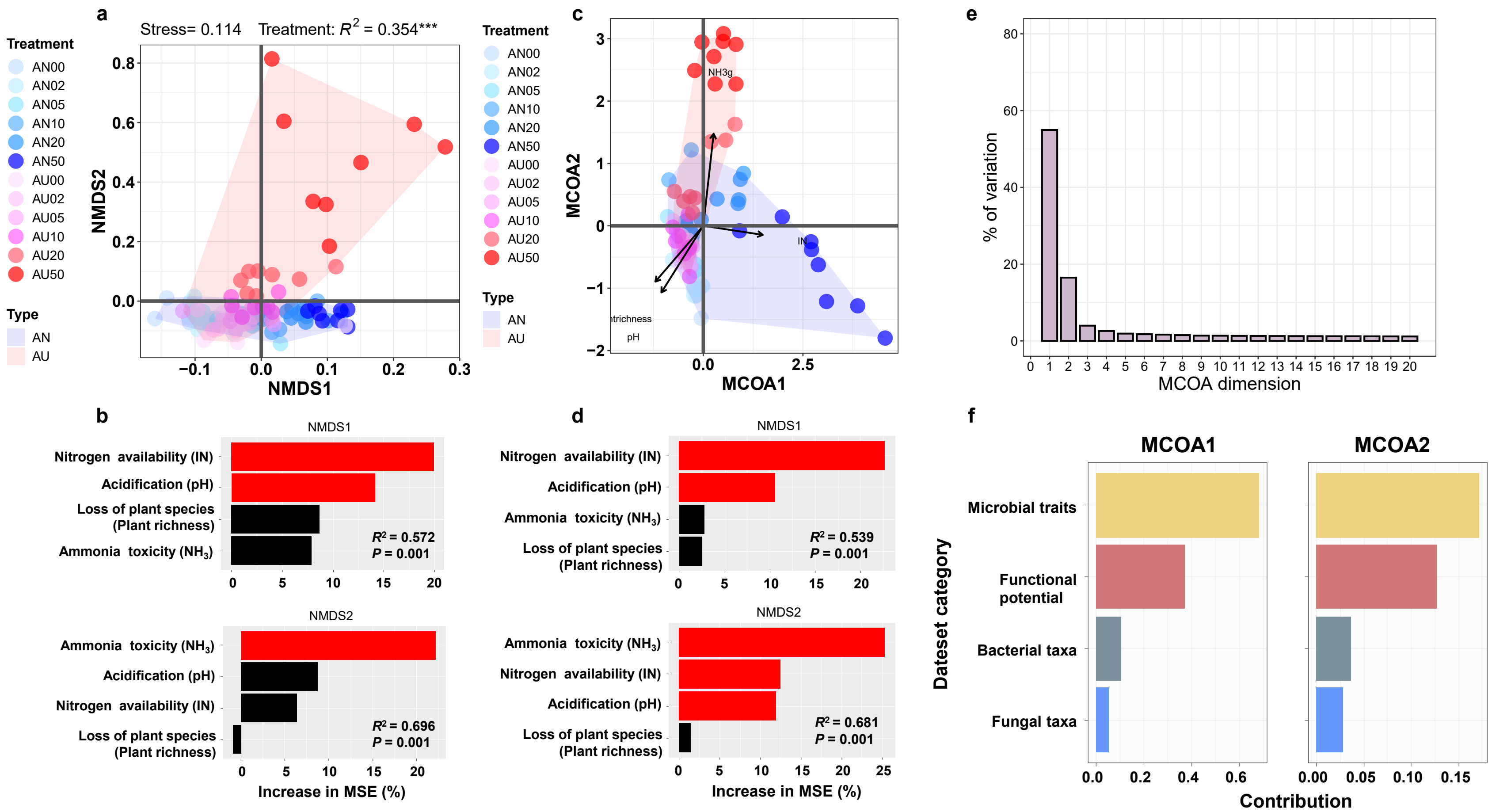

Figure S26. Fungal communities exhibited similar enrichment patterns as bacterial communities under AN and AU addition.

a NMDS analysis of fungal communities with Bray-Curtis dissimilarity and PERM ANOVA showing significant difference between AN and AU. **b** The importance of resources and stress was represented as the percentage of increase in %MSE (increase of mean square

error) with NMDS dimension1 and dimension2 of fungal communities, predicted by random forest models. **c** Multitable co-inertia analysis (MCOA) with two-dimensional trait space depicted consistent trait associations across soil bacterial community composition, fungal community composition, bacterial functional potential, and microbial traits under AN and AU. Dots represented the microbial communities from the 96 samples used in this study along the two dimensions. Significant resource and stress indexes are presented as vectors on the MCOA plot using the "envfit" (based on 999 permutations) at $P < 0.05$. **d** The importance of resources and stress was represented as the percentage of increase in %MSE (increase of mean square error) with MCOA dimension1 and dimension2 predicted by random forest models. Red bars indicate factors that significantly predicted MCOA dimensions and black bars indicate non-significant predicting factors. **e** The explanation of MCOA dimensions to the variation. **f** The contributions of the four datasets to MCOA.

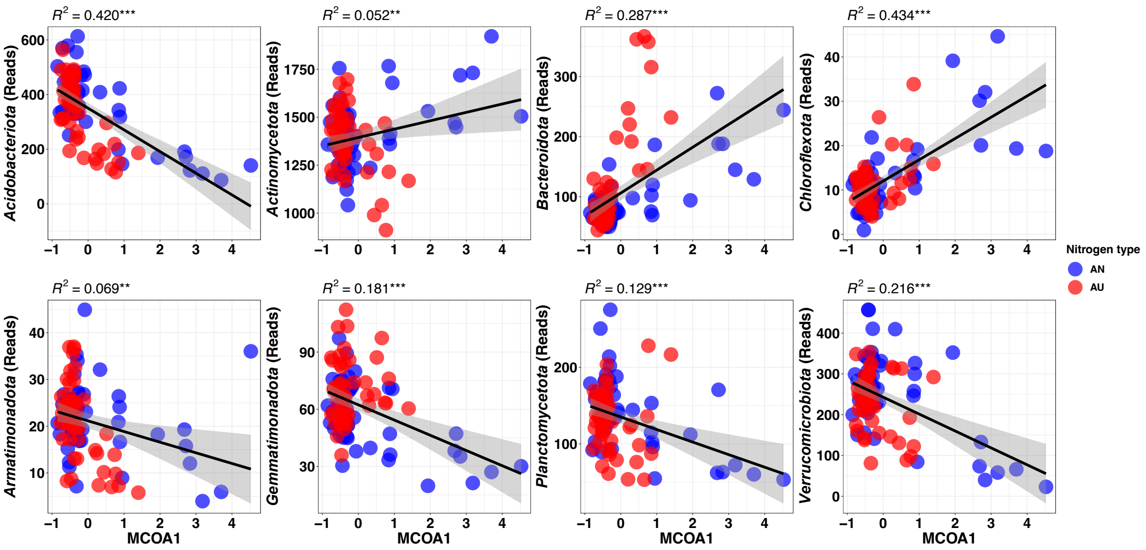

Figure S27. Correlations between bacterial phyla and MCOA1.

Statistical analysis was performed using a linear regression model with two-sided test, and adjusted R^2 values are reported. The gray area around the smooth line is the 95% confidence interval. **, 0.001 < *P* < 0.01; ***, *P* < 0.001

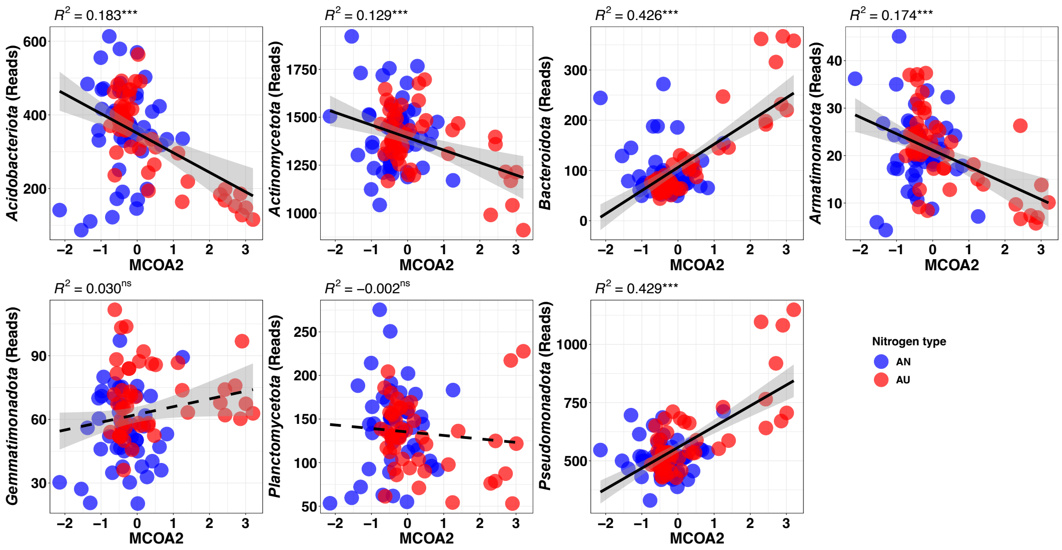

Figure S28. Correlations between bacterial taxa and MCOA2.

Statistical analysis was performed using a linear regression model with two-sided test, and adjusted R^2 values are reported. The gray area around the smooth line is the 95% confidence interval. ns, non-significant; ***, $P < 0.001$

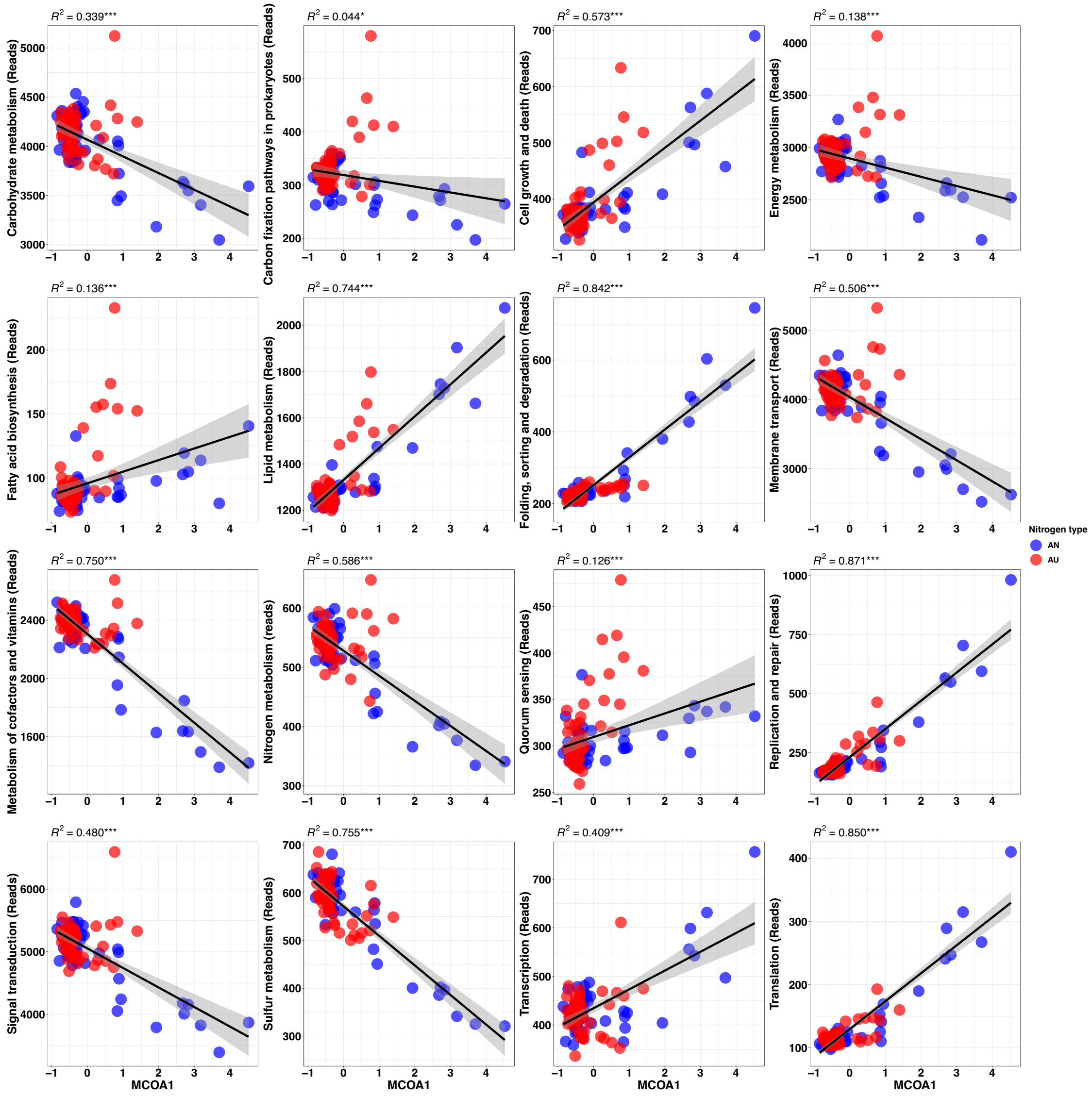

Figure S29. Correlations between functional potential classified into Y-A-S strategies and MCOA1.

Statistical analysis was performed using a linear regression model with two-sided test, and adjusted R^2 values are reported. The gray area around the smooth line is the 95% confidence interval. *, $0.01 < P < 0.05$; ***, $P < 0.001$

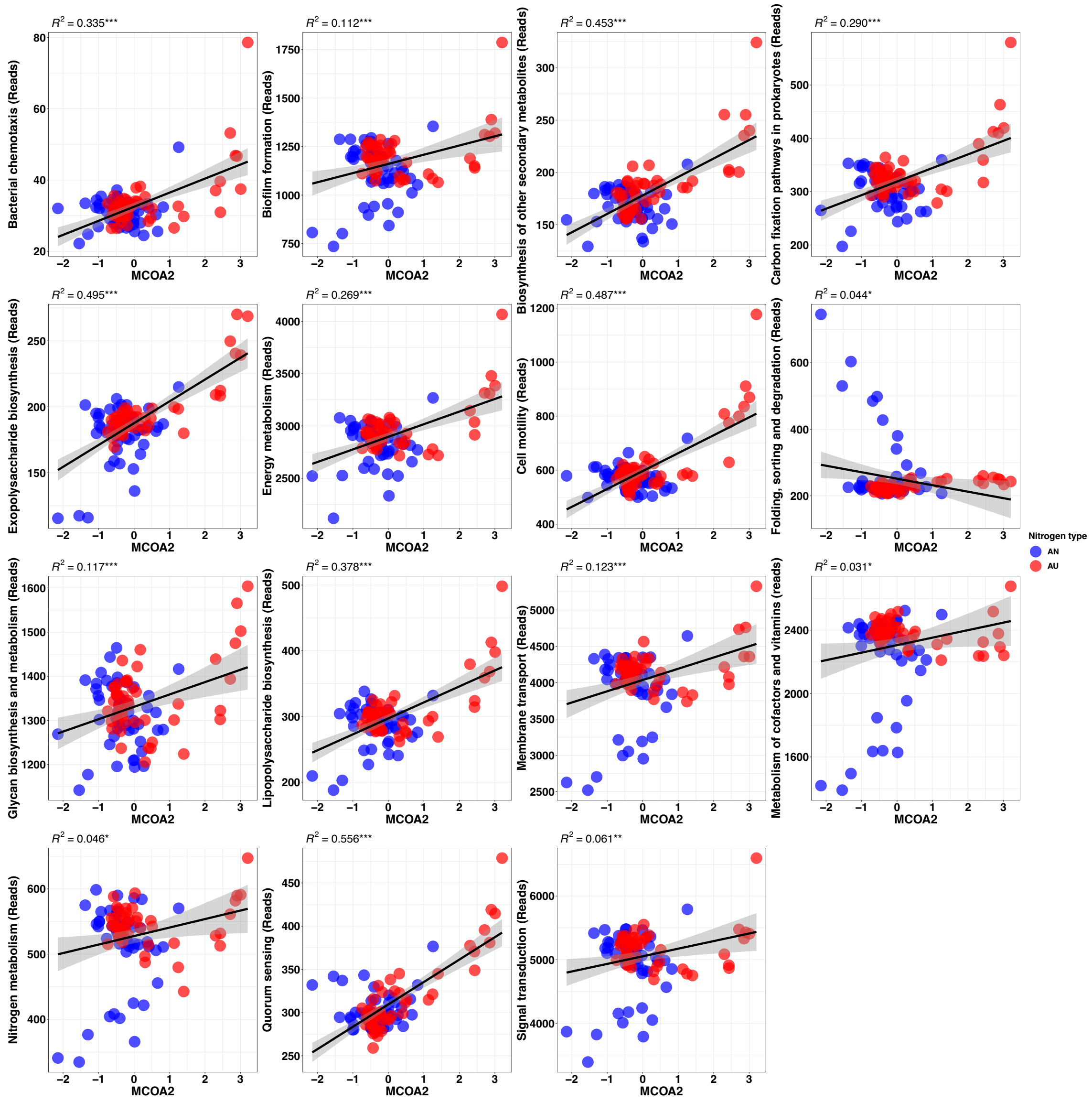

Figure S30. Correlations between functional potential classified into Y-A-S strategies and MCOA2.

Statistical analysis was performed using a linear regression model with two-sided test, and adjusted R^2 values are reported. The gray area around the smooth line is the 95% confidence interval. *, $0.01 < P < 0.05$; **, $0.001 < P < 0.01$; ***, $P < 0.001$

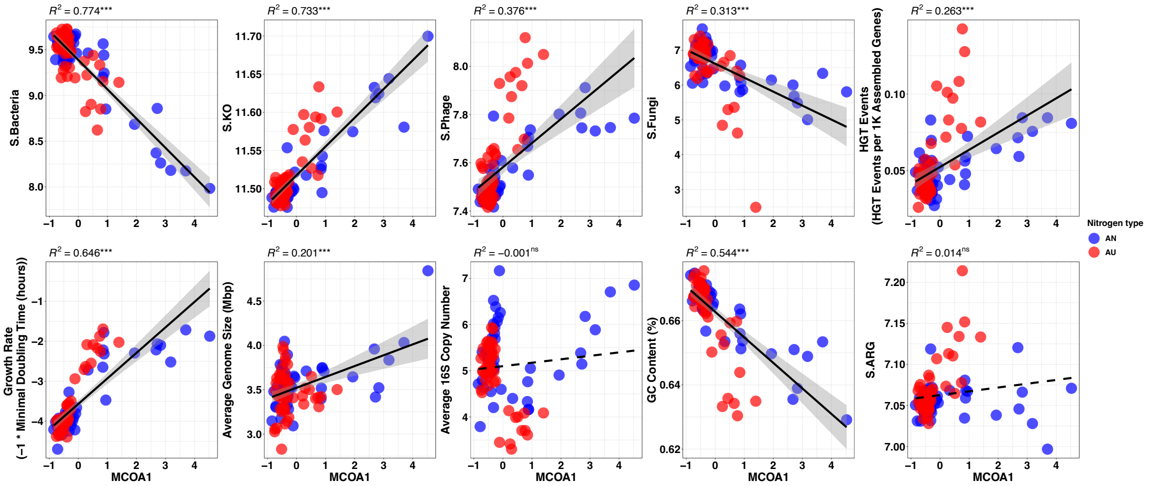

Figure S31. Correlations between microbial traits and MCOA1.

Statistical analysis was performed using a linear regression model with two-sided test, and adjusted R^2 values are reported. The gray area around the smooth line is the 95% confidence interval. ns, non-significant; ***, $P < 0.001$

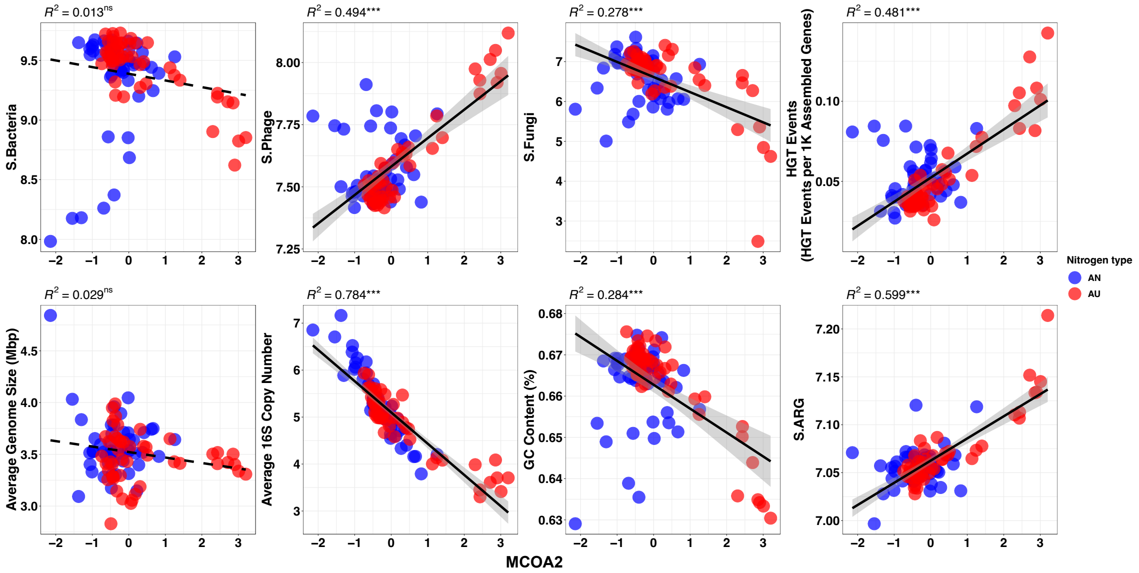

Figure S32. Correlations between microbial traits and MCOA2.

Statistical analysis was performed using a linear regression model with two-sided test, and adjusted R² values are reported. The gray area around the smooth line is the 95% confidence interval. ns, non-significant; ***, $P < 0.001$

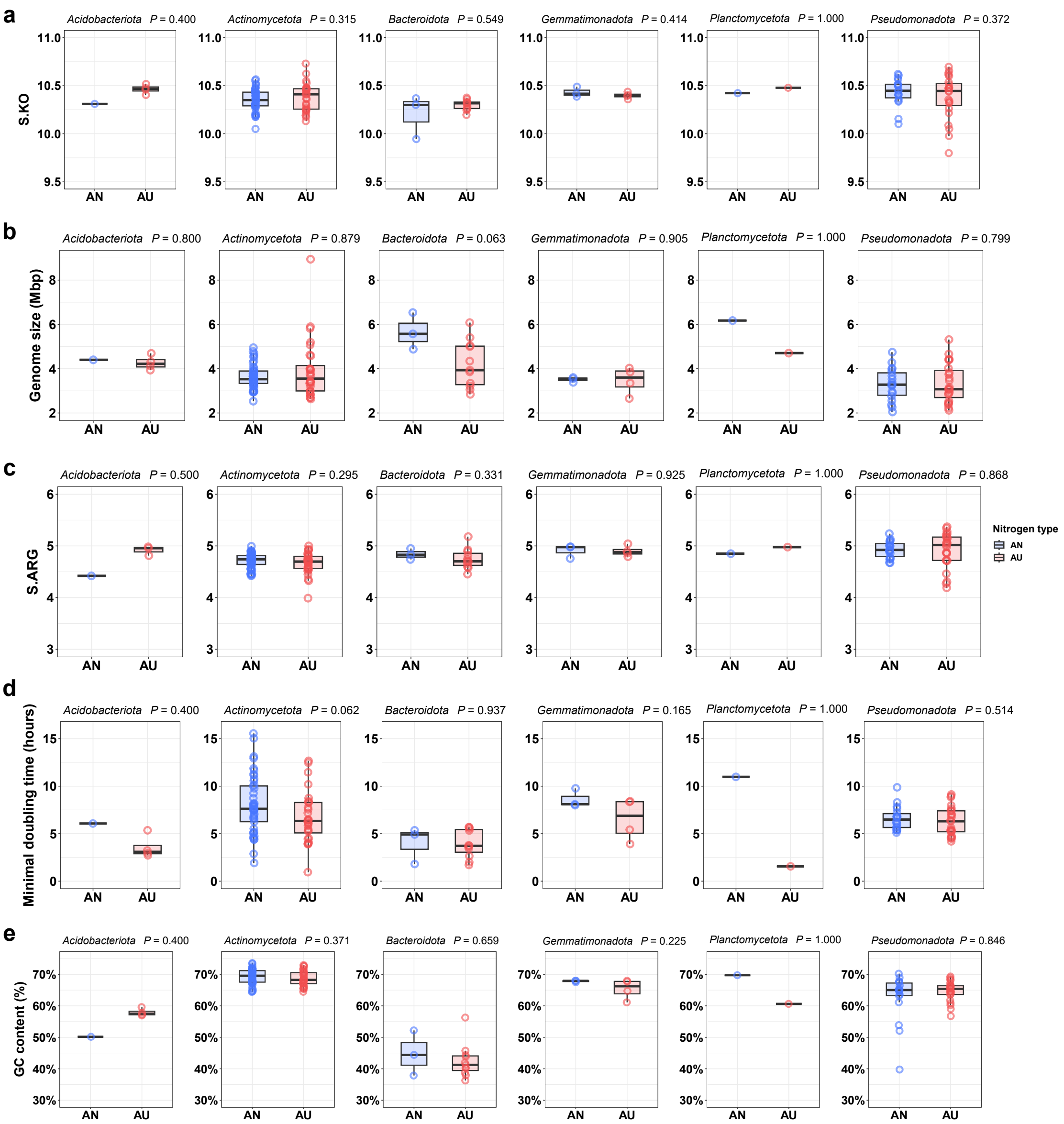

Figure S33. The microbial traits of metagenome-assembled genomes (MAGs) showing no significant difference between AN and AU.

The P-values of *t*-test are showed.

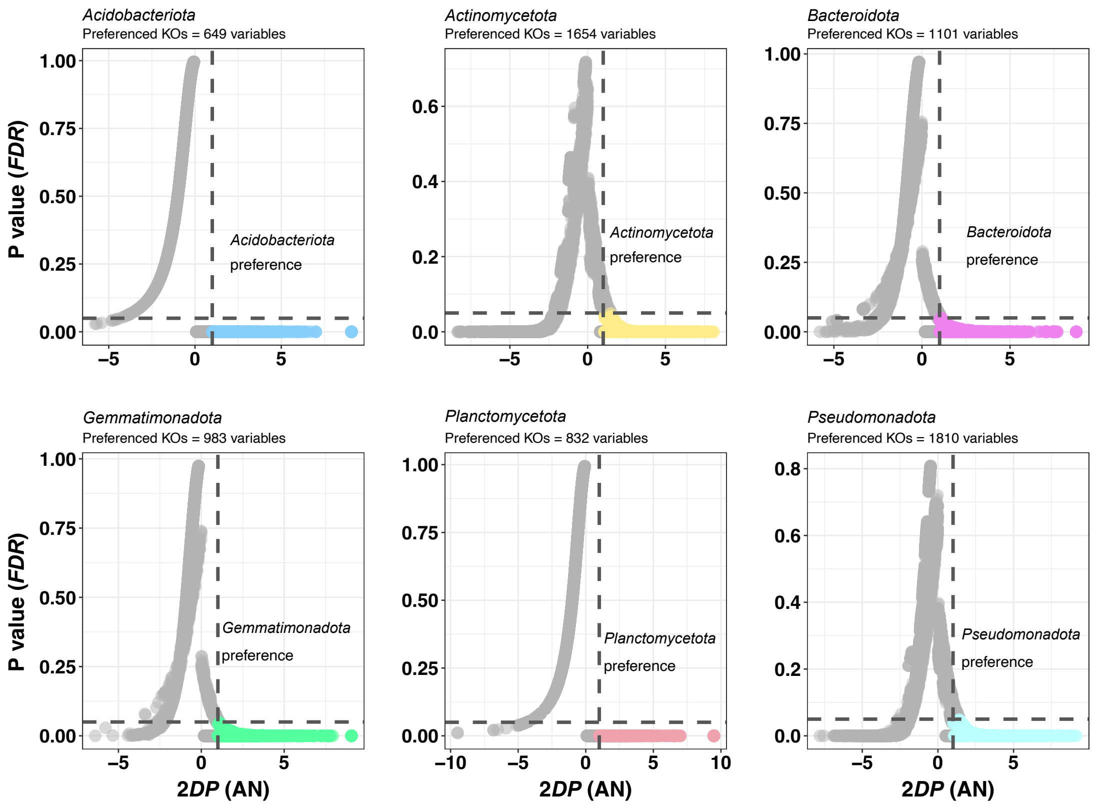

Figure S34. Preference analysis about the functional potential of bacterial metagenome-assembled genomes (MAGs) at phylum level under AN treatment.

The right-bottom quadrant of two-dimensional preference (2*DP*) plot shows the preference of KOs in each phylum ($P_{FDR} < 0.05$ and 2*DP* > 1.0).

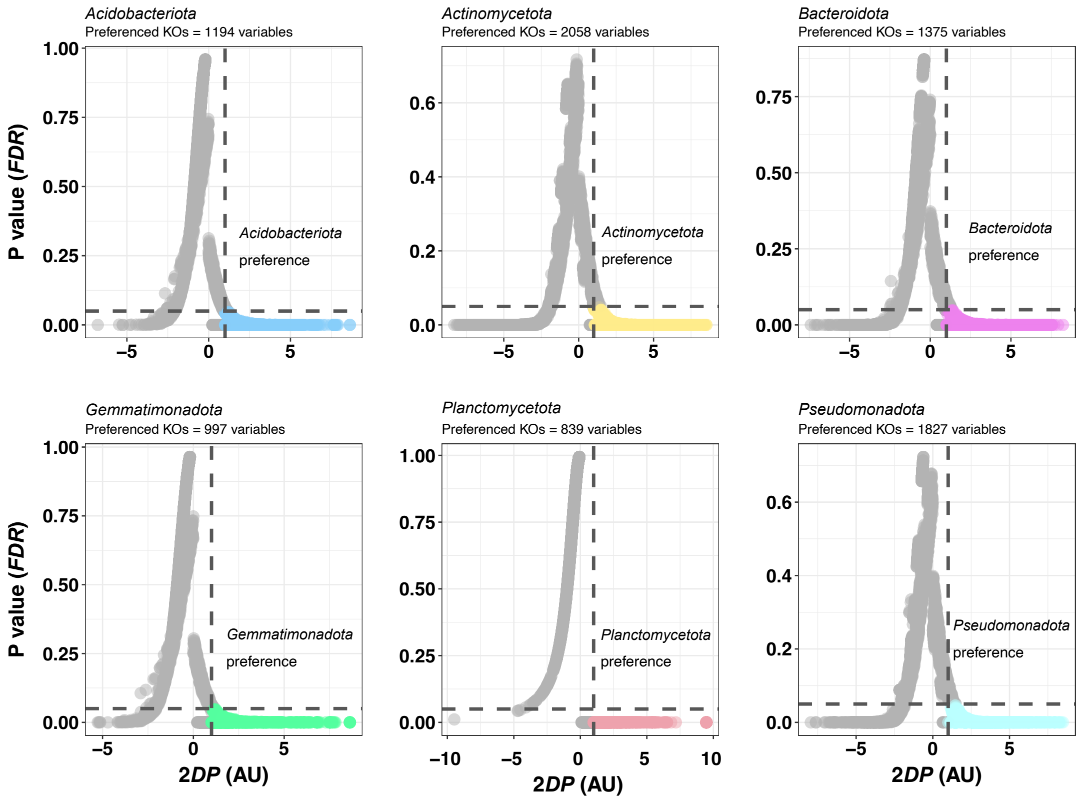

Figure S35. Preference analysis about the functional potential of bacterial metagenome-assembled genomes (MAGs) at phylum level under AU treatment. The right-bottom quadrant of two-dimensional preference (2*DP*) plot shows the preference of KOs in each phylum ($P_{FDR} < 0.05$ and 2*DP* > 1.0).

Table S1. A meta-analysis of ammonia toxicity with the addition of AN and AU.

Reference	System	Soil texture	Soil pH	Measure technique	N addition rate (g m^{-2} yr^{-1})		NH_3 loss (% of N applied)	
					AN	AU	AN	AU
[16]	Wheat field	Calcareous soil	8.44	Semi-open metal cylinder	20	20	4.40	10.60
[17]	No-till corn field	Lyles sandy loam	5.60	Semi-open steel cylinder	16.8	16.8	3.69	30.30
		Bedford silt loam	5.50				1.55	10.71
[18]	Italian ryegrass field	Sand: Silt: Clay =7.3 : 1 : 5.2	6.3	Foam absorber with PTFE	5	5	6.80	21.20
[19]	Coffee crop field	Sand: Silt: Clay =1.1 : 1 : 2.3	6.6	Semi-open static collector	45	45	0.49	18.50
[20]	Bentgrass	Marvyn loamy sand	6.0	Active flow chambers	14.7	14.7	2.00	40.00
[21]	No-till corn field	Hapludox soil	4.5-5.2	Semi-open static collector	15	15	1.00	12.50
[22]	No-till corn field	Sand: Silt: Clay =1 : 1.2 : 2.3	5.4-6.2	Semi-open static collector	10	10	0.80	18.00
							0.80	14.20
[23]	Orchardgrass sod	Xenia silt loam soil	6.0	Semi-open steel cylinder	30	30	5.43	37.70
							3.87	24.20
[24]	Bermudagrass	Captina silt loam	5.3-5.5	Semi-open static chamber	13.3	13.3	0.50	14.30
							0.70	16.90
[25]	Corn field	Plano silt loam	6.0-6.2	Semi-open PVC chambers	11.2	11.2	0.00	8.00
							1.00	18.00
		Fayette silt loam	7.1-7.3				2.00	24.00
							2.00	19.00
	Orchardgrass pasture	Fayette silt loam	6.9-7.3		6.7	6.7	0.00	25.00
							1.00	20.00
[26]	Bean field	Sandy loam	4.9-5.1	Semi-open static chamber	10	10	<1.00	11.00
		Sandy clay loam	4.3-4.5				<1.00	15.30
		Clay	4.5				<1.00	15.50
[27]	Grassland	Sand: Silt: Clay =3 : 1 : 3.1	5.4	Semi-open static chamber	9	9	2.02	13.92
					18	18	3.17	24.37
					27	27	7.71	44.58
[28]	Grassland	Sandy texture	4.5	Semi-open static chamber	10	10	4.88	21.94
					20	20	2.87	21.33
[29]	No-till corn field	Dystrophic red latosol	5.2-5.7	Semi-open PVC chambers	15	15	0.70	39.00
[30]	Coffee orchard	Ferralsol soil	4.5	Semi-open static chamber	45	45	0.30	31.20

	field							
[31]	Coffee plantations	Hapludox soil	4.6	Semi-open PVC chambers	30	30	0.20	23.20
							0.80	24.20
[32]	Sugarcane field	Hapludox soil	4.4-4.9	Semi-open static chamber	5	5	<1.00	18.20
							<1.00	15.10
					10	10	<1.00	23.10
							<1.00	18.30
					15	15	<1.00	24.80
							<1.00	21.80
[33]	Corn field	Acrudox soil	5.5-5.9	Semi-open PVC chambers	15	15	1.00	21.93
							0.67	20.80
							1.07	19.33
							0.60	13.33

Table S2. A list of studies on microbial Y-A-S life history strategies.

Y-A-S life history strategies						
**Reference**	**System**	**Estimation technique and marker**	**High yield (Y)**	**Resource acquisition (A)**	**Stress tolerance (S)**	**Note**
This study	Nitrogen addition	Metagenomic annotation	Fatty acid biosynthesis;<br>Carbon metabolism;<br>Biosynthesis of amino acids;<br>Carbohydrate metabolism;<br>Biosynthesis of nucleotide sugars;<br>Metabolism of other amino acids;<br>Lipid metabolism;<br>Amino acid metabolism;<br>Nucleotide metabolism;<br>Average 16S copy number;<br>Replication and repair;<br>Cell growth and death;	Membrane transport;<br>Cell motility;<br>Bacterial chemotaxis;<br>Nitrogen metabolism;<br>Carbon fixation pathways in prokaryotes;<br>Sulfur metabolism;<br>Metabolism of terpenoids and polyketides;<br>Degradation of aromatic compounds;<br>Carbohydrate metabolism;	Replication and repair;<br>Synthesis of EPS;<br>Biosynthesis of secondary metabolites;<br>Lipopolysaccharide biosynthesis;<br>Biofilm formation;<br>Glycan biosynthesis and metabolism;<br>Degradation of aromatic compounds;<br>Metabolism of terpenoids and polyketides;	Grassland
[34]	Theoretical model	Omics	Central carbon metabolism;<br>Amino acid synthesis;<br>Fatty acid synthesis;<br>Nucleotide synthesis;	Extracellular enzyme production;<br>Motility and chemotaxis;<br>Transporters;<br>Siderophores;	σ factors;<br>Molecular chaperons(GroEL, DnaK);<br>Synthesis of trehalose and glycine betaine;<br>Synthesis of EPS;<br>Synthesis of cell walls;	EPS: exopolysaccharides
[35]	Nitrogen addition	Metagenomic annotation	Fatty acid biosynthesis;<br>Amino acid synthesis;<br>Nucleotide synthesis;<br>DNA replication;<br>Biofilm formation;<br>Cell cycle;<br>Apoptosis;		Replication and repair;<br>Glycan biosynthesis and metabolism;<br>Lipopolysaccharide biosynthesis;<br>Biosynthesis of secondary metabolites;<br>Metabolism of terpenoids and polyketides;	
[36]	Drought	Metagenomic annotation		Carbon fixation pathways in prokaryotes;	LAM biosynthesis;<br>Peptidoglycan biosynthesis;	LAM: Lipoarabinomannan

				Methane metabolism;<br>Cell motility;<br>Bacterial chemotaxis;<br>Sulfur metabolism;<br>Membrane transport;	Two-component system;<br>Lipopolysaccharide biosynthesis;	
[37]	LDG	16S amplicon & PICRUSt2 analysis	rRNA community copy number;	Motility and chemotaxis proteins;	Sporulation proteins;	LDG: Latitudinal diversity gradient (*Sarracenia purpurea*)
[38]	Drought	16S amplicon & PICRUSt2 analysis	Average rRNA copy number;	Carbon acquisition ability (Eco-microplates);	Sporulation protein gene number;	Plastic greenhouse (tomato)
[39]	SQI	Metagenomic annotation		Motility and chemotaxis proteins;	Molecular chaperons(DnaJ);<br>Phage shock protein(PapA);<br>Universal stress response protein (UspA);	SQI: soil quality index (cotton, wheat, pasture and native vegetation)
[40]	Global soil	Metagenomic annotation	Central carbohydrate metabolism;	Lignin degradation;<br>Motility and chemotaxis proteins;<br>Transport system;	Lipid metabolism;<br>Lipopolysaccharide metabolism;	
[41]	Tillage	Metagenomic annotation			Synthesis of EPS;	Cropland
[42]	Genome data	COG and KEGG annotation			σ factors (σD, σS, σH, σF, σB, σN and extracytoplasmic σ factors);	2,518 bacterial and 202 archaeal genomes
[43]	Drought	Omics			Synthesis of trehalose;	Review;<br>Plants, rhizobia, AMF
[44]	Genome data	CAZy and KEGG annotation	Amino acid related enzymes;<br>Lipid biosynthesis proteins;<br>Lipopolysaccharide biosynthesis proteins;	Genes for CAZymes;	Chaperones;<br>Folding catalysts;<br>The prokaryotic defense system;<br>Peptidoglycan biosynthesis and degradation proteins;	440 *Sphingomonas* genomes from different sources
[45]	Salinity & nutrient	Metagenomic annotation	Amino acid metabolism;<br>Carbohydrate metabolism;<br>Cell processes(cell cycle-Caulobacter, pyrimidine metabolism and RNA polymerase);	Alkaline phosphatase activity;<br>Transporters;<br>Flagellar assembly;	Homologous recombination;<br>Base excision repair;<br>Biofilm formation;<br>Extracellular polysaccharide biosynthesis;<br>Osmolytes production;	Coastal saline soils

					Transcription;<br>Translation;<br>Folding, sorting and degradation	
[46]	Warming	Metatranscriptomic annotation	Carbohydrate metabolism;<br>Amino acid metabolism;<br>Microbial biomass;	Cellulase, xylan, chitin, and hemicellulose hydrolases;	Chaperones (GroEL, Hsp20);	Grassland
[47]	pH and nutrient	Metagenomic annotation		High GC content;	large genome;	398 soil samples across a broad geographic range
[48]	Genome data	Metagenome assembled genomes (MAGs)		Nitrogen metabolism;		>10,000 MAGs from the Genomic Catalog of Earth's microbiomes (GEMs) dataset and NCBI RefSeq database
[49]	pH	Metagenomic annotation	Growth rate;<br>Average 16S rRNA gene copy numbers;	Genes for CAZymes;<br>Carbohydrate metabolism;<br>Energy production and conversion;	GC content;	176 soils from 11 terrestrial biomes across 6 continents
[50]	Review	Review		Metabolism of terpenoids and polyketides;	Metabolism of terpenoids and polyketides;	
[51]	Review	Review		Degradation of aromatic compounds;	Degradation of aromatic compounds;	
[52]	Nutrient	Experiment and theory		Cell motility;<br>Bacterial chemotaxis;		

Table S3. A list of studies on microbial traits.

Nutrient addition / Acidification / Ammonia toxicity stress													
**Refer -ence**	**System**	**Research factors**	**S.Bacteria**	**S.Fungi**	**HGT events**	**S.Phage**	**Growth rate**	***rrn***	**S.ARG**	**GC %**	**Genome size**	**Functional diversity**	**Note**
This study	Grassland	AN / AU	↓^{2} / ↓	↓ / ↓	↑ / ↑^{2}	↑ / ↑^{2}	↑ / ↑	ns / ↓	ns / ↑	↓ / ↓	↑ / ns	↑^{2} / ↑	**HGT**: WAAFLE; **Growth rate**: gRodon[53]; ***rrn* copy number**: Pereira-Flores et al[54]; **GC content**: quast[55]; **ARG diversity**: Resfams database[56]; **Genome size**: MicrobeCensus[57]
[58]	Forest	pH↓	↓				ns	ns	↑	↓	↑	↑	**Growth rate**: gRodon[53]; ***rrn* copy number**: Pereira-Flores et al[54]; **GC content**: quast[55]; **ARG diversity**: Resfams database[56]; **Genome size**: MicrobeCensus[57]
[59]	Grassland	AN	↓		↑	↑					ns	↑	pH: ↓; **HGT**: WAAFLE; **Genome size**: MicrobeCensus[57]
[60]	Grassland	AU / AU+SP	ns / ns	ns / ↓			ns / ↑				↓ / ↓	ns / ns	25 global grasslands of Nutrient Network experiment; pH: ↓; **Growth rate**: matching 16S rRNA gene sequences to published genomes; **Genome size**: estimated from published genomes
[61]	Grassland	AN	↓										pH: ↓
[62]	Cropland	AU	↓										pH: ↓
[63]	Grassland	AN	↓										pH: ↓

[64]	Grassland	ANU	↓	↓									pH: ↓
[65]	Meta-analysis	N	↓										pH: ↓
[66]	Meta-analysis	N	↓	↓									pH: ↓
[67]	Forest	AU	↓	↓ (Topsoil) ↑ (Subsoil)									pH: ns; Short-term N application
[68]	Meta-analysis	N	↓	↓									pH: ↓
[69]	Grassland	AU	↓					↑		↑			pH: ↓; ***rrn* copy number:** rrnDB database; **GC content:** annotating 16S representative sequence to GTDB
[70]	Cropland	AU	↑										pH: ↓ (slightly)
[71]	Forest	AN	↓										pH: ↓
[72]	Meta-analysis	N	↓	↓									pH: ↓ (slightly)
[73]	Grassland	AU	↓					↑					pH: ↓; ***rrn* copy number:** rrnDB database
[74]	Forest	AN	↓										pH: ↓
[75]	Forest	AN	↓										pH: ↓
[76]	Cropland	AU	↓										pH: ↓
[77]	Grassland	AU	↓	↓									pH: ↓
[78]	Microcosm	AN	↓										Nature soils: wetland; Function: extracellular enzyme activity
[79]	Forest grassland ecotone	AU	↓	ns									pH: ↓
[80]	Grassland	AU	↓	ns									pH: ↓

[81]	Grassland	AS	↓										60 samples of the Park Grass Experiment; pH: ↓ (AS addition)
[82]	Grassland	AN	ns										pH: ↓; 10 g N m^{-2} y^{-1}; AU in 2005, AN in 2006-2010
[83]	Arid shrubland	AN	↓	ns									Catabolic diversity↑
[84]	Grassland & cropland	AN	ns										
[85]	Grassland	AN	↓										pH: ↓
[86]	Grassland	AU	↓	↓									pH: ↓
[87]	Grassland	AN	↓										pH: ↓ (slightly)
[88]	Forest	AN	↑										pH: ↓
[89]	Wheat rhizospher e	AN	↓									ns	pH: ↓; **Functional diversity**: predicting with Tax4Fun[90]
[91]	Pot experimen t	AU	↓										Nature soils: *B. ischaemum*-dominated natural grassland soils; Short-term N application; pH: ns; *B. ischaemum* seeds
[92]	Cropland	AU	↓	↓									pH: ns;
[93]	Naked rock	AN	↓										pH: ↓
[94]	Cropland	N	ns	↓ (slightly)									pH: ↓(black and red soils); pH: ns (fluvo-aquic soil)
[95]	Forest	AN	ns	ns									pH: ↓
[96]	Permafrost peatland	AN	ns	ns									PH: ns
[97]	Grassland(g) & cropland(c)	AN	g: ns c: ↓										pH: ↓
[98]	Grassland	AN	↓	ns									pH: ↓; Qinghai-Tibetan Plateau
[99]	Cropland	AN	↓	↑									pH: ↓; AN:1967-2007, AU: 2008;

[100]	Forest	AU	↓										pH: ↓
[101]	Cropland	AU+PK	↓										pH: ↓
[102]	Grassland	AN	↓										pH: ↓
[103]	Mesocosm	AN	↓										Nature soils: mangrove soils; pH: ↑
[104]	Meta-analysis	N	↓	↓									pH: ↓; Nature soils: forest, grassland, cropland, shrubland and tundra
[105]	Forest	AN	ns	ns (R) ↑ (B)									pH: ns; R: rhizosphere; B: bulk soil
[106]	Cropland	AU	↓										pH: ↓
[107]	Biocrust	AN	↑	↑									Liudaogou watershed; Short-term N application
[108]	Karst ecosystem	pH↓	↓										Forest, cropland, and Heshang cave soils
[109]	Cropland	pH↓	↓										
[110]	Mollisol	pH↓	↓										Nitrogen fertilizers causing pH decline
[111]	Saline soils	pH < 8.2	↓										Ebinur Lake; pH >7
[112]	Nature soils	pH↓	↓										98 soil samples from across North and South America
[113]	Wetland	pH↓	↓										
[114]	Nature soils	pH↓	↓									↓	Nature soils: cold deserts, hot deserts, forests, grasslands, and tundra
[115]	Nature soils	pH↓	↓										Soil samples collected from 237 locations across a wide range of ecosystem types
[116]	Meta-analysis	pH↓	↓										3,986 samples mostly collected from soil and freshwater of Earth Microbiome Project
[117]	Nature soils	pH↓	↓	↓									615 composite samples of topsoil from all continents

													and climates
[118]	Nature soils	pH↓	↓										1,000 soils collected across the UK
[119]	Cropland	pH↓	↓	↓ (slightly)									Hoosfield acid strip
[120]	Nature soils	pH↓	↓										Soil samples collected from across North and South America
[121]	River sediment	pH↓	↓										Qiantang River
[122]	Cropland	pH↓	↓										
[123]	Meta-analysis	pH↓	↓										Natural soils: forests in China
[124]	Forest	pH↓	↓										Natural soils: tea garden
[125]	Nature soils	pH↓	↓										a 1,200 m elevation gradient on Huangshan, 6 typical vegetation types
[126]	Microcosm	pH↓	↓										Plastisphere bacterial communities
[127]	Forest	pH < 5	↓										
[128]	Forest and grassland	pH↓	↓										
[129]	Cropland	pH↓	↓										
[130]	The intestines of *L. vanmei*	NH_3 stress	↓										
[131]	Phyllosphere	NH_3 stress	↓	ns									*S. octophylla* phyllosphere
[132]	Anaerobic digestion	NH_3 stress	↑-↓										
[133]	UASB reactor	NH_3 stress	↓										UASB: anaerobic sludge blanket

[134]	Water sample	NH_3 stress	↓										
[135]	Swine manure	NH_3 stress	↑		↑								**HGT events**: MGEs & ARGs
[136]	Grassland	AU		↓									pH: ↓
[137]	Grassland	AU	ns (Topsoil)<br>↑ (Subsoil)	↓ (Topsoil)<br>↓ (Subsoil)									pH: ↓
[138]	Grassland	AU	↓	↓									pH: ↓
[139]	Grassland	AU	↓	↑-↓									RA of saprotrophs: ↑;<br>pH: ↓
[140]	Grassland	AU		↓									pH: ns
[141]	Cropland	AU		↓									34-year application of N;<br>pH: ↓
[142]	Grassland	AU		↓									pH: ↓
[143]	Grassland	AN		↑									pH: ↓
[144]	Forest	AN		↑ (S)<br>ns (L)									S: short-term N addition (2 years);<br>L: long-term N addition (13 years);<br>pH: ↓
[145]	Grassland	AU		ns									pH: ns;<br>Short-term N application
[146]	Grassland	AU		↑-↓									pH: ↓
[147]	Grassland	AN	ns	↑									pH: ns
[148]	Grassland	AN		ns									pH: ↓
[149]	Grassland	AU	↓	↓									
[150]	Desert	AN	ns	ns									pH: ns
[151]	Forest	AN	↓	ns									pH: ↓
[152]	Grassland	AN		↑									pH: ↓
[153]	Grassland	AN	ns	ns									Short-term N addition (2 years);<br>pH: ns
[154]	Forest	AU	↓	ns									pH: ↓ (slightly);

													Short-term N application
[155]	Bamboo forest	AN	ns	ns									O: organic horizon;<br>M: organic horizon;<br>pH: ↓ (O)
[156]	Meta-analysis	N	↓	ns									pH: ↓
[157]	Forest	AN	ns (CNA)<br>ns (CNAUR)	↓ (CNA)<br>ns (CNAUR)									CNA: canopy N addition;<br>UR: understory removal;<br>Soil layer: 0-10cm;<br>pH: ns
[158]	Poplar plantation	AN		↓									pH: ↓
[159]	Nature soils	pH↓		↑-↓ (unimodal)									1251 plots in the northern Baltic region
[160]	Nature soils	pH↓		↓									a 1,000 m elevation gradient on Mount Nadu
[161]	Nature soils	pH↓		↓									a 1,000+ m elevation gradient on Mount Shegyla
[162]	Biological soil crusts	pH↓		↑									Ice-free regions in coastal areas of Victoria Land, Antarctica
[163]	Global topsoil	pH↓		↓									365 sites worldwid
[164]	Forest	pH↓	↓	↓									a 1,000+ m elevation gradient in South Tyrol, the Italian Alps
[165]	Ground-water	pH↓		↓ (slightly)									Uranium and nitrate contamination
[166]	Forest	pH↓	↓	↓									karst and non-karst forests in southwest China
[167]	Forest	pH↓		↑									a 400 m elevation gradient in a *Quercus aliena* var. *acuteserrata* forest of Mount Qinling
[168]	Alpine-gorge region	pH↓		↓									a 2,500+ m elevation gradient on the Eastern Qinghai-Tibetan Plateau
[169]	Forest	pH↓		ns									a 1,300 m elevation

													gradient in a *Abies georgei* forests of Mount Segila
[170]	Cropland	pH↓	↓	ns									a 700 m elevation gradient in Yunnan-Kweichow Plateau
[171]	Nature soils	NH_3 stress		ns									the area affected by the N fertilizer factory (JSC Achema) in central Lithuania
[172]	Riparian area	pH↓	ns		↓								**HGT events**: MGEs & ARGs
[173]	Activated sludge	pH↓			↑								**HGT events**: MGEs & ARGs
[174]	Forest and grassland	pH↓				ns				ns			**Phage diversity:** Elution and collection; Correlations were tested by us
[175]	Seawater	pH↓				ns							**Phage diversity:** VirSorter2 and checkV
[176]	Cropland	AU				↑							pH: ↓; **Phage diversity:** Virus-like particles morphological categorization and reciprocal Simpson's index (1/D)
[177]	Antarctic soil	pH↓	ns			↑							**Phage diversity:** Elution and collection; Correlations were confirmed by us
[178]	Stormwater retention pond water	pH↓	↓			ns							**Phage diversity:** Centrifugation and collection
[179]	Various land use types	pH↓				ns							Soils: forest, paddy field and vegetable field, urban park and road verge in China; **Phage diversity:** VIBRANT
[180]	Cropland	pH↓				↑							Nature soils: *Radix pseudostellariae*

													rhizosphere soils;<br>**Phage diversity**: RNA sequence searching with the NCBI virus reference genome database
[181]	Cropland	AU	↑			↓							**Phage diversity:** RAPD-PCR
[182]	Microcosm	AS+G					↑	↑			↓		Natural soils: grassland, woodland & forest;<br>**Growth rate**: the rate of DNA synthesis(qSIP with $H_2{}^{18}O$)[183];<br>***rrn* copy number & Genome size**: best BLAST hit algorithm;
[184]	Microcosm	AN					↓						Natural soils: grassland and sandy-loam soil;<br>**Growth rate**: leucine incorporation[185, 186];<br>Short-term N application
[187]	Microcosm	AN					↓						Natural soils: forest, cropland and grassland;<br>**Growth rate**: thymidine incorporation[186, 188];<br>Short-term N application
[189]	Microcosm	AN					↓						Natural soils: forest;<br>**Growth rate**: thymidine incorporation[186, 188]
[190]	Microcosm	AN					↓						Natural soils: forest;<br>**Growth rate**: leucine incorporation[185, 186]
[191]	Microcosm	pH					↓						Natural soils: cropland;<br>**Growth rate**: thymidine incorporation[186, 188]
[192]	Microcosm	pH					↓						Natural soils: Hoosfield acid strip;<br>**Growth rate**: leucine incorporation[185, 186]
[193]	Microcosm	pH					↓						Natural soils: grassland and cropland;<br>**Growth rate**: DNA-^{13}C/DNA-C (DO^{13}C)
[194]	Microcosm	AN /					ns / ↑						Natural soils: garden soil;<br>Short-term N application;

		AN+G											**Growth rate**: thymidine and leucine incorporation[185, 186, 188]
[195]	Microcosm	AU	↓				↑	↑					Natural soils: grassland ; pH: ns; Short-term N application; **Growth rate**: estimating by population size; ***rrn* copy number**: predicting with rrnDB[196];
[197]	Arid ecosystem	Soil available N↑					↑	↑		↓	ns		Vegetated soils having a higher nitrogen availability than bare soils; ***rrn* copy number**: Pereira-Flores et al[54]; **Growth rate**: Sara Vieira-Silva et al[198]; **GC content**: Albert Barberan et al[199]; **Genome size**: Pereira-Flores et al[54]
[200]	Microcosm	AN					↓ (open grass)						Natural soils: oak-savanna; **Growth rate**: incorporation of $H_2^{18}O$ [201]; Short-term N application
[202]	Microcosms	Alfalfa / pH					↑ / ↓						Natural soils: Hoosfield acid strip; **Growth rate**: leucine incorporation[185, 186]
[203]	Microcosms	pH					↓						Natural soils: vineyards soil; **Growth rate**: thymidine incorporation[186, 188]
[204]	Microcosms	AS / pH					ns / ↓						Natural soils: grassland; **Growth rate**: leucine incorporation[185, 186]
[205]	Microcosms	pH					↓						Natural soils: forest; **Growth rate**: thymidine incorporation[188]
[206]	Microcosms	pH					↓						Natural soils: forest; **Growth rate**: thymidine

													and leucine incorporation[185, 188]
[207]	Microcosms	pH					↓						Natural soils: forest; **Growth rate**: thymidine incorporation[188]
[208]	Grassland	AN					↑						Natural soils: grassland; **Growth rate**: leucine incorporation[185, 186]; Short-term N application
[209]	Microcosms	NH_4^++ PO_3^-	↑					↓					Natural soils: coastal sediment; ***rrn* copy number**: predicting with rrnDB[196];
[210]	Triplicate anaerobic digesters	Poultry waste	ns					↑				↑	Substrate: dairy manure; ***rrn* copy number**: predicting with rrnDB[196]; **Functional diversity**: GeoChip
[211]	Grassland	$Ca(NO_3)_2$	ns					↑				ns	***rrn* copy number**: predicting with rrnDB[196]; **Functional diversity**: GeoChip
[212]	Grassland	Slow-release NPK(AU)						↑			ns		***rrn* copy number and genome size**: trait estimation method[212]
[213]	Postglacial ecosystems	pH↓ / Soil available resource ↑						↓ / ↑					***rrn* copy number**: Diana R Nemergut et al[214]
[215]	Microcosms	NH_4Cl+MP						↑					Natural soils: coastal sediment; ***rrn* copy number**: predicting with rrnDB[196]
[216]	Black, red and yellow soils	Soil total N↑ / PH↓							↑ / ↓				Experiment: Crucian carp corpse decomposition; **ARG diversity**: HT-qPCR array

[217]	Cropland	AU	↓						↑				**ARG diversity**: HT-qPCR array
[218]	Cropland	AU+PK	↑						↑				pH: ↓;<br>**ARG diversity**: HT-qPCR array
[219]	Cropland	AU+PK	↓						↑				pH: ↓;<br>**ARG diversity**: HT-qPCR array
[220]	Greenhous e soils	AU+PK							↑				**ARG diversity**: metagenomic sequencing and Structured ARG(SARG) database[221]
[222]	Cropland	pH↓							↓				105 cropland soil samples of China;<br>**ARG diversity**: HT-qPCR array
[223]	Forest	pH↓	↓						↓				**ARG diversity**: HT-qPCR array
[224]	Marine	Total N								↑			**GC content**: dividing the total amount of G and C bases by the total gene length
[47]	Meta-analysis	pH								↓	↑		**Genome size**: MicrobeCensus[57];<br>**GC content**: calculating by using the read depth for each contig
[225]	Forests, grasslands, shrublands	Soil total N↑ / pH									ns / ns		237 locations across the globe;<br>**Genome size**: surveying in the bacteria trait database
[40]	Global topsoils	pH↓						↓			↑		128 global soil sample datas;<br>***rrn* copy number**: Pereira-Flores et al[54];<br>**Genome size**: MicrobeCensus[57]
[226]	Forest	AN										↓	**Functional diversity**: GeoChip
[227]	Cropland	AU	ns									ns	**Functional diversity**: GeoChip
[228]	Cropland	AU+rice straw	ns									↑	pH: ↑;<br>**Functional diversity**: GeoChip
[229]	Meta-analysis	NPK	ns	ns								↑	pH: ↓;<br>**Functional diversity**: derived from Biolog

													Ecoplate™ measures of carbon substrate metabolism[230]
[231]	Cropland	NPK(AU)	ns	ns								↑	pH: ↓; **Functional diversity**: derived from Biolog Ecoplate™ measures of carbon substrate metabolism[230]
[232]	Greenhouse vegetable land	AU										↓	pH: ↓; **Functional diversity**: derived from Biolog Ecoplate™ measures of carbon substrate metabolism[230]
[233]	Apple orchard	AU										ns	pH: ↓; **Functional diversity**: derived from Biolog Ecoplate™ measures of carbon substrate metabolism[230]
[234]	Farmland	AU										↓	pH: ↓; **Functional diversity**: derived from Biolog Ecoplate™ measures of carbon substrate metabolism[230]
[235]	Cropland	AU										↑	**Functional diversity**: derived from Biolog Ecoplate™ measures of carbon substrate metabolism[230]
[236]	Cropland	AU										↓ (<20 g m^{-2} yr^{-1})	pH: ↓; **Functional diversity**: derived from Biolog Ecoplate™ measures of carbon substrate metabolism[230]
[237]	Grassland	AN										↓	pH: ↓; **Functional diversity**: derived from Biolog Ecoplate™ measures of

													carbon substrate metabolism[230]
[238]	Cropland	AU										ns	**Functional diversity**: derived from Biolog Ecoplate™ measures of carbon substrate metabolism[230]
[239]	Greenhouse	AU										↓	pH: ↓; **Functional diversity**: derived from Biolog Ecoplate™ measures of carbon substrate metabolism[230]
[240]	Grassland	AN / AN+SP	↓ / ↓									ns / ↑	pH: ↓
[241]	Global topsoils	pH↓	↓	ns								↓ (Bac) ↑ (Fun)	1,450 sites worldwide
[242]	Tibetan plateau	pH↓	ns									↑	
[243]	Grassland	pH↓	ns									↑	Loess Plateau
[244]	Eucalyptus plantation	pH↓										↑	**Functional diversity**: derived from Biolog Ecoplate™ measures of carbon substrate metabolism[230]
[245]	Mt. Norikura	pH↓	↓									↑	

AN, ammonium nitrate; AU, urea; ANU, urea-ammonium-nitrate; N, nitrogen fertilizer; AS, ammonium sulfate; SP, superphosphate; MP, monopotassium phosphate; P, phosphorus fertilizer; K, potassium fertilizer; G, glucose; RA, relative abundance; ↓, decrease; ↑, increase; ↓^{2}, decrease more; ↑^{2}, increase more; S.Bacteria, bacterial diversity; S.Fungi, fungal diversity; S.Phage, phage diversity; S.ARG, the diversity of bacterial antibiotic resistance genes; *rrn*, the average 16S rRNA gene copy number

Supplementary References

1. Yang GJ, Stevens C, Zhang ZJ, Lü XT, Han XG. Different nitrogen saturation thresholds for above-, below-, and total net primary productivity in a temperate steppe. *Global Change Biology* 2023;**29**:4586-94. https://doi.org/10.1111/gcb.16803
2. Yang GJ, Hautier Y, Zhang ZJ, Lü XT, Han XG. Decoupled responses of above-and below-ground stability of productivity to nitrogen addition at the local and larger spatial scale. *Global Change Biology* 2022;**28**:2711-20. https://doi.org/10.1111/gcb.16090
3. Caporaso JG, Kuczynski J, Stombaugh J, Bittinger K, Bushman FD, Costello EK *et al.* QIIME allows analysis of high-throughput community sequencing data. *Nature methods* 2010;**7**:335-36. https://doi.org/10.1038/nmeth.f.303
4. Bengtsson-Palme J, Ryberg M, Hartmann M, Branco S, Wang Z, Godhe A *et al.* Improved software detection and extraction of ITS1 and ITS2 from ribosomal ITS sequences of fungi and other eukaryotes for analysis of environmental sequencing data. *Methods of Ecology & Evolution* 2013;**4**:914-19. https://doi.org/10.1111/2041-210X.12073
5. Edgar RC. Search and clustering orders of magnitude faster than BLAST. *Bioinformatics* 2010;**26**:2460-61. https://doi.org/10.1093/bioinformatics/btq461
6. Schloss PD, Westcott SL, Ryabin T, Hall JR, Hartmann M, Hollister EB *et al.* Introducing mothur: Open-source, platform-independent, community-supported software for describing and comparing microbial communities. *Applied Environmental Microbiology* 2009;**75**:7537-41. https://doi.org/10.1128/AEM.01541-09
7. West PT, Probst AJ, Grigoriev IV, Thomas BC, Banfield JF. Genome-reconstruction for eukaryotes from complex natural microbial communities. *Genome Research* 2018;**28**:569-80. https://doi.org/10.1101/gr.228429.117
8. Guo J, Bolduc B, Zayed AA, Varsani A, Dominguez-Huerta G, Delmont TO *et al.* VirSorter2: a multi-classifier, expert-guided approach to detect diverse DNA and RNA viruses. *Microbiome* 2021;**9**:1-13. https://doi.org/10.1186/s40168-020-00990-y

9. Hyatt D, Chen GL, LoCascio PF, Land ML, Larimer FW, Hauser LJ. Prodigal: prokaryotic gene recognition and translation initiation site identification. *BMC bioinformatics* 2010;**11**:1-11. https://doi.org/10.1186/1471-2105-11-119

10. Bushnell B. BBMap: a fast, accurate, splice-aware aligner. *Lawrence Berkeley National Laboratory* 2014;LBNL-7065E. https://escholarship.org/uc/item/1h3515gn

11. Mistry J, Bateman A, Finn RD. Predicting active site residue annotations in the Pfam database. *BMC bioinformatics* 2007;**8**:1-14. https://doi.org/10.1186/1471-2105-8-298

12. Nayfach S, Camargo AP, Schulz F, Eloe-Fadrosh E, Roux S, Kyrpides NC. CheckV assesses the quality and completeness of metagenome-assembled viral genomes. *Nature biotechnology* 2021;**39**:578-85. https://doi.org/10.1038/s41587-020-00774-7

13. Menzel P, Ng KL, Krogh A. Fast and sensitive taxonomic classification for metagenomics with Kaiju. *Nature Communications* 2016;**7**:11257. https://doi.org/10.1038/ncomms11257

14. Oksanen J, Blanchet FG, Friendly M, Kindt R, Legendre P, McGlinn D *et al.* vegan: Community ecology package. R package version 2.5-7. https://CRAN.R-project.org/package=vegan

15. Gu Z, Gu L, Eils R, Schlesner M, Brors B. Circlize implements and enhances circular visualization in R. *Bioinformatics* 2014;**30**:2811-12.

16. Gezgin S, Bayrakll F. Ammonia volatilization from ammonium sulphate, ammonium nitrate, and urea surface applied to winter wheat on a calcareous soil. *Journal of plant nutrition* 1995;**18**:2483-94.

17. Keller GD, Mengel DB. Ammonia volatilization from nitrogen fertilizers surface applied to no-till corn. *Soil Science Society of America Journal* 1986;**50**:1060-63.

18. Bernardi AC, Mota EP, Cardosa RD, Monte MB, Oliveira PP. Ammonia volatilization from soil, dry-matter yield, and nitrogen levels of italian ryegrass. *Communications in soil science and plant analysis* 2014;**45**:153-62.

19. Chagas WFT, Guelfi DR, Caputo ALC, Souza TLd, Andrade AB, Faquin V. Ammonia volatilization from blends with stabilized and controlled-released urea in the coffee system. *Ciência e Agrotecnologia* 2016;**40**:497-509.
20. Knight EC, Guertal EA, Wood CW. Mowing and nitrogen source effects on ammonia volatilization from turfgrass. *Crop science* 2007;**47**:1628-34.
21. Fontoura SMV, Bayer C. Ammonia volatilization in no-till system in the south-central region of the state of paraná, brazil. *Revista Brasileira de Ciência do Solo* 2010;**34**:1677-84.
22. Faria LdA, Nascimento CACd, Vitti GC, Luz PHdC, Guedes EMS. Loss of ammonia from nitrogen fertilizers applied to maize and soybean straw. *Revista Brasileira de Ciência do Solo* 2013;**37**:969-75.
23. Lightner J, Mengel D, Rhykerd C. Ammonia volatilization from nitrogen fertilizer surface applied to orchardgrass sod. *Soil Science Society of America Journal* 1990;**54**:1478-82.
24. Massey CG, Slaton NA, Norman RJ, Gbur Jr EE, DeLong RE, Golden BR. Bermudagrass forage yield and ammonia volatilization as affected by nitrogen fertilization. *Soil Science Society of America Journal* 2011;**75**:638-48.
25. Oberle S, Bundy L. Ammonia volatilization from nitrogen fertilizers surface-applied to corn (zea mays) and grass pasture (dactylis glomerata). *Biology and Fertility of Soils* 1987;**4**:185-92.
26. Crusciol CAC, Almeida DS, Alves CJ, Soratto RP, Krebsky EO, Spolidorio ES. Mitigation of ammonia volatilisation from urea with micronised sulfur applied to common bean. *Soil Research* 2019;**57**:357-64. https://doi.org/https://doi.org/10.1071/SR18232
27. Corrêa DCdC, Cardoso AdS, Ferreira MR, Siniscalchi D, Gonçalves PHdA, Lumasini RN *et al.* Ammonia volatilization, forage accumulation, and nutritive value of marandu palisade grass pastures in different n sources and doses. *Atmosphere* 2021;**12**:1179.
28. Cassimiro JB, de Oliveira CLB, Boni AdS, Donato NdL, Meirelles GC, da Silva JF *et al.* Ammonia volatilization and marandu grass production in response to enhanced-efficiency nitrogen fertilizers. *Agronomy* 2023;**13**:837.

29. Souza TLd, Guelfi DR, Silva AL, Andrade AB, Chagas WFT, Cancellier EL. Ammonia and carbon dioxide emissions by stabilized conventional nitrogen fertilizers and controlled release in corn crop. *Ciência e Agrotecnologia* 2017;**41**:494-510.

30. Dominghetti AW, Guelfi DR, Guimarães RJ, Caputo ALC, Spehar CR, Faquin V. Nitrogen loss by volatilization of nitrogen fertilizers applied to coffee orchard. *Ciência e Agrotecnologia* 2016;**40**:173-83.

31. Freitas T, Bartelega L, Santos C, Dutra MP, Sarkis LF, Guimarães RJ *et al.* Technologies for fertilizers and management strategies of n-fertilization in coffee cropping systems to reduce ammonia losses by volatilization. *Plants* 2022;**11**:3323.

32. Barth G, Otto R, Mira AB, Ferraz-Almeida R, Vitti AC, Cantarella H *et al.* Performance of enhanced efficiency nitrogen fertilizers in green-harvesting sugarcane. *Agrosystems, Geosciences & Environment* 2020;**3**:e20015.

33. Santos C, Pinto SIdC, Guelfi D, Rosa SD, da Fonseca AB, Fernandes TJ *et al.* Corn cropping system and nitrogen fertilizers technologies affect ammonia volatilization in brazilian tropical soils. *Soil Systems* 2023;**7**:54.

34. Malik AA, Martiny JB, Brodie EL, Martiny AC, Treseder KK, Allison SD. Defining trait-based microbial strategies with consequences for soil carbon cycling under climate change. *The ISME journal* 2020;**14**:1-9.

35. Yang L, Canarini A, Zhang W, Lang M, Chen Y, Cui Z *et al.* Microbial life-history strategies mediate microbial carbon pump efficacy in response to n management depending on stoichiometry of microbial demand. *Global Change Biology* 2024;**30**:e17311.

36. Li C, Liao H, Xu L, Wang C, He N, Wang J *et al.* The adjustment of life history strategies drives the ecological adaptations of soil microbiota to aridity. *Molecular ecology* 2022;**31**:2920-34.

37. Bittleston LS, Freedman ZB, Bernardin JR, Grothjan JJ, Young EB, Record S *et al.* Exploring microbiome functional dynamics through space and time with trait-based theory. *Msystems* 2021;**6**:10.1128/msystems. 00530-21.

38. Sun Y, Tao C, Deng X, Liu H, Shen Z, Liu Y *et al.* Organic fertilization enhances the resistance and resilience of soil microbial communities under extreme drought. *Journal of Advanced Research* 2023;**47**:1-12.

39. Finn D, Yu J, Penton CR. Soil quality shapes the composition of microbial community stress response and core cell metabolism functional genes. *Applied Soil Ecology* 2020;**148**:103483.

40. Piton G, Allison SD, Bahram M, Hildebrand F, Martiny JB, Treseder KK *et al.* Life history strategies of soil bacterial communities across global terrestrial biomes. *Nature microbiology* 2023;**8**:2093-102.

41. Cania B, Vestergaard G, Suhadolc M, Mihelič R, Krauss M, Fliessbach A *et al.* Site-specific conditions change the response of bacterial producers of soil structure-stabilizing agents such as exopolysaccharides and lipopolysaccharides to tillage intensity. *Frontiers in microbiology* 2020;**11**:568.

42. Chávez J, Devos DP, Merino E. Complementary tendencies in the use of regulatory elements (transcription factors, sigma factors, and riboswitches) in bacteria and archaea. *Journal of Bacteriology* 2020;**203**:10.1128/jb. 00413-20.

43. Sharma MP, Grover M, Chourasiya D, Bharti A, Agnihotri R, Maheshwari HS *et al.* Deciphering the role of trehalose in tripartite symbiosis among rhizobia, arbuscular mycorrhizal fungi, and legumes for enhancing abiotic stress tolerance in crop plants. *Frontiers in Microbiology* 2020;**11** https://doi.org/10.3389/fmicb.2020.509919

44. Sorouri B, Rodriguez CI, Gaut BS, Allison SD. Variation in sphingomonas traits across habitats and phylogenetic clades. *Frontiers in Microbiology* 2023;**14**:1146165.

45. Ning Q, Chen L, Li F, Zhou G, Zhang C, Ma D *et al.* Tradeoffs of microbial life history strategies drive the turnover of microbial-derived organic carbon in coastal saline soils. *Frontiers in Microbiology* 2023;**14**:1141436.

46. Bei Q, Moser G, Müller C, Liesack W. Seasonality affects function and complexity but not diversity of the rhizosphere microbiome in european temperate grassland. *Science of The Total Environment* 2021;**784**:147036.

47. Chuckran PF, Flagg C, Propster J, Rutherford WA, Sieradzki ET, Blazewicz SJ *et al.* Edaphic controls on genome size and gc content of bacteria in soil microbial communities. *Soil Biology and Biochemistry* 2023;**178**:108935.

48. Reji L, Darnajoux R, Zhang X. A genomic view of environmental and life history controls on microbial nitrogen acquisition strategies. *Environmental Microbiology Reports* 2024;**16**:e13220.

49. Osburn ED, McBride SG, Bahram M, Strickland MS. Global patterns in the growth potential of soil bacterial communities. *Nature Communications* 2024;**15**:6881.

50. Avalos M, Garbeva P, Vader L, van Wezel GP, Dickschat JS, Ulanova D. Biosynthesis, evolution and ecology of microbial terpenoids. *Natural Product Reports* 2022;**39**:249-72.

51. Fuchs G, Boll M, Heider J. Microbial degradation of aromatic compounds — from one strategy to four. *Nature Reviews Microbiology* 2011;**9**:803-16. https://doi.org/10.1038/nrmicro2652

52. Ni B, Colin R, Link H, Endres RG, Sourjik V. Growth-rate dependent resource investment in bacterial motile behavior quantitatively follows potential benefit of chemotaxis. *Proceedings of the National Academy of Sciences* 2020;**117**:595-601. https://doi.org/doi:10.1073/pnas.1910849117

53. Weissman JL, Hou S, Fuhrman JA. Estimating maximal microbial growth rates from cultures, metagenomes, and single cells via codon usage patterns. *Proceedings of the National Academy of Sciences* 2021;**118**:e2016810118. https://doi.org/10.1073/pnas.2016810118

54. Pereira-Flores E, Glöckner FO, Fernandez-Guerra A. Fast and accurate average genome size and 16s rrna gene average copy number computation in metagenomic data. *BMC Bioinformatics* 2019;**20**:453. https://doi.org/10.1186/s12859-019-3031-y

55. Gurevich A, Saveliev V, Vyahhi N, Tesler G. Quast: Quality assessment tool for genome assemblies. *Bioinformatics* 2013;**29**:1072-75.

56. Gibson MK, Forsberg KJ, Dantas G. Improved annotation of antibiotic resistance determinants reveals microbial resistomes cluster by ecology. *The ISME journal* 2015;**9**:207-16.

57. Nayfach S, Pollard KS. Average genome size estimation improves comparative metagenomics and sheds light on the functional ecology of the human microbiome. *Genome Biology* 2015;**16**:51. https://doi.org/10.1186/s13059-015-0611-7
58. Wang C, Yu Q-Y, Ji N-N, Zheng Y, Taylor JW, Guo L-D *et al.* Bacterial genome size and gene functional diversity negatively correlate with taxonomic diversity along a ph gradient. *Nature Communications* 2023;**14**:7437. https://doi.org/10.1038/s41467-023-43297-w
59. Yang J-X, Peng Y, Yu Q-Y, Yang J-J, Zhang Y-H, Zhang H-Y *et al.* Gene horizontal transfers and functional diversity negatively correlated with bacterial taxonomic diversity along a nitrogen gradient. *npj Biofilms and Microbiomes* 2024;**10**:128. https://doi.org/10.1038/s41522-024-00588-4
60. Leff JW, Jones SE, Prober SM, Barberán A, Borer ET, Firn JL *et al.* Consistent responses of soil microbial communities to elevated nutrient inputs in grasslands across the globe. *Proceedings of the National Academy of Sciences* 2015;**112**:10967-72.
61. Ling N, Chen D, Guo H, Wei J, Bai Y, Shen Q *et al.* Differential responses of soil bacterial communities to long-term n and p inputs in a semi-arid steppe. *Geoderma* 2017;**292**:25-33.
62. Zhou J, Guan D, Zhou B, Zhao B, Ma M, Qin J *et al.* Influence of 34-years of fertilization on bacterial communities in an intensively cultivated black soil in northeast china. *Soil Biology and Biochemistry* 2015;**90**:42-51.
63. Zeng J, Liu X, Song L, Lin X, Zhang H, Shen C *et al.* Nitrogen fertilization directly affects soil bacterial diversity and indirectly affects bacterial community composition. *Soil Biology and Biochemistry* 2016;**92**:41-49. https://doi.org/https://doi.org/10.1016/j.soilbio.2015.09.018
64. Li B-B, Roley SS, Duncan DS, Guo J, Quensen JF, Yu H-Q *et al.* Long-term excess nitrogen fertilizer increases sensitivity of soil microbial community to seasonal change revealed by ecological network and metagenome analyses. *Soil Biology and Biochemistry* 2021;**160**:108349. https://doi.org/https://doi.org/10.1016/j.soilbio.2021.108349

65. Dai Z, Su W, Chen H, Barberán A, Zhao H, Yu M *et al.* Long-term nitrogen fertilization decreases bacterial diversity and favors the growth of actinobacteria and proteobacteria in agro-ecosystems across the globe. *Global change biology* 2018;**24**:3452-61.

66. Yang Y, Chen X, Liu L, Li T, Dou Y, Qiao J *et al.* Nitrogen fertilization weakens the linkage between soil carbon and microbial diversity: A global meta-analysis. *Global Change Biology* 2022;**28**:6446-61. https://doi.org/https://doi.org/10.1111/gcb.16361

67. Cui J, Yuan X, Zhang Q, Zhou J, Lin K, Xu J *et al.* Nutrient availability is a dominant predictor of soil bacterial and fungal community composition after nitrogen addition in subtropical acidic forests. *PLOS ONE* 2021;**16**:e0246263. https://doi.org/10.1371/journal.pone.0246263

68. Zhou Z, Wang C, Luo Y. Meta-analysis of the impacts of global change factors on soil microbial diversity and functionality. *Nature Communications* 2020;**11**:3072. https://doi.org/10.1038/s41467-020-16881-7

69. Wang C, Shi Z, Li A, Geng T, Liu L, Liu W. Long-term nitrogen input reduces soil bacterial network complexity by shifts in life history strategy in temperate grassland. *iMeta* 2024;**3**:e194. https://doi.org/https://doi.org/10.1002/imt2.194

70. Liu W, Ling N, Guo J, Ruan Y, Zhu C, Shen Q *et al.* Legacy effects of 8-year nitrogen inputs on bacterial assemblage in wheat rhizosphere. *Biology and Fertility of Soils* 2020;**56**:583-96.

71. Wang H, Liu S, Zhang X, Mao Q, Li X, You Y *et al.* Nitrogen addition reduces soil bacterial richness, while phosphorus addition alters community composition in an old-growth n-rich tropical forest in southern china. *Soil Biology and Biochemistry* 2018;**127**:22-30.

72. Wang C, Liu D, Bai E. Decreasing soil microbial diversity is associated with decreasing microbial biomass under nitrogen addition. *Soil Biology and Biochemistry* 2018;**120**:126-33. https://doi.org/https://doi.org/10.1016/j.soilbio.2018.02.003

73. Liu W, Jiang L, Yang S, Wang Z, Tian R, Peng Z *et al.* Critical transition of soil bacterial diversity and composition triggered by nitrogen enrichment. *Ecology* 2020;**101**:e03053.

74. Wang Q, Wang C, Yu W, Turak A, Chen D, Huang Y *et al.* Effects of nitrogen and phosphorus inputs on soil bacterial abundance, diversity, and community composition in chinese fir plantations. *Frontiers in microbiology* 2018;**9**:1543.

75. He J, Tan X, Nie Y, Ma L, Liu J, Lu X *et al.* Distinct responses of abundant and rare soil bacteria to nitrogen addition in tropical forest soils. *Microbiology Spectrum* 2023;**11**:e03003-22.

76. Zhou J, Jiang X, Wei D, Zhao B, Ma M, Chen S *et al.* Consistent effects of nitrogen fertilization on soil bacterial communities in black soils for two crop seasons in china. *Scientific reports* 2017;**7**:3267.

77. Song B, Li Y, Yang L, Shi H, Li L, Bai W *et al.* Soil acidification under long-term n addition decreases the diversity of soil bacteria and fungi and changes their community composition in a semiarid grassland. *Microbial ecology* 2023;**85**:221-31.

78. Jackson CR, Vallaire SC. Effects of salinity and nutrients on microbial assemblages in louisiana wetland sediments. *Wetlands* 2009;**29**:277-87.

79. Li D, Meng M, Ren BH, Ma X, Bai L, Li J. Different responses of soil fungal and bacterial communities to nitrogen addition in a forest grassland ecotone. *Frontiers in Microbiology* 2023;**14**:1211768.

80. Liu W, Liu L, Yang X, Deng M, Wang Z, Wang P *et al.* Long-term nitrogen input alters plant and soil bacterial, but not fungal beta diversity in a semiarid grassland. *Global Change Biology* 2021;**27**:3939-50.

81. Zhalnina K, Dias R, de Quadros PD, Davis-Richardson A, Camargo FA, Clark IM *et al.* Soil ph determines microbial diversity and composition in the park grass experiment. *Microbial ecology* 2015;**69**:395-406.

82. Zhang X, Wei H, Chen Q, Han X. The counteractive effects of nitrogen addition and watering on soil bacterial communities in a steppe ecosystem. *Soil Biology and Biochemistry* 2014;**72**:26-34.

83. Mueller RC, Belnap J, Kuske CR. Soil bacterial and fungal community responses to nitrogen addition across soil depth and microhabitat in an arid shrubland. *Frontiers in microbiology* 2015;**6**:157127.

84. Fierer N, Lauber CL, Ramirez KS, Zaneveld J, Bradford MA, Knight R. Comparative metagenomic, phylogenetic and physiological analyses of soil microbial communities across nitrogen gradients. *The ISME journal* 2012;**6**:1007-17.

85. Zhang X, Han X. Nitrogen deposition alters soil chemical properties and bacterial communities in the inner mongolia grassland. *Journal of Environmental Sciences* 2012;**24**:1483-91.

86. Chen M, Zheng Y, Zhai X, Ma F, Chen J, Stevens C *et al.* Metal ions steer the duality in microbial community recovery from nitrogen enrichment by shaping functional groups. *Global Change Biology* 2024;**30**:e17475. https://doi.org/https://doi.org/10.1111/gcb.17475

87. Coolon JD, Jones KL, Todd TC, Blair JM, Herman MA. Long-term nitrogen amendment alters the diversity and assemblage of soil bacterial communities in tallgrass prairie. *PLoS One* 2013;**8**:e67884.

88. Cui J, Wang J, Xu J, Xu C, Xu X. Changes in soil bacterial communities in an evergreen broad-leaved forest in east china following 4 years of nitrogen addition. *Journal of Soils and Sediments* 2017;**17**:2156-64.

89. Kavamura VN, Hayat R, Clark IM, Rossmann M, Mendes R, Hirsch PR *et al.* Inorganic nitrogen application affects both taxonomical and predicted functional structure of wheat rhizosphere bacterial communities. *Frontiers in microbiology* 2018;**9**:378965.

90. Aßhauer KP, Wemheuer B, Daniel R, Meinicke P. Tax4fun: Predicting functional profiles from metagenomic 16s rrna data. *Bioinformatics* 2015;**31**:2882-84.

91. Wang J, Liao L, Ye Z, Liu H, Zhang C, Zhang L *et al.* Different bacterial co-occurrence patterns and community assembly between rhizosphere and bulk soils under n addition in the plant–soil system. *Plant and Soil* 2022:1-17.

92. Zhong Y, Yan W, Shangguan Z. Impact of long-term n additions upon coupling between soil microbial community structure and activity, and nutrient-use efficiencies. *Soil Biology and Biochemistry* 2015;**91**:151-59.

93. Chen X, Song H, Sun B, Yang T. Effects of simulated atmospheric nitrogen deposition on the bacterial community structure and diversity of four distinct biocolonization types on stone monuments: A case study of the leshan giant buddha, a world heritage site. *Heritage Science* 2024;**12**:23.

94. Zhao Z-B, He J-Z, Geisen S, Han L-L, Wang J-T, Shen J-P *et al.* Protist communities are more sensitive to nitrogen fertilization than other microorganisms in diverse agricultural soils. *Microbiome* 2019;**7**:1-16.

95. Fu X, Dai Y, Cui J, Deng P, Fan W, Xu X. Soil bacterial and fungal communities resilience to long-term nitrogen addition in subtropical forests in china. *Journal of Forestry Research* 2024;**35**:17.

96. Ma X, Song Y, Song C, Wang X, Wang N, Gao S *et al.* Effect of nitrogen addition on soil microbial functional gene abundance and community diversity in permafrost peatland. *Microorganisms* 2021;**9**:2498.

97. Ramirez KS, Lauber CL, Knight R, Bradford MA, Fierer N. Consistent effects of nitrogen fertilization on soil bacterial communities in contrasting systems. *Ecology* 2010;**91**:3463-70.

98. Li J, Yang C, Zhou H, Shao X. Responses of plant diversity and soil microorganism diversity to water and nitrogen additions in the qinghai-tibetan plateau. *Global Ecology and Conservation* 2020;**22**:e01003.

99. Li Y, Tremblay J, Bainard LD, Cade-Menun B, Hamel C. Long-term effects of nitrogen and phosphorus fertilization on soil microbial community structure and function under continuous wheat production. *Environmental Microbiology* 2020;**22**:1066-88.

100. Wu J, Liu W, Zhang W, Shao Y, Duan H, Chen B *et al.* Long-term nitrogen addition changes soil microbial community and litter decomposition rate in a subtropical forest. *Applied Soil Ecology* 2019;**142**:43-51.

101. Ullah S, He P, Ai C, Zhao S, Ding W, Song D *et al.* How do soil bacterial diversity and community composition respond under recommended and conventional nitrogen fertilization regimes? *Microorganisms* 2020;**8**:1193.

102. Wang Z, Na R, Koziol L, Schellenberg MP, Li X, Ta N *et al.* Response of bacterial communities and plant-mediated soil processes to nitrogen deposition and precipitation in a desert steppe. *Plant and Soil* 2020;**448**:277-97.

103. Craig H, Antwis RE, Cordero I, Ashworth D, Robinson CH, Osborne TZ *et al.* Nitrogen addition alters composition, diversity, and functioning of microbial communities in mangrove soils: An incubation experiment. *Soil Biology and Biochemistry* 2021;**153**:108076.

104. Yang Y, Cheng H, Gao H, An S. Response and driving factors of soil microbial diversity related to global nitrogen addition. *Land Degradation & Development* 2020;**31**:190-204.

105. Fu Y, Yan G, Liu G, Huang B, Sun X, Wang X *et al.* Responses of the rhizosphere microbiome to long-term nitrogen addition in a boreal forest. *Canadian Journal of Forest Research* 2022;**52**:1071-87.

106. Castellano-Hinojosa A, Strauss SL, González-López J, Bedmar EJ. Changes in the diversity and predicted functional composition of the bulk and rhizosphere soil bacterial microbiomes of tomato and common bean after inorganic n-fertilization. *Rhizosphere* 2021;**18**:100362.

107. Dou W, Xiao B, Delgado-Baquerizo M, Revillini D, Kidron GJ. Dryland nitrogen deposition induces microbiome-driven increases in biocrust respiration and losses of soil carbon. *Land Degradation & Development* 2024;**35**:647-58. https://doi.org/https://doi.org/10.1002/ldr.4942

108. Yun Y, Wang H, Man B, Xiang X, Zhou J, Qiu X *et al.* The relationship between ph and bacterial communities in a single karst ecosystem and its implication for soil acidification. *Frontiers in Microbiology* 2016;**7**:213262.

109. Wan W, Tan J, Wang Y, Qin Y, He H, Wu H *et al.* Responses of the rhizosphere bacterial community in acidic crop soil to ph: Changes in diversity, composition, interaction, and function. *Science of the Total Environment* 2020;**700**:134418.

110. Du Y, Wang T, Wang C, Anane P-S, Liu S, Paz-Ferreiro J. Nitrogen fertilizer is a key factor affecting the soil chemical and microbial communities in a mollisol. *Canadian journal of microbiology* 2019;**65**:510-21.

111. Zhao S, Liu J-J, Banerjee S, Zhou N, Zhao Z-Y, Zhang K *et al.* Soil ph is equally important as salinity in shaping bacterial communities in saline soils under halophytic vegetation. *Scientific reports* 2018;**8**:4550.

112. Fierer N, Jackson RB. The diversity and biogeography of soil bacterial communities. *Proceedings of the National Academy of Sciences* 2006;**103**:626-31.

113. Hartman WH, Richardson CJ, Vilgalys R, Bruland GL. Environmental and anthropogenic controls over bacterial communities in wetland soils. *Proceedings of the national academy of sciences* 2008;**105**:17842-47.

114. Fierer N, Leff JW, Adams BJ, Nielsen UN, Bates ST, Lauber CL *et al.* Cross-biome metagenomic analyses of soil microbial communities and their functional attributes. *Proceedings of the National Academy of Sciences* 2012;**109**:21390-95.

115. Delgado-Baquerizo M, Eldridge DJ. Cross-biome drivers of soil bacterial alpha diversity on a worldwide scale. *Ecosystems* 2019;**22**:1220-31.

116. Thompson LR, Sanders JG, McDonald D, Amir A, Ladau J, Locey KJ *et al.* A communal catalogue reveals earth's multiscale microbial diversity. *Nature* 2017;**551**:457-63.

117. Guerra CA, Berdugo M, Eldridge DJ, Eisenhauer N, Singh BK, Cui H *et al.* Global hotspots for soil nature conservation. *Nature* 2022;**610**:693-98.

118. Griffiths RI, Thomson BC, James P, Bell T, Bailey M, Whiteley AS. The bacterial biogeography of british soils. *Environmental microbiology* 2011;**13**:1642-54.

119. Rousk J, Bååth E, Brookes PC, Lauber CL, Lozupone C, Caporaso JG *et al.* Soil bacterial and fungal communities across a ph gradient in an arable soil. *The ISME journal* 2010;**4**:1340-51.

120. Lauber CL, Hamady M, Knight R, Fierer N. Pyrosequencing-based assessment of soil ph as a predictor of soil bacterial community structure at the continental scale. *Applied and environmental microbiology* 2009;**75**:5111-20.

121. Liu S, Ren H, Shen L, Lou L, Tian G, Zheng P *et al.* Ph levels drive bacterial community structure in sediments of the qiantang river as determined by 454 pyrosequencing. *Frontiers in Microbiology* 2015;**6** https://doi.org/10.3389/fmicb.2015.00285

122. Liu J, Sui Y, Yu Z, Shi Y, Chu H, Jin J *et al.* High throughput sequencing analysis of biogeographical distribution of bacterial communities in the black soils of northeast china. *Soil Biology and Biochemistry* 2014;**70**:113-22.

123. Yan K, Dong Y, Gong Y, Zhu Q, Wang Y. Climatic and edaphic factors affecting soil bacterial community biodiversity in different forests of china. *Catena* 2021;**207**:105675.

124. Ni Y, Yang T, Ma Y, Zhang K, Soltis PS, Soltis DE *et al.* Soil ph determines bacterial distribution and assembly processes in natural mountain forests of eastern china. *Global Ecology and Biogeography* 2021;**30**:2164-77.

125. Xiang J, Gu J, Wang G, Bol R, Yao L, Fang Y *et al.* Soil ph controls the structure and diversity of bacterial communities along elevational gradients on huangshan, china. *European Journal of Soil Biology* 2024;**120**:103586. https://doi.org/https://doi.org/10.1016/j.ejsobi.2023.103586

126. Li H-Q, Shen Y-J, Wang W-L, Wang H-T, Li H, Su J-Q. Soil ph has a stronger effect than arsenic content on shaping plastisphere bacterial communities in soil. *Environmental Pollution* 2021;**287**:117339.

127. Yavitt JB, Roco CA, Debenport SJ, Barnett SE, Shapleigh JP. Community organization and metagenomics of bacterial assemblages across local scale ph gradients in northern forest soils. *Microbial ecology* 2021;**81**:758-69.

128. Kaiser K, Wemheuer B, Korolkow V, Wemheuer F, Nacke H, Schöning I *et al.* Driving forces of soil bacterial community structure, diversity, and function in temperate grasslands and forests. *Scientific Reports* 2016;**6**:33696.

129. Tan W, Wang J, Bai W, Qi J, Chen W. Soil bacterial diversity correlates with precipitation and soil ph in long-term maize cropping systems. *Scientific reports* 2020;**10**:6012.

130. Duan Y, Liu Q, Wang Y, Zhang J, Xiong D. Impairment of the intestine barrier function in litopenaeus vannamei exposed to ammonia and nitrite stress. *Fish & shellfish immunology* 2018;**78**:279-88.

131. Song M, Sun B, Li R, Qian Z, Bai Z, Zhuang X. Successions and interactions of phyllospheric microbiome in response to nh3 exposure. *Science of The Total Environment* 2022;**837**:155805.

132. Kalamaras SD, Vasileiadis S, Karas P, Angelidaki I, Kotsopoulos TA. Microbial adaptation to high ammonia concentrations during anaerobic digestion of manure-based feedstock: Biomethanation and 16s rrna gene sequencing. *Journal of Chemical Technology & Biotechnology* 2020;**95**:1970-79.

133. Gao M, Guo B, Zhang L, Zhang Y, Liu Y. Microbial community dynamics in anaerobic digesters treating conventional and vacuum toilet flushed blackwater. *Water Research* 2019;**160**:249-58.

134. Sun S, Qiao Z, Sun K, Huo D. Assembly process and co-occurrence network of microbial community in response to free ammonia gradient distribution. *Microbiology Spectrum* 2024;**12**:e01051-24. https://doi.org/doi:10.1128/spectrum.01051-24

135. Zhang J, Buhe C, Yu D, Zhong H, Wei Y. Ammonia stress reduces antibiotic efflux but enriches horizontal gene transfer of antibiotic resistance genes in anaerobic digestion. *Bioresource Technology* 2020;**295**:122191.

136. Chen Y, Xu Z, Feng K, Yang G, Fu W, Chen B. Nitrogen and water addition regulate soil fungal diversity and co-occurrence networks. *Journal of Soils and Sediments* 2020;**20**:3192-203. https://doi.org/10.1007/s11368-020-02629-9

137. Xu J, Wang Y, Zhang Y, Li Q, Du B, Asitaiken JLHT *et al.* Effect of nitrogen addition on soil net nitrogen mineralization in topsoil and subsoil regulated by soil microbial properties and mineral protection: Evidence from a long-term grassland experiment. *Science of The Total Environment* 2024;**947**:174686. https://doi.org/https://doi.org/10.1016/j.scitotenv.2024.174686

138. Wang Y, Zhang H, Yu G, Dong L, Kang J, Wang W. Grassland degradation affects the response of soil bacterial and plant but not fungal diversity to nitrogen addition. *Journal of Ecology* 2023;**111**:2269-80. https://doi.org/https://doi.org/10.1111/1365-2745.14179

139. Liao L, Wang X, Wang J, Liu G, Zhang C. Nitrogen fertilization increases fungal diversity and abundance of saprotrophs while reducing nitrogen fixation potential in a semiarid grassland. *Plant and Soil* 2021;**465**:515-32. https://doi.org/10.1007/s11104-021-05012-w

140. Yang H, Cheng L, Che L, Su Y, Li Y. Nutrients addition decreases soil fungal diversity and alters fungal guilds and co-occurrence networks in a semi-arid grassland in northern china. *Science of The Total Environment* 2024;**926**:172100. https://doi.org/https://doi.org/10.1016/j.scitotenv.2024.172100

141. Zhou J, Jiang X, Zhou B, Zhao B, Ma M, Guan D *et al.* Thirty four years of nitrogen fertilization decreases fungal diversity and alters fungal community composition in black soil in northeast china. *Soil Biology and Biochemistry* 2016;**95**:135-43. https://doi.org/https://doi.org/10.1016/j.soilbio.2015.12.012

142. Chen W, Xu R, Chen J, Yuan X, Zhou L, Tan T *et al.* Consistent responses of surface- and subsurface soil fungal diversity to n enrichment are mediated differently by acidification and plant community in a semi-arid grassland. *Soil Biology and Biochemistry* 2018;**127**:110-19. https://doi.org/https://doi.org/10.1016/j.soilbio.2018.09.020

143. Zhang H, Wang L, Liu H, Zhao J, Li G, Wang H *et al.* Nitrogen deposition combined with elevated precipitation is conducive to maintaining the stability of the soil fungal diversity on the stipa baicalensis steppe. *Soil Biology and Biochemistry* 2018;**117**:135-38. https://doi.org/https://doi.org/10.1016/j.soilbio.2017.11.004

144. He J, Jiao S, Tan X, Wei H, Ma X, Nie Y *et al.* Adaptation of soil fungal community structure and assembly to long- versus short-term nitrogen addition in a tropical forest. *Frontiers in Microbiology* 2021;**12** https://doi.org/10.3389/fmicb.2021.689674

145. He D, Xiang X, He J-S, Wang C, Cao G, Adams J *et al.* Composition of the soil fungal community is more sensitive to phosphorus than nitrogen addition in the alpine meadow on the qinghai-tibetan plateau. *Biology and Fertility of Soils* 2016;**52**:1059-72. https://doi.org/10.1007/s00374-016-1142-4

146. Chen W, Xu R, Wu Y, Chen J, Zhang Y, Hu T *et al.* Plant diversity is coupled with beta not alpha diversity of soil fungal communities following n enrichment in a semi-arid grassland. *Soil Biology and Biochemistry* 2018;**116**:388-98. https://doi.org/https://doi.org/10.1016/j.soilbio.2017.10.039

147. Yan Y, Sun X, Sun F, Zhao Y, Sun W, Guo J *et al.* Sensitivity of soil fungal and bacterial community compositions to nitrogen and phosphorus additions in a temperate meadow. *Plant and Soil* 2022;**471**:477-90. https://doi.org/10.1007/s11104-021-05237-9

148. Xiao Y, Li C, Yang Y, Peng Y, Yang Y, Zhou G. Soil fungal community composition, not assembly process, was altered by nitrogen addition and precipitation changes at an alpine steppe. *Frontiers in Microbiology* 2020;**11** https://doi.org/10.3389/fmicb.2020.579072

149. Zhan J, Li Y, Zhao X, Yang H, Ning Z, Zhang R. Effects of nitrogen addition and plant litter manipulation on soil fungal and bacterial communities in a semiarid sandy land. *Frontiers in Microbiology* 2023;**14** https://doi.org/10.3389/fmicb.2023.1013570

150. Zhang X, Song X, Wang T, Huang L, Ma H, Wang M *et al.* The responses to long-term nitrogen addition of soil bacterial, fungal, and archaeal communities in a desert ecosystem. *Frontiers in Microbiology* 2022;**13** https://doi.org/10.3389/fmicb.2022.1015588

151. Li Y, Tian D, Wang J, Niu S, Tian J, Ha D *et al.* Differential mechanisms underlying responses of soil bacterial and fungal communities to nitrogen and phosphorus inputs in a subtropical forest. *PeerJ* 2019;**7**:e7631. https://doi.org/10.7717/peerj.7631

152. Jiang M, Tian Y, Guo R, Li S, Guo J, Zhang T. Effects of warming and nitrogen addition on soil fungal and bacterial community structures in a temperate meadow. *Frontiers in Microbiology* 2023;**14** https://doi.org/10.3389/fmicb.2023.1231442

153. McHugh TA, Morrissey EM, Mueller RC, Gallegos-Graves LV, Kuske CR, Reed SC. Bacterial, fungal, and plant communities exhibit no biomass or compositional response to two years of simulated nitrogen deposition in a semiarid grassland. *Environmental Microbiology* 2017;**19**:1600-11. https://doi.org/https://doi.org/10.1111/1462-2920.13678

154. Cao M, Zheng X, Cui L, Wu F, Gao H, Jiang J. Soil bacterial communities are more sensitive to short-term nitrogen deposition than fungal communities in subtropical chinese fir forests. *Forest Ecology and Management* 2023;**549**:121490. https://doi.org/https://doi.org/10.1016/j.foreco.2023.121490

155. Zheng X, Xu A, Lin Y, Wang H, Ding H, Wu Y *et al.* Long-term nitrogen deposition alters the soil bacterial community structure but has little effect on fungal communities. *Global Ecology and Conservation* 2024;**54**:e03080. https://doi.org/https://doi.org/10.1016/j.gecco.2024.e03080

156. Wang X, Feng J, Ao G, Qin W, Han M, Shen Y *et al.* Globally nitrogen addition alters soil microbial community structure, but has minor effects on soil microbial diversity and richness. *Soil Biology and Biochemistry* 2023;**179**:108982. https://doi.org/https://doi.org/10.1016/j.soilbio.2023.108982

157. Xi D, Jin S, Wu J. Soil bacterial community is more sensitive than fungal community to canopy nitrogen deposition and understory removal in a chinese fir plantation. *Frontiers in Microbiology* 2022;**13** https://doi.org/10.3389/fmicb.2022.1015936

158. Yang N, Zhang J, Hua J, Song B, Wang T, Xing W *et al.* Differentiation of fungal trophic guilds to long-term nitrogen addition in a poplar plantation. *Forest Ecology and Management* 2024;**555**:121699. https://doi.org/https://doi.org/10.1016/j.foreco.2024.121699

159. Tedersoo L, Anslan S, Bahram M, Drenkhan R, Pritsch K, Buegger F *et al.* Regional-scale in-depth analysis of soil fungal diversity reveals strong ph and plant species effects in northern europe. *Frontiers in Microbiology* 2020;**11** https://doi.org/10.3389/fmicb.2020.01953

160. Liu D, Liu G, Chen L, Wang J, Zhang L. Soil ph determines fungal diversity along an elevation gradient in southwestern china. *Science China Life Sciences* 2018;**61**:718-26. https://doi.org/10.1007/s11427-017-9200-1

161. Wang J-T, Zheng Y-M, Hu H-W, Zhang L-M, Li J, He J-Z. Soil ph determines the alpha diversity but not beta diversity of soil fungal community along altitude in a typical tibetan forest ecosystem. *Journal of Soils and Sediments* 2015;**15**:1224-32. https://doi.org/10.1007/s11368-015-1070-1

162. Canini F, Geml J, D'Acqui LP, Selbmann L, Onofri S, Ventura S *et al.* Exchangeable cations and ph drive diversity and functionality of fungal communities in biological soil crusts from coastal sites of victoria land, antarctica. *Fungal Ecology* 2020;**45**:100923. https://doi.org/https://doi.org/10.1016/j.funeco.2020.100923

163. Tedersoo L, Bahram M, Põlme S, Kõljalg U, Yorou NS, Wijesundera R *et al.* Global diversity and geography of soil fungi. *Science* 2014;**346**:1256688. https://doi.org/doi:10.1126/science.1256688

164. Siles JA, Margesin R. Abundance and diversity of bacterial, archaeal, and fungal communities along an altitudinal gradient in alpine forest soils: What are the driving factors? *Microbial Ecology* 2016;**72**:207-20. https://doi.org/10.1007/s00248-016-0748-2

165. Jasrotia P, Green SJ, Canion A, Overholt WA, Prakash O, Wafula D *et al.* Watershed-scale fungal community characterization along a ph gradient in a subsurface environment cocontaminated with uranium and nitrate. *Applied and Environmental Microbiology* 2014;**80**:1810-20. https://doi.org/doi:10.1128/AEM.03423-13

166. Xiao D, He X, Zhang W, Hu P, Sun M, Wang K. Comparison of bacterial and fungal diversity and network connectivity in karst and non-karst forests in southwest china. *Science of The Total Environment* 2022;**822**:153179. https://doi.org/https://doi.org/10.1016/j.scitotenv.2022.153179

167. Zhou Y, Jia X, Han L, Liu Z, Kang S, Zhao Y. Fungal community diversity in soils along an elevation gradient in a quercus aliena var. Acuteserrata forest in qinling mountains, china. *Applied Soil Ecology* 2021;**167**:104104. https://doi.org/https://doi.org/10.1016/j.apsoil.2021.104104

168. Chen J, Shi Z, Liu S, Zhang M, Cao X, Chen M *et al.* Altitudinal variation influences soil fungal community composition and diversity in alpine–gorge region on the eastern qinghai–tibetan plateau. *Journal of Fungi* 2022;**8**:807.

169. Li J, Chen L, Wang H, Ouyang S, Liu X, Lu J. Pattern and drivers of soil fungal community along elevation gradient in the abies georgei forests of segila mountains, southeast tibet. *Global Ecology and Conservation* 2022;**39**:e02291. https://doi.org/https://doi.org/10.1016/j.gecco.2022.e02291

170. Gu S, Wu S, Zeng W, Deng Y, Luo G, Li P *et al.* High-elevation-induced decrease in soil ph weakens ecosystem multifunctionality by influencing soil microbiomes. *Environmental Research* 2024;**257**:119330. https://doi.org/https://doi.org/10.1016/j.envres.2024.119330

171. Marozas V, PreikŠA Ž, Koike T, Watanabe T. Impact of nitrogen pollution disturbances on forest vegetation and fungi near a fertilizer factory. *Eurasian Journal of Forest Research* 2022;**22**:52-58. https://doi.org/10.14943/EJFR.22.52

172. Fu C, Qin Y, Xiang Q, Qiao M, Zhu Y. Ph drives the spatial variation of antibiotic resistance gene profiles in riparian soils at a watershed scale. *Environmental Pollution* 2023;**326**:121486.

173. Huang H, Zheng X, Chen Y, Liu H, Wan R, Su Y. Alkaline fermentation of waste sludge causes a significant reduction of antibiotic resistance genes in anaerobic reactors. *Science of the Total Environment* 2017;**580**:380-87.

174. Florent P, Cauchie H-M, Herold M, Jacquet S, Ogorzaly L. Soil ph, calcium content and bacteria as major factors responsible for the distribution of the known fraction of the DNA bacteriophage populations in soils of luxembourg. *Microorganisms* 2022;**10**:1458.

175. Weinheimer AR, Aylward FO, Leray M, Scott JJ. Contrasting drivers of abundant phage and prokaryotic communities revealed in diverse coastal ecosystems. *ISME communications* 2023;**3**:127.

176. Chen L, Xun W, Sun L, Zhang N, Shen Q, Zhang R. Effect of different long-term fertilization regimes on the viral community in an agricultural soil of southern china. *European journal of soil biology* 2014;**62**:121-26.

177. Adriaenssens EM, Kramer R, Van Goethem MW, Makhalanyane TP, Hogg I, Cowan DA. Environmental drivers of viral community composition in antarctic soils identified by viromics. *Microbiome* 2017;**5**:1-14.

178. Saxton MA, Naqvi NS, Rahman F, Thompson CP, Chambers RM, Kaste JM *et al.* Site-specific environmental factors control bacterial and viral diversity in stormwater retention ponds. *Aquatic Microbial Ecology* 2016;**77**:23-36.

179. Liao H, Li H, Duan C-S, Zhou X-Y, Luo Q-P, An X-L *et al.* Response of soil viral communities to land use changes. *Nature Communications* 2022;**13**:6027.

180. Wu H, Yan W, Wu H, Zhang J, Zhang Z, Zhang Z *et al.* Consecutive monoculture regimes differently affected the diversity of the rhizosphere soil viral community and accumulated soil-borne plant viruses. *Agriculture, Ecosystems & Environment* 2022;**337**:108076.

181. Li Y, Sun H, Yang W, Chen G, Xu H. Dynamics of bacterial and viral communities in paddy soil with irrigation and urea application. *Viruses* 2019;**11**:347.

182. Li J, Mau RL, Dijkstra P, Koch BJ, Schwartz E, Liu X-JA *et al.* Predictive genomic traits for bacterial growth in culture versus actual growth in soil. *The ISME journal* 2019;**13**:2162-72.

183. Hungate BA, Mau RL, Schwartz E, Caporaso JG, Dijkstra P, van Gestel N *et al.* Quantitative microbial ecology through stable isotope probing. *Applied and environmental microbiology* 2015;**81**:7570-81.

184. Rousk J, Bååth E. Fungal and bacterial growth in soil with plant materials of different c/n ratios. *FEMS microbiology ecology* 2007;**62**:258-67.

185. Bååth E. Measurement of protein synthesis by soil bacterial assemblages with the leucine incorporation technique. *Biology and Fertility of Soils* 1994;**17**:147-53.
186. Bååth E. Estimation of fungal growth rates in soil using 14c-acetate incorporation into ergosterol. *Soil Biology and Biochemistry* 2001;**33**:2011-18.
187. Demoling F, Figueroa D, Bååth E. Comparison of factors limiting bacterial growth in different soils. *Soil Biology and Biochemistry* 2007;**39**:2485-95.
188. Bååth E. Thymidine incorporation into macromolecules of bacteria extracted from soil by homogenization-centrifugation. *Soil Biology and Biochemistry* 1992;**24**:1157-65.
189. Demoling F, Nilsson LO, Bååth E. Bacterial and fungal response to nitrogen fertilization in three coniferous forest soils. *Soil Biology and Biochemistry* 2008;**40**:370-79.
190. Kamble PN, Rousk J, Frey SD, Bååth E. Bacterial growth and growth-limiting nutrients following chronic nitrogen additions to a hardwood forest soil. *Soil biology and biochemistry* 2013;**59**:32-37.
191. Tobor-Kapłon MA, Bloem J, Römkens PF, Ruiter Pd. Functional stability of microbial communities in contaminated soils. *Oikos* 2005;**111**:119-29.
192. Rousk J, Brookes PC, Bååth E. Contrasting soil ph effects on fungal and bacterial growth suggest functional redundancy in carbon mineralization. *Applied and environmental microbiology* 2009;**75**:1589-96.
193. Malik AA, Puissant J, Buckeridge KM, Goodall T, Jehmlich N, Chowdhury S *et al.* Land use driven change in soil ph affects microbial carbon cycling processes. *Nature Communications* 2018;**9**:3591. https://doi.org/10.1038/s41467-018-05980-1
194. Meidute S, Demoling F, Bååth E. Antagonistic and synergistic effects of fungal and bacterial growth in soil after adding different carbon and nitrogen sources. *Soil Biology and Biochemistry* 2008;**40**:2334-43.

195. Samad MS, Johns C, Richards KG, Lanigan GJ, de Klein CA, Clough TJ *et al.* Response to nitrogen addition reveals metabolic and ecological strategies of soil bacteria. *Molecular ecology* 2017;**26**:5500-14.

196. Stoddard SF, Smith BJ, Hein R, Roller BR, Schmidt TM. Rrn db: Improved tools for interpreting rrna gene abundance in bacteria and archaea and a new foundation for future development. *Nucleic acids research* 2015;**43**:D593-D98.

197. Chen Y, Neilson JW, Kushwaha P, Maier RM, Barberán A. Life-history strategies of soil microbial communities in an arid ecosystem. *The ISME Journal* 2021;**15**:649-57.

198. Vieira-Silva S, Falony G, Darzi Y, Lima-Mendez G, Garcia Yunta R, Okuda S *et al.* Species–function relationships shape ecological properties of the human gut microbiome. *Nature microbiology* 2016;**1**:1-8.

199. Barberán A, Fernández-Guerra A, Bohannan BJ, Casamayor EO. Exploration of community traits as ecological markers in microbial metagenomes. *Molecular ecology* 2012;**21**:1909-17.

200. Morris KA, Richter A, Migliavacca M, Schrumpf MJSB, Biochemistry. Growth of soil microbes is not limited by the availability of nitrogen and phosphorus in a mediterranean oak-savanna. 2022;**169**:108680.

201. Spohn M, Klaus K, Wanek W, Richter A. Microbial carbon use efficiency and biomass turnover times depending on soil depth–implications for carbon cycling. *Soil Biology and Biochemistry* 2016;**96**:74-81.

202. Rousk J, Brookes PC, Bååth EJSB, Biochemistry. Investigating the mechanisms for the opposing ph relationships of fungal and bacterial growth in soil. 2010;**42**:926-34.

203. Fernández-Calviño D, Bååth E. Growth response of the bacterial community to ph in soils differing in ph. *FEMS Microbiology Ecology* 2010;**73**:149-56. https://doi.org/10.1111/j.1574-6941.2010.00873.x %J FEMS Microbiology Ecology

204. Rousk J, Brookes PC, Bååth E. Fungal and bacterial growth responses to n fertilization and ph in the 150-year ‘park grass’ uk grassland experiment. *FEMS Microbiology Ecology* 2011;**76**:89-99. https://doi.org/10.1111/j.1574-6941.2010.01032.x %J FEMS Microbiology Ecology

205. Bååth E, Arnebrant K. Growth rate and response of bacterial communities to ph in limed and ash treated forest soils. *Soil Biology and Biochemistry* 1994;**26**:995-1001.

206. Bååth E. Growth rates of bacterial communities in soils at varying ph: A comparison of the thymidine and leucine incorporation techniques. *Microbial Ecology* 1998;**36**:316-27.

207. Bååth E, Frostegård Å, Fritze H. Soil bacterial biomass, activity, phospholipid fatty acid pattern, and ph tolerance in an area polluted with alkaline dust deposition. *Applied and Environmental Microbiology* 1992;**58**:4026-31.

208. Walsh JJ, Rousk J, Edwards-Jones G, Jones DL, Williams AP. Fungal and bacterial growth following the application of slurry and anaerobic digestate of livestock manure to temperate pasture soils. *Biology and Fertility of Soils* 2012;**48**:889-97.

209. Ji Y, Conrad R, Xu H. Responses of archaeal, bacterial, and functional microbial communities to growth season and nitrogen fertilization in rice fields. *Biology and fertility of soils* 2020;**56**:81-95.

210. Wu L, Yang Y, Chen S, Jason Shi Z, Zhao M, Zhu Z *et al.* Microbial functional trait of rrna operon copy numbers increases with organic levels in anaerobic digesters. *The ISME journal* 2017;**11**:2874-78.

211. Ma X, Wang T, Shi Z, Chiariello NR, Docherty K, Field CB *et al.* Long-term nitrogen deposition enhances microbial capacities in soil carbon stabilization but reduces network complexity. *Microbiome* 2022;**10**:112.

212. Gravuer K, Eskelinen A. Nutrient and rainfall additions shift phylogenetically estimated traits of soil microbial communities. *Frontiers in microbiology* 2017;**8**:258647.

213. He Q, Wu Y, Wang J, Bing H. Community rrna operon copy number of soil bacteria decreases with soil depth and ecosystem succession in postglacial ecosystems. *Applied soil ecology* 2023;**186**:104817.

214. Nemergut DR, Knelman JE, Ferrenberg S, Bilinski T, Melbourne B, Jiang L *et al.* Decreases in average bacterial community rrna operon copy number during succession. *The ISME journal* 2016;**10**:1147-56.

215. Dai T, Wen D, Bates CT, Wu L, Guo X, Liu S *et al.* Nutrient supply controls the linkage between species abundance and ecological interactions in marine bacterial communities. *Nature communications* 2022;**13**:175.

216. Feng T, Su W, Zhu J, Yang J, Wang Y, Zhou R *et al.* Corpse decomposition increases the diversity and abundance of antibiotic resistance genes in different soil types in a fish model. *Environmental Pollution* 2021;**286**:117560.

217. Wang F, Han W, Chen S, Dong W, Hu C, Liu B. Fifteen-year application of manure and chemical fertilizers differently impacts soil args and microbial community structure. *Frontiers in microbiology* 2020;**11**:486880.

218. Wang F, Xu M, Stedtfeld RD, Sheng H, Fan J, Liu M *et al.* Long-term effect of different fertilization and cropping systems on the soil antibiotic resistome. *Environmental science & technology* 2018;**52**:13037-46.

219. Xie W-Y, Yuan S-T, Xu M-G, Yang X-P, Shen Q-R, Zhang W-W *et al.* Long-term effects of manure and chemical fertilizers on soil antibiotic resistome. *Soil Biology and Biochemistry* 2018;**122**:111-19.

220. Sun Y, Qiu T, Gao M, Shi M, Zhang H, Wang X. Inorganic and organic fertilizers application enhanced antibiotic resistome in greenhouse soils growing vegetables. *Ecotoxicology and environmental safety* 2019;**179**:24-30.

221. Yang Y, Jiang X, Chai B, Ma L, Li B, Zhang A *et al.* Args-oap: Online analysis pipeline for antibiotic resistance genes detection from metagenomic data using an integrated structured arg-database. *Bioinformatics* 2016;**32**:2346-51.

222. Du S, Shen J-P, Hu H-W, Wang J-T, Han L-L, Sheng R *et al.* Large-scale patterns of soil antibiotic resistome in chinese croplands. *Science of the total environment* 2020;**712**:136418.

223. Hu HW, Wang JT, Singh BK, Liu YR, Chen YL, Zhang YJ *et al.* Diversity of herbaceous plants and bacterial communities regulates soil resistome across forest biomes. *Environmental microbiology* 2018;**20**:3186-200.

224. Dlugosch L, Poehlein A, Wemheuer B, Pfeiffer B, Giebel H-A, Daniel R *et al.* Nitrogen availability drives gene length of dominant prokaryotes and diversity of genes acquiring nitrogen-species in oceanic systems. *bioRxiv* 2021:2021.01. 10.426031.

225. Liu H, Zhang H, Powell J, Delgado-Baquerizo M, Wang J, Singh B. Warmer and drier ecosystems select for smaller bacterial genomes in global soils. *iMeta* 2023;**2**:e70.

226. Tian J, Dungait JA, Lu X, Yang Y, Hartley IP, Zhang W *et al.* Long-term nitrogen addition modifies microbial composition and functions for slow carbon cycling and increased sequestration in tropical forest soil. *Global change biology* 2019;**25**:3267-81.

227. Su JQ, Ding LJ, Xue K, Yao HY, Quensen J, Bai SJ *et al.* Long-term balanced fertilization increases the soil microbial functional diversity in a phosphorus-limited paddy soil. *Molecular ecology* 2015;**24**:136-50.

228. Ding L-J, Su J-Q, Sun G-X, Wu J-S, Wei W-X. Increased microbial functional diversity under long-term organic and integrated fertilization in a paddy soil. *Applied microbiology and biotechnology* 2018;**102**:1969-82.

229. Bebber DP, Richards VR. A meta-analysis of the effect of organic and mineral fertilizers on soil microbial diversity. *Applied Soil Ecology* 2022;**175**:104450.

230. Ros M, Klammer S, Knapp B, Aichberger K, Insam H. Long-term effects of compost amendment of soil on functional and structural diversity and microbial activity. *Soil use and management* 2006;**22**:209-18.

231. Bei S, Zhang Y, Li T, Christie P, Li X, Zhang J. Response of the soil microbial community to different fertilizer inputs in a wheat-maize rotation on a calcareous soil. *Agriculture, Ecosystems & Environment* 2018;**260**:58-69.

232. Shen W, Lin X, Shi W, Min J, Gao N, Zhang H *et al.* Higher rates of nitrogen fertilization decrease soil enzyme activities, microbial functional diversity and nitrification capacity in a chinese polytunnel greenhouse vegetable land. *Plant and soil* 2010;**337**:137-50.

233. Zhu Z, Bai Y, Lv M, Tian G, Zhang X, Li L *et al.* Soil fertility, microbial biomass, and microbial functional diversity responses to four years fertilization in an apple orchard in north china. *Horticultural plant journal* 2020;**6**:223-30.

234. Kumar U, Shahid M, Tripathi R, Mohanty S, Kumar A, Bhattacharyya P *et al.* Variation of functional diversity of soil microbial community in sub-humid tropical rice-rice cropping system under long-term organic and inorganic fertilization. *Ecological indicators* 2017;**73**:536-43.

235. Li F, Liu M, Li Z, Jiang C, Han F, Che Y. Changes in soil microbial biomass and functional diversity with a nitrogen gradient in soil columns. *Applied Soil Ecology* 2013;**64**:1-6.

236. Liu J, Liu M, Wu M, Jiang C, Chen X, Cai Z *et al.* Soil ph rather than nutrients drive changes in microbial community following long-term fertilization in acidic ultisols of southern china. *Journal of Soils and Sediments* 2018;**18**:1853-64.

237. He Y, Qi Y, Dong Y, Xiao S, Peng Q, Liu X *et al.* Effects of nitrogen fertilization on soil microbial biomass and community functional diversity in temperate grassland in inner mongolia, china. *Clean–Soil, Air, Water* 2013;**41**:1216-21.

238. Zhong W, Gu T, Wang W, Zhang B, Lin X, Huang Q *et al.* The effects of mineral fertilizer and organic manure on soil microbial community and diversity. *Plant and soil* 2010;**326**:511-22.

239. Wang Z, Wang S, Bian T, Song Q, Wu G, Awais M *et al.* Effects of nitrogen addition on soil microbial functional diversity and extracellular enzyme activities in greenhouse cucumber cultivation. *Agriculture* 2022;**12**:1366.

240. Zhang X, Johnston ER, Barberán A, Ren Y, Wang Z, Han X. Effect of intermediate disturbance on soil microbial functional diversity depends on the amount of effective resources. *Environmental microbiology* 2018;**20**:3862-75.

241. Bahram M, Hildebrand F, Forslund SK, Anderson JL, Soudzilovskaia NA, Bodegom PM *et al.* Structure and function of the global topsoil microbiome. *Nature* 2018;**560**:233-37.

242. Song H-K, Shi Y, Yang T, Chu H, He J-S, Kim H *et al.* Environmental filtering of bacterial functional diversity along an aridity gradient. *Scientific Reports* 2019;**9**:866.

243. Guo Y, Chen X, Wu Y, Zhang L, Cheng J, Wei G *et al.* Natural revegetation of a semiarid habitat alters taxonomic and functional diversity of soil microbial communities. *Science of the Total Environment* 2018;**635**:598-606.

244. Lan X, Du H, Peng W, Liu Y, Fang Z, Song T. Functional diversity of the soil culturable microbial community in eucalyptus plantations of different ages in guangxi, south china. *Forests* 2019;**10**:1083.

245. Kerfahi D, Dong K, Yang Y, Kim H, Takahashi K, Adams J. Elevation trend in bacterial functional gene diversity decouples from taxonomic diversity. *Catena* 2021;**199**:105099.
